# Supplementary material for: Two-period e-commerce platforms operation strategies considering the difference in product quality perception
Source: PLoS One. 2026 Mar 26;21(3):e0327280. doi: 10.1371/journal.pone.0327280 (PMC13020826; doi:10.1371/journal.pone.0327280)
Supplement: S1 Appendix — (DOCX) [file pone.0327280.s001.docx]

Appendix

Equilibrium solving

Appendix S1. Proof of equilibrium solution of self-operated model (S-R) under simultaneous decision-making

By comparing consumer second period utility:

$$L-b_{AR}^{S}-tx_{0}=\bar{v}-c_{BR}^{S}-t\left(1-x_{0}\right)+\beta^{k}c_{BR}^{S}$$

$$L-b_{BR}^{S}-t\left(1-x_{1}\right)=\bar{v}-c_{AR}^{S}-tx_{1}+\beta^{k}c_{AR}^{S}$$

The utility indistinguishability points for consumers repeating and switching purchases of product$A$and product$B$in the second period can be obtained as follows:

$$x_{0}=\frac{t-b_{AR}^{S}-\lambda(H-L)-c_{BR}^{S}\left(\beta^{S}-1\right)}{2t}$$

$$x_{1}=\frac{t+b_{BR}^{S}+\lambda(H-L)+c_{AR}^{S}\left(\beta^{S}-1\right)}{2t}$$

According to the decision sequence, substitute the aforementioned utility indifference points into $\pi(E_{AR}^{S})$ and $\pi(E_{BR}^{S})$.At the same time, let $\pi(E_{AR}^{S})$ be a derivative of $b_{AR}^{S}$ and $c_{AR}^{S}$, and $\pi(E_{BR}^{S})$ be a derivative of $b_{BR}^{S}$ and $c_{BR}^{S}$, the result of the differentiation is 0, which leads to:

$$b_{AR}^{S}=\frac{1}{3}\left[\frac{t\left(128-\lambda\left(61+24\lambda\right)\right)}{\left(\lambda-1\right)\left(2\lambda-13\right)}-(H-L)\lambda\right]$$

$$b_{BR}^{S}=\frac{1}{3}\left(\frac{t\left(128-\lambda\left(61+24\lambda\right)\right)}{\left(\lambda-1\right)\left(2\lambda-13\right)}-(H-L)\lambda\right)$$

$$c_{AR}^{S}=\frac{\frac{t\left(\lambda\left(59+24\lambda\right)-115\right)}{\left(\lambda-1\right)\left(2\lambda-13\right)}-(H-L)\lambda}{3\left(\beta^{S}-1\right)}$$

$$c_{BR}^{S}=\frac{\frac{t\left(\lambda\left(59+24\lambda\right)-115\right)}{\left(\lambda-1\right)\left(2\lambda-13\right)}-(H-L)\lambda}{3\left(\beta^{S}-1\right)}$$

The product $A$ has a first-period market demand of $d_{A}=y$ and a second-period market demand of $D_{A}=\lambda\text{y}+\left(1-\lambda\right)x_{0}+\left(1-\lambda\right)\left(x_{1}-y\right)$, while the product$B$has a first-period market demand of $d_{B}=\left(1-\text{y}\right)$ and a second-period market demand of$D_{B}=\lambda\text{y}+(1-\lambda)(1-x_{1})$ $+\left(1-\lambda\right)\left(y-x_{0}\right)$. By comparing the two-period utility of consumers at, we have:

$$\bar{v}-c_{BR}^{S}-t\left(1-x_{0}\right)+\beta^{S}c_{BR}^{S}+\bar{v}-a_{AR}^{S}-td_{A}=(\bar{v}-c_{AR}^{S}-tx_{1}+\beta^{S}c_{AR}^{S})+(\bar{v}-a_{BR}^{S}-td_{B})$$

According to the Nash equilibrium, the point of no difference in utility between the two products purchased in the first period is obtained:

$$\text{y}=\frac{t\left(\lambda-2\right)-a_{AR}^{S}\left(\lambda-1\right)+a_{BR}^{S}\left(\lambda-1\right)}{2t\left(\lambda-2\right)}$$

Based on the order of decision, substituting $y$ into $\Pi(E_{AR}^{S})$ and $\Pi(E_{BR}^{S})$, and simultaneously making $\Pi(E_{AR}^{S})$ the derivative of $a_{AR}^{S}$, $\Pi(E_{BR}^{S})$ the derivative of$a_{BR}^{S}$, and the result of the derivation is $0$, we have:

$$a_{AR}^{S}=\frac{2}{3}\left((H-L)\lambda+\frac{t\left(90-\lambda\left(67+7\lambda\right)\right)}{\left(\lambda-1\right)\left(2\lambda-13\right)}\right)$$

$$a_{BR}^{S}=\frac{2}{3}\left((H-L)\lambda+\frac{t\left(90-\lambda\left(67+7\lambda\right)\right)}{\left(\lambda-1\right)\left(2\lambda-13\right)}\right)$$

Substituting the product prices$a_{AR}^{S}$,$a_{BR}^{S}$,$b_{AR}^{S}$,$b_{BR}^{S}$,$c_{AR}^{S}$ and $c_{BR}^{S}$ into $\Pi(M_{AR}^{S})$ and $\Pi(M_{BR}^{S})$, and simultaneously making $\Pi(M_{AR}^{S})$ the derivative of $\omega_{A}^{S}$, $\Pi(M_{BR}^{S})$ the derivative of $\omega_{B}^{S}$, and the result of the derivation is $0$, we have:

$$\omega_{A}^{S}=\frac{t\left(34-\lambda\left(19+8\lambda\right)\right)}{\left(\lambda-1\right)\left(2\lambda-13\right)}$$

$$\omega_{B}^{S}=\frac{t\left(34-\lambda\left(19+8\lambda\right)\right)}{\left(\lambda-1\right)\left(2\lambda-13\right)}$$

Observation of the equilibrium results shows that:

$b_{AR}^{S}=b_{BR}^{S}\text{、}c_{AR}^{S}=c_{BR}^{S}\text{、}a_{AR}^{S}=a_{BR}^{S}\text{、}\omega_{A}^{S}=\omega_{B}^{S}$, i.e., the optimal solutions of the platform $E_{A}\text{、}E_{B}$ are all the same in the S-R case.

Appendix S2. Proof of equilibrium solution of commission-type model (S-C) under simultaneous decision making

By comparing consumer second period utility:

$$L-b_{AC}^{S}-tx_{0}=\bar{v}-c_{BC}^{S}-t\left(1-x_{0}\right)+e_{i}^{k}$$

It can be obtained that there is no point of difference between the utility of consumers in the second period for repeat and switching purchases of the product $A$ and the product $B$:

$$x_{0}=\frac{t-(H-L)\lambda-b_{AC}^{S}+c_{BC}^{S}-e_{B}^{S}}{2t}$$

$$x_{1}=\frac{t+(H-L)\lambda+b_{BC}^{S}-c_{AC}^{S}+e_{A}^{S}}{2t}$$

Based on the decision order, the above utility indistinguishable points are obtained by substituting them into $\pi(M_{AC}^{S})$ and $\pi(M_{BC}^{S})$ and at the same time making$\pi(M_{AC}^{S})$ the derivative of $b_{AC}^{S}$ and $c_{AC}^{S}$, and $\pi(M_{BC}^{S})$ the derivative of $b_{BC}^{S}$and $c_{BC}^{S}$ , which results in $0$:

$$b_{AC}^{S}=b_{BC}^{S}=\frac{\lambda\left(H-L\right)\left(\lambda-1\right)\left(\lambda\left(785+28\lambda\right)-1574\right)+5t\left(\lambda\left(2207+100\lambda\right)-3842\right)}{12\left(1-\lambda\right)\left(\lambda\left(95+4\lambda\right)-512\right)}$$

$$c_{AC}^{S}=c_{BC}^{S}=\frac{\left(\lambda\left(H-L\right)\left(1-\lambda\right)\left(127+4\lambda\right)\left(2+5\lambda\right)\right)+t\left(20750-\lambda\left(14309+652\lambda\right)\right)}{12\left(\lambda-1\right)\left(\lambda\left(95+4\lambda\right)-512\right)}$$

Substituting product prices$b_{AC}^{S}$,$b_{BC}^{S}$,$c_{AC}^{S}$, and $c_{BC}^{S}$ into $\pi(E_{AC}^{S})$ and$\pi(E_{BC}^{S})$, and simultaneously making $\pi(E_{AC}^{S})$ the derivative of $e_{A}^{S}$ and $\pi(E_{BC}^{S})$ the derivative of $e_{B}^{S}$, the result is $0$, which can be obtained:

$$e_{A}^{S}=e_{B}^{S}=\frac{3t\left(598-7\lambda\left(87+4\lambda\right)\right)-3\lambda\left(H-L\right)\left(\lambda-1\right)\left(\lambda\left(103+4\lambda\right)-378\right)}{4\left(\lambda-1\right)\left(\lambda\left(95+4\lambda\right)-512\right)}$$

By comparing the consumers' two-period utility it can be obtained that there is no point of difference between the utility of the consumers who buy the two products in the first period.

$$\bar{v}-c_{BC}^{S}-t\left(1-x_{0}\right)+e_{B}^{S}+(\bar{v}-a_{AC}^{S}-td_{A})=(\bar{v}-c_{AC}^{S}-tx_{1}+e_{A}^{S})+(\bar{v}-a_{BC}^{S}-td_{B})$$

$$\text{y}=\frac{6t\left(\lambda-2\right)-4\left(\lambda-1\right)a_{AC}^{S}+4\left(\lambda-1\right)a_{BC}^{S}-3\left(\lambda-1\right)\theta_{A}^{S}+3\left(\lambda-1\right)\theta_{B}^{S}}{12t\left(\lambda-2\right)}$$

Substituting $y$ into $\Pi(M_{AC}^{S})$ and $\Pi(M_{BC}^{S})$, and at the same time making$\Pi(M_{AC}^{S})$ the derivative of $a_{AC}^{S}$ and $\Pi(M_{BC}^{S})$ the derivative of $a_{BC}^{S}$, the result is$0$, we have:

$$a_{AC}^{S}=a_{BC}^{S}=\frac{2\lambda\left(H-L\right)\left(1-\lambda\right)\left(292+\lambda\left(143+4\lambda\right)\right)+t\left(17416+\lambda\left(+\lambda\left(629+44\lambda\right)-14840\right)\right)}{6\left(\lambda-1\right)\left(\lambda\left(95+4\lambda\right)-512\right)}$$

The product prices and discount amount $e_{i}^{S}$ can be obtained by substituting $\Pi(E_{AC}^{S})$ and $\Pi(E_{BC}^{S})$, and at the same time making $\Pi(E_{AC}^{S})$ the derivative of$\theta_{A}^{S}$,$\Pi(E_{BC}^{S})$ the derivative of $\theta_{B}^{S}$, and the result of the derivation is $0$:

$$\theta_{A}^{S}=\theta_{B}^{S}=\frac{\left(\lambda\left(H-L\right)\left(1-\lambda\right)\left(\lambda\left(119+4\lambda\right)-110\right)\right)+t\left(2818-\lambda\left(2017+92\lambda\right)\right)}{2\left(\lambda-1\right)\left(\lambda\left(95+4\lambda\right)-512\right)}$$

Observation of the equilibrium results shows that:

$a_{AC}^{S}=a_{BC}^{S}\text{、}b_{AC}^{S}=b_{BC}^{S}\text{、}c_{AC}^{S}=c_{BC}^{S}\text{、}e_{A}^{S}=e_{B}^{S}\text{、}\theta_{A}^{S}=\theta_{B}^{S}$, i.e., the optimal solutions of the platform $E_{A}\text{、}E_{B}$ are all the same in the S-C case.

Appendix S3. Proof of equilibrium solution of self-employed model (D-R) under sequential decision making

The order of solution is the same as Appendix A.1, with the difference that in the stage of platform retail price competition, platform $E_{A}$ as the leader platform makes the decision first, and platform $E_{B}$ as the follower platform makes the decision later. The equilibrium solutions of the Stackelberg model using backward induction are as follows:

$$\omega_{A}^{D}=\frac{\left(\lambda\left(H-L\right)\left(1-\lambda\right)\left(3+2\lambda\right)\right)+t\left(899+\lambda\left(4\left(\lambda-41\right)\lambda-545\right)\right)}{3\left(\lambda-1\right)\left(4\lambda\left(5+\lambda\right)-95\right)}$$

$$\omega_{B}^{D}=\frac{1021t+\lambda\left(H-L\right)\left(\lambda-1\right)\left(3+2\lambda\right)-t\lambda\left(607+4\lambda\left(55+\lambda\right)\right)}{3\left(\lambda-1\right)\left(4\lambda\left(5+\lambda\right)-95\right)}$$

$$\begin{matrix} a_{AR}^{D}=\frac{\lambda(H-L)(\lambda-1)(72705+2\lambda(\lambda(4\lambda(628+83\lambda)-4527)-29369))}{192(\lambda-1)(\lambda(3+\lambda)-5)(4\lambda(5+\lambda)-95)}+ \\ \frac{\text{t}(\lambda(779939+2\lambda(\lambda(972\lambda^{2}+8\lambda^{3}-918\lambda-60069)-57735)))-561665}{192(\lambda-1)(\lambda(3+\lambda)-5)(4\lambda(5+\lambda)-95)} \end{matrix}$$

$$a_{BR}^{D}=\frac{11147t-\frac{11558t}{\lambda-1}+71\lambda\left(59H-59L+2t\right)+\frac{142\left(17t+\lambda\left(L-H+4t\right)\right)}{\lambda\left(3+\lambda\right)-5}-\frac{24\left(497\lambda\left(L-H\right)+4t\left(18446+4589\lambda\right)\right)}{4\lambda\left(5+\lambda\right)-95}}{6816}$$

$$\begin{matrix} b_{BR}^{D}=\frac{t\left(195450+\lambda\left(\lambda\left(2\lambda\left(21133+3898\lambda+68\lambda^{2}\right)-13833\right)-221737\right)\right)}{96\left(1-\lambda\right)\left(\lambda\left(3+\lambda\right)-5\right)\left(4\lambda\left(5+\lambda\right)-95\right)}+ \\ \frac{t\left(195450+\lambda\left(\lambda\left(2\lambda\left(21133+3898\lambda+68\lambda^{2}\right)-13833\right)-221737\right)\right)}{96\left(1-\lambda\right)\left(\lambda\left(3+\lambda\right)-5\right)\left(4\lambda\left(5+\lambda\right)-95\right)} \end{matrix}$$

$$\begin{matrix} b_{AR}^{D}=\frac{\left(\text{λ}\left(\text{H-L}\right)\left(\text{1-λ}\right)\left(\text{11145+λ}\left(\text{4λ}\left(\text{3λ}\left(\text{67+8λ}\right)\text{-292}\right)\text{-9379}\right)\right)\right)}{48\left(\lambda-1\right)\left(\lambda\left(3+\lambda\right)-5\right)\left(4\lambda\left(5+\lambda\right)-95\right)}+ \\ \frac{t\left(\lambda\left(109188+\lambda\left(3293+2\lambda\left(2\lambda\left(18\lambda-653\right)-9485\right)\right)\right)-98215\right)}{48\left(\lambda-1\right)\left(\lambda\left(3+\lambda\right)-5\right)\left(4\lambda\left(5+\lambda\right)-95\right)} \end{matrix}$$

$$\begin{matrix} c_{AR}^{D}=\frac{\left(\lambda\left(H-L\right)\left(1-\lambda\right)\left(10890+\lambda\left(4\lambda\left(3\lambda\left(61+8\lambda\right)-341\right)-8753\right)\right)\right)}{48\left(\beta^{D}-1\right)\left(\lambda-1\right)\left(\lambda\left(3+\lambda\right)-5\right)\left(4\lambda\left(5+\lambda\right)-95\right)}+ \\ \frac{t\left(90970+\lambda\left(\lambda\left(2\lambda\left(9971+2\left(683-18\lambda\right)\lambda\right)-719\right)-108459\right)\right)}{48\left(\beta^{D}-1\right)\left(\lambda-1\right)\left(\lambda\left(3+\lambda\right)-5\right)\left(4\lambda\left(5+\lambda\right)-95\right)} \end{matrix}$$

$$\begin{matrix} c_{BR}^{D}=\frac{\left(\lambda\left(H-L\right)\left(1-\lambda\right)\left(11925+\lambda\left(4\lambda\left(\lambda\left(209+24\lambda\right)-292\right)-9859\right)\right)\right)}{96\left(\beta^{D}-1\right)\left(\lambda-1\right)\left(\lambda\left(3+\lambda\right)-5\right)\left(4\lambda\left(5+\lambda\right)-95\right)}+ \\ \frac{t\left(177125+\lambda\left(\lambda\left(2\lambda\left(19299+3670\lambda+68\lambda^{2}\right)-14603\right)-198312\right)\right)}{96\left(\beta^{D}-1\right)\left(\lambda-1\right)\left(\lambda\left(3+\lambda\right)-5\right)\left(4\lambda\left(5+\lambda\right)-95\right)} \end{matrix}$$

Appendix S4. Proof of equilibrium solution of commission-type model (D-C) under sequential decision-making

The order of solution is the same as Appendix A.2, with the difference that platform $E_{A}$ as the leader platform makes the decision first and platform $E_{B}$ as the follower platform makes the decision later in the phase of platform discount competition in the second period and the phase of platform commission rate competition in the first period. The equilibrium solutions of the Stackelberg model using backward induction are as follows:

$$\begin{matrix} a_{AC}^{D}=(((H-L)(1-\lambda)\lambda(\lambda(531431374384+\lambda(278396126768+\lambda(\lambda(4\lambda(4\lambda(418961 \\ +4\lambda(5041+56\lambda))-38673485)-6733603987)-47198747420)))-1022023303312)) \\ +t(-33150803072688+\lambda(56507026168288+\lambda(\lambda(2255244859288+\lambda\text{（}255864960371 \\ +\lambda\text{（}32\lambda\text{（}\lambda\text{（}3359+40\lambda\text{（}757+11\lambda))-33429575)-12444370925)))-27188422935136)) \\ -33150803072688))/(6(\lambda-1)(854387017616+\lambda(\lambda(216667089416+\lambda(6230316280+ \\ \lambda(16\lambda(2\lambda(28903+8\lambda(391+5\lambda))-5547017)-1895596843)))-949817488736))) \end{matrix}$$

$$\begin{matrix} a_{BC}^{D}=(((H-L)(1-\lambda)\lambda(\lambda(533060839504+\lambda(270438966704+\lambda(\lambda(4\lambda(4\lambda(509303 \\ +4\lambda(5311+56\lambda))-27078659)-6057290545)-43930520324)))-1019906049712)) \\ +t(\lambda(56839435942528+\lambda(\lambda(2218476834004+\lambda(255051057845+\lambda(32\lambda(\lambda(3899 \\ +40\lambda(757+11\lambda))-33364964)-12404896709)))-27511199806624))-3296852678 \\ 5488))/(6(\lambda-1)(854387017616+\lambda(\lambda(216667089416+\lambda(6230316280+\lambda(16\lambda(2\lambda\\ (28903+8\lambda(391+5\lambda))-5547017)-1895596843)))-949817488736))) \end{matrix}$$

$$\begin{matrix} b_{AC}^{D}=(\left(H-L\right)\left(\lambda-1\right)\lambda(638781615208+\lambda(\lambda\text{ }(318882388426+\lambda(\lambda(16\lambda(2\lambda(212267+ \\ 8\lambda(1160+13\lambda))-4238899)-3383206091)-5115392899))-891241448548))+t(955 \\ 1765639720+\lambda(\lambda(5269008760574+\lambda(86785414771+8\lambda(8\lambda(4\lambda(93785+\lambda(11365+ \\ 152\lambda))-39407831)-6698702351)))-14226916556060)))/(3\left(1-\lambda\right)(854387017616+ \\ \lambda(\lambda\text{ }(216667089416+\lambda(6230316280+\lambda(16\lambda(2\lambda(28903+8\lambda(391+5\lambda))-5547017- \\ 1895596843)))-949817488736))) \end{matrix}$$

$$\begin{matrix} b_{BC}^{D}=(((H-L)(1-\lambda)\lambda(645486251608+\lambda(\lambda(340129529470+\lambda(\lambda(8\lambda(8\lambda(14\lambda(151+2\lambda) \\ -1097)-26757551)-4879041347)-9344291641))-913194084268)))+t(\lambda(129453983 \\ 85620+\lambda(\lambda(10812994871-8\lambda(\lambda(4\lambda(982885+4\lambda(24425+316\lambda))-315356287)-7312 \\ 402544))-4752960387626))-8974557396920))/(3(\lambda-1)(854387017616+\lambda(\lambda(21666 \\ 7089416+\lambda(6230316280+\lambda(16\lambda(2\lambda(28903+8\lambda(391+5\lambda))-5547017)-1895596843))) \\ -949817488736))) \end{matrix}$$

$$\begin{matrix} c_{AC}^{D}=(((H-L)(1-\lambda)\lambda(\lambda(\lambda(188854260884+\lambda(\lambda(8\lambda(8\lambda(108599+4102\lambda+44\lambda^{2}) \\ -2600359)-2824281895)-16107104654))-105764324168)-133569676768)+ \\ t(11033105617600+\lambda(\lambda(6582578652940+\lambda(81052145366+\lambda(8\lambda(16\lambda(253367+ \\ \lambda(29821+398\lambda))-407697857)-70328683919)))-17021113691608)))/(3(\lambda-1) \\ (854387017616+\lambda(\lambda(216667089416+\lambda(6230316280+\lambda(16\lambda(2\lambda(28903+8\lambda(391 \\ +5\lambda))-5547017)-1895596843)))-949817488736)))) \end{matrix}$$

$$\begin{matrix} c_{BC}^{D}=(((H-L)(1-\lambda)\lambda(\lambda(\lambda(213761409572+\lambda(\lambda(32\lambda(\lambda(7537+40\lambda(80+\lambda)) \\ -5025862)-4193432095)-19089212774))-140883448808)-118984151968)) \\ +t(\lambda(15321222294328+\lambda(\lambda(12072655186+\lambda(75222109847-32\lambda(\lambda(1239545 \\ +8\lambda(15752+205\lambda))-102151112)))-6148175618044))-9777424528000))/(3(\lambda-1) \\ (854387017616+\lambda(\lambda(216667089416+\lambda(6230316280+\lambda(16\lambda(2\lambda(28903+8\lambda\\ (391+5\lambda))-5547017)-1895596843)))-949817488736))) \end{matrix}$$

$$\begin{matrix} e_{AC}^{D}=(((H-L)(1-\lambda)\lambda(467907506528+\lambda(\lambda(147827416844+\lambda(1785266974 \\ +\lambda(8\lambda(8\lambda(35429+2\lambda(869+10\lambda))-4614529)-1306174489)))-554396660216)) \\ +t(1194023520448+\lambda(\lambda(881839729108+\lambda(19611283226+\lambda(8\lambda(16\lambda(25487+ \\ \lambda(3643+50\lambda))-52182263)-8437427009)))-2075352184552)))/((\lambda-1) \\ (854387017616+\lambda(\lambda(216667089416+\lambda(6230316280+\lambda(16\lambda(2\lambda(28903+ \\ 8\lambda(391+5\lambda))-5547017)-1895596843))))-949817488736))) \end{matrix}$$

$$\begin{matrix} e_{BC}^{D}=(3(H-L)(1-\lambda)\lambda(157485226976+\lambda(\lambda(52436212756+\lambda(102349714 \\ +\lambda(32\lambda(\lambda(8\lambda(72+\lambda)-1907)-942314)-620917491)))-188781479112)) \\ +3t(\lambda(486491942104+\lambda(\lambda(5625810602+\lambda(3396382379-32\lambda(\lambda(62293 \\ +8\lambda(704+9\lambda))-4312788)))-229739973900))-267489017216))/((\lambda-1) \\ (854387017616+\lambda(\lambda(216667089416+\lambda(6230316280+\lambda(16\lambda(2\lambda(28903 \\ +8\lambda(391+5\lambda))-5547017)-1895596843)))-949817488736))) \end{matrix}$$

$$\begin{matrix} \theta_{AC}^{D}=(4(H-L)(1-\lambda)\lambda(\lambda(119+4\lambda)-110)(\lambda(169584228+\lambda(4\lambda(\lambda(7173+8\lambda(123+2\lambda)) \\ -411802)-14744913))-189964676)-4t(764195571128+\lambda(\lambda(466526148878+\lambda\\ (6291464287+\lambda(4\lambda(4\lambda(139427+4\lambda(4183+56\lambda))-57485015)-4922881933))) \\ -1193529117260)))/((\lambda-1)(854387017616+\lambda(\lambda(216667089416+\lambda(6230316280+ \\ \lambda(16\lambda(2\lambda(28903+8\lambda(391+5\lambda))-5547017)-1895596843)))-949817488736))) \end{matrix}$$

$$\begin{matrix} \theta_{BC}^{D}=(2(H-L)(1-\lambda)\lambda(\lambda(95+4\lambda)-512)(\lambda(119+4\lambda)-110)(784516+\lambda(\lambda(8\lambda(47+2\lambda) \\ -28479)-575396))+2t(\lambda(2097587265080+\lambda(\lambda(3618760874+\lambda(10676407123-16\lambda\\ (2\lambda(178303+232\lambda(77+\lambda))-28772369)))-854674442468))-1322486447456))/ \\ ((\lambda-1)(854387017616+\lambda(\lambda(216667089416+\lambda(6230316280+\lambda(16\lambda(2\lambda(28903+8\lambda\\ (391+5\lambda))-5547017)-1895596843)))-949817488736))) \end{matrix}$$

Proofs of Theorems

Appendix S5. Proof of Theorem 1

Part(ⅰ): Simultaneous decision-making on both platforms in a commission-based model

The first period retail price of the manufacturer's product is obtained by taking the partial derivative of $\lambda$ :

$$\begin{matrix} \frac{\delta a_{iC}^{S}}{\delta\lambda}=(3t(991144+\lambda(\lambda(275743+8\lambda(2721+62\lambda))-841872))-2(\lambda-1)^{2} (\lambda(\lambda(6273+ \\ 8\lambda(95+2\lambda))-146432)-149504)\triangle s)/6(\lambda-1)^{2} (\lambda(95+4\lambda)-512)^{2} \end{matrix}$$

This gives$6\left(\lambda-1\right)^{2}\left(\lambda\left(95+4\lambda\right)-512\right)^{2}>0$, which makes $\frac{t}{H-L}=f$. By simplifying the molecular part, we have:

$$\begin{matrix} (3t(991144+\lambda(\lambda(275743+8\lambda(2721+62\lambda))-841872))-2\left(\lambda-1\right)^{2}(\lambda(\lambda(6273+8\lambda(95+2\lambda)) \\ -146432)-149504)\triangle\text{s)}/(H-L)=3\text{f}(991144+\lambda(\lambda(275743+8\lambda(2721+62\lambda))-841872)) \\ -2\left(\lambda-1\right)^{2}(\lambda(\lambda(6273+8\lambda(95+2\lambda))-146432)-149504) \end{matrix}$$

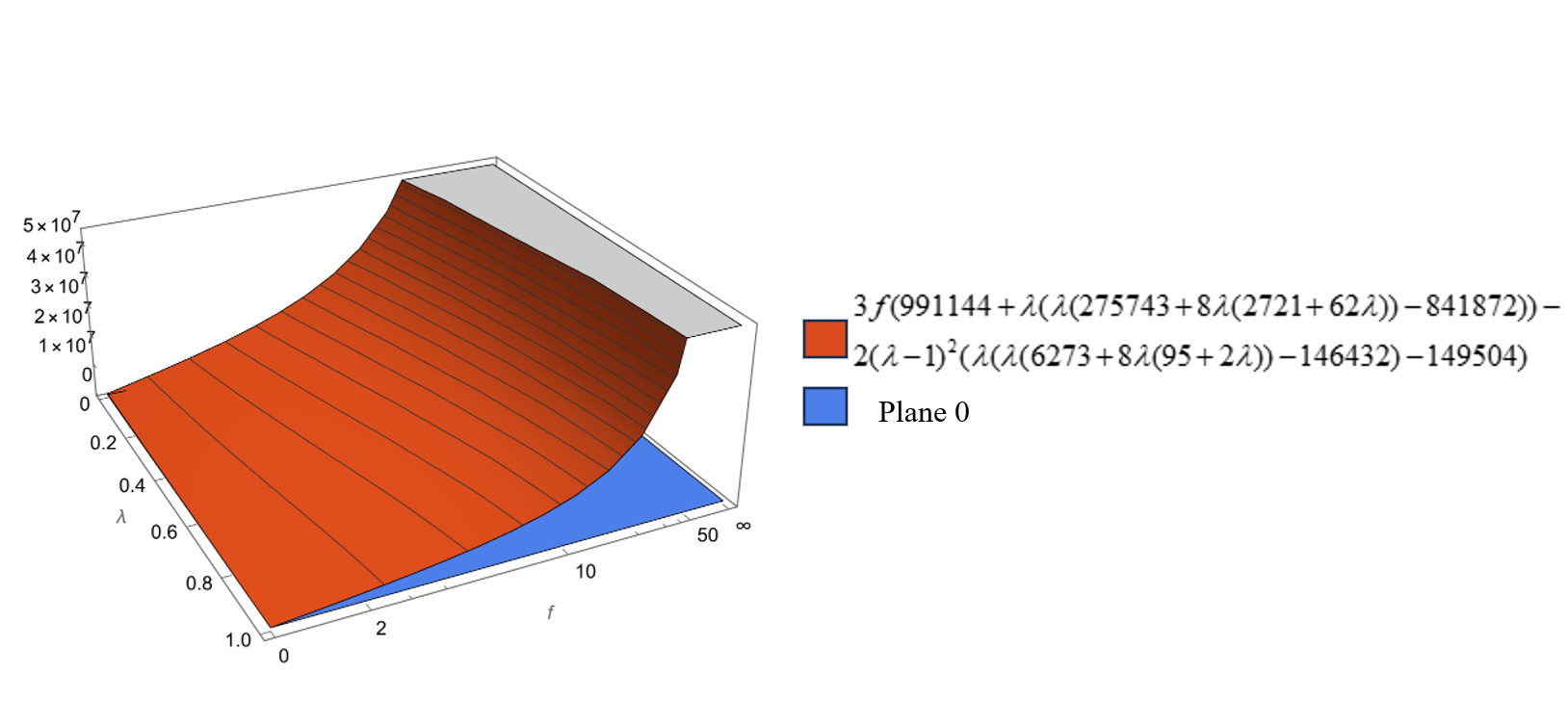


**Fig S1.** Comparison of the size of plane 1 and plane 0

From Fig S1, we have$\frac{\partial a_{iC}^{S}6\left(\lambda-1\right)^{2}\left(\lambda\left(95+4\lambda\right)-512\right)^{2}}{\partial\lambda\left(H-L\right)}>0$ when$0<\lambda<1$, and we can get $\frac{\partial a_{iC}^{S}}{\partial\lambda}>0$. Similarly, we have $\frac{\partial b_{iC}^{S}}{\partial\lambda}>0$and$\frac{\partial c_{iC}^{S}}{\partial\lambda}>0$.

Part(ⅱ): Price changes on leader platforms under a commission-based model

In the commission-based model, the platform acts as a leader when the manufacturer's retail price of the product $a_{AC}^{D}\text{、}b_{AC}^{D}\text{、}c_{AC}^{D}$ is obtained by taking the partial derivative of $\lambda$ respectively, and the proof procedure is shown in Part(ⅰ):

$$\frac{\partial a_{AC}^{D}}{\partial\lambda}>0\text{、}\frac{\partial b_{AC}^{D}}{\partial\lambda}>0\text{、}\frac{\partial c_{AC}^{D}}{\partial\lambda}>0$$

Part(ⅲ): Price changes on follower platforms in a commission-based model

In the commission-based model, when the platform acts as a follower, the proof of the procedure shown in Part(ⅰ) can be obtained by solving for the bias of $\lambda$ on the retail price of the manufacturer's product $a_{BC}^{D}\text{、}b_{BC}^{D}\text{、}c_{BC}^{D}$, respectively:

$$\frac{\partial a_{BC}^{D}}{\partial\lambda}>0\text{、}\frac{\partial b_{BC}^{D}}{\partial\lambda}>0\text{、}\frac{\partial c_{BC}^{D}}{\partial\lambda}>0$$

The Proof of the Theorem is hence completed.

Appendix S6. Proof of Theorem 2

Part(ⅰ): Simultaneous decision-making on both platforms in a self-managed model

The partial derivative of $\lambda$ with respect to the first period retail price of the platform's products gives:

$$\frac{\partial a_{iR}^{S}}{\partial\lambda}=\frac{2}{3}\left(\frac{t\left(479+\lambda\left(239\lambda-542\right)\right)}{\left(13-15\lambda+2\lambda^{2}\right)^{2}}+\triangle\text{s}\right)$$

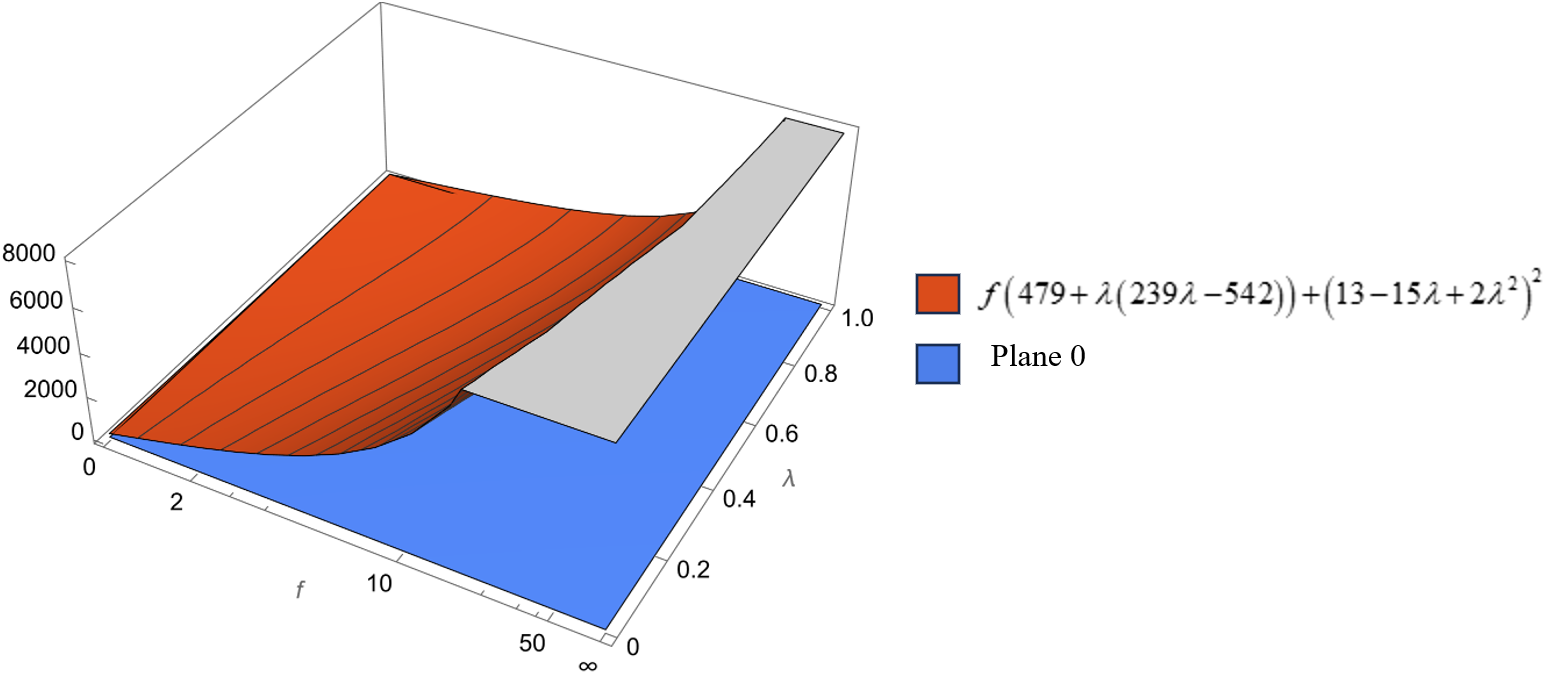


**Fig S2.** Comparison of the size of plane 2 and plane 0

Let$\frac{t}{H-L}=f$, then$\frac{\partial a_{iR}^{S}}{\partial\lambda\triangle\text{s}}=\frac{2}{3}\left(\frac{f\left(479+\lambda\left(239\lambda-542\right)\right)}{\left(13-15\lambda+2\lambda^{2}\right)^{2}}+1\right)$. From Fig S2, we have$f\left(479+\lambda\left(239\lambda-542\right)\right)+\left(13-15\lambda+2\lambda^{2}\right)^{2}>0$when$0<\lambda<1$,i.e.,$\frac{\partial a_{iR}^{S}}{\partial\lambda\triangle\text{s}}>0$, and $\frac{\partial a_{iR}^{S}}{\partial\lambda}>0$when $\triangle\text{s}>0$, and similarly $\frac{\partial b_{iR}^{S}}{\partial\lambda}>0$.


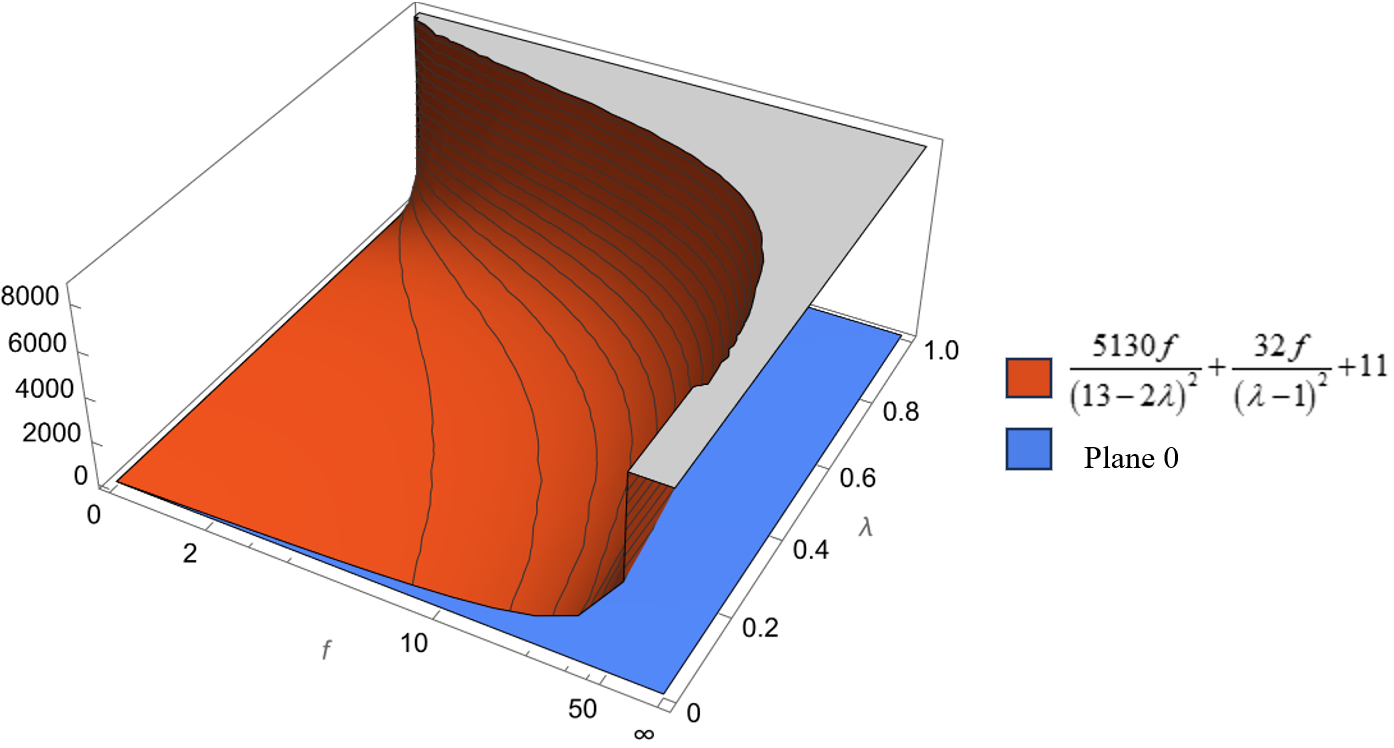


**Fig S3.** Comparison of the size of plane 3 and plane 0

$\frac{\partial c_{iR}^{S}}{\partial\lambda}=\frac{\frac{5130t}{\left(13-2\lambda\right)^{2}}+\frac{32t}{\left(\lambda-1\right)^{2}}+11\triangle\text{s}}{33\left(1-\beta^{S}\right)}$, and$0<\beta^{S}<1$, it is easy to obtain the denominator

part of the partial derivative result$33\left(1-\beta^{S}\right)>0$, so that$\frac{t}{H-L}=f$,$\left(\frac{5130t}{\left(13-2\lambda\right)^{2}}+\frac{32t}{\left(\lambda-1\right)^{2}}+11\triangle\text{s}\right)\text{*}\frac{1}{\triangle\text{s}}=\frac{5130f}{\left(13-2\lambda\right)^{2}}+\frac{32f}{\left(\lambda-1\right)^{2}}+11$.

From Fig S3, the plane$\frac{5130f}{\left(13-2\lambda\right)^{2}}+\frac{32f}{\left(\lambda-1\right)^{2}}+11>0$when$0<\lambda<1$, that is,$\frac{\partial c_{iR}^{S}}{\partial\lambda\triangle\text{s}}>0$.we have $\frac{\partial c_{iR}^{S}}{\partial\lambda}>0$.

Part(ⅱ): Changes in the price of the leader's platform under the self-employed model

In the self-managed model, when the platform acts as the leader, the proof of the partial derivation of $\lambda$ for the retail price of the platform's product $a_{AR}^{D}\text{、}b_{AR}^{D}\text{、}c_{AR}^{D}$ is shown in Part(ⅰ), which leads to$\frac{\partial a_{AR}^{D}}{\partial\lambda}>0\text{、}\frac{\partial b_{AR}^{D}}{\partial\lambda}>0\text{、}\frac{\partial c_{AR}^{D}}{\partial\lambda}>0$.

Part(ⅲ): Price changes on follower platforms in the captive model

In the self-owned type model, when the platform acts as a follower, the partial derivative of $\lambda$ is obtained for the retail price of the platform's product$a_{BR}^{D}\text{、}b_{BR}^{D}\text{、}c_{BR}^{D}$ .The proof procedure is shown in Part(ⅰ) , which leads to $\frac{\partial a_{BR}^{D}}{\partial\lambda}>0\text{、}\frac{\partial b_{BR}^{D}}{\partial\lambda}>0\text{、}\frac{\partial c_{BR}^{D}}{\partial\lambda}>0$. The proof is complete.

Appendix S7. Proof of Theorem 3

Plotting the image with the help of Mathematica scientific computing software, the denominator part of the result of making difference is greater than 0 as can be seen in Fig S4.

**
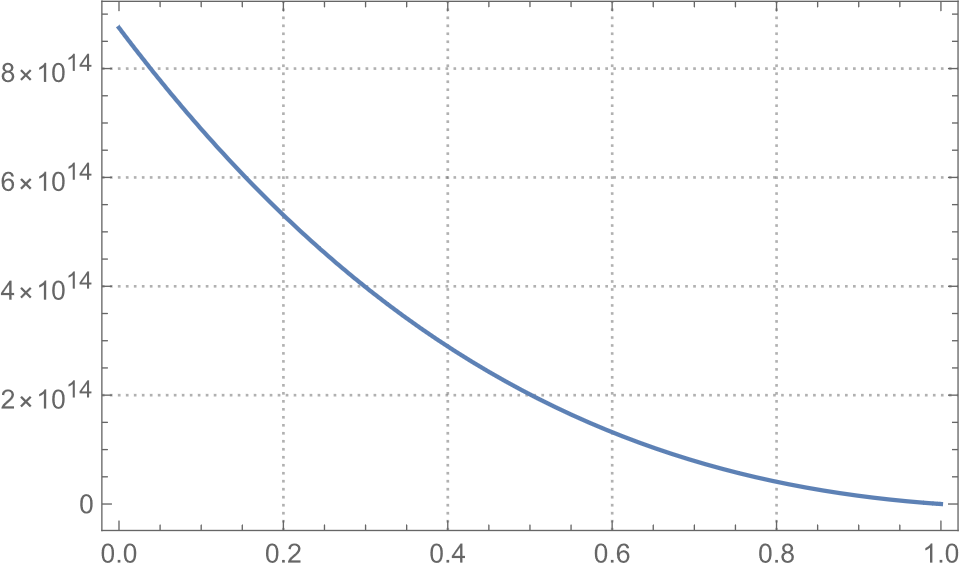
**

**Fig S4.**$\text{ θ}_{\text{i}}^{\text{S}}\text{-θ}_{\text{A}}^{\text{D}}$positive and negative judgement of the denominator part


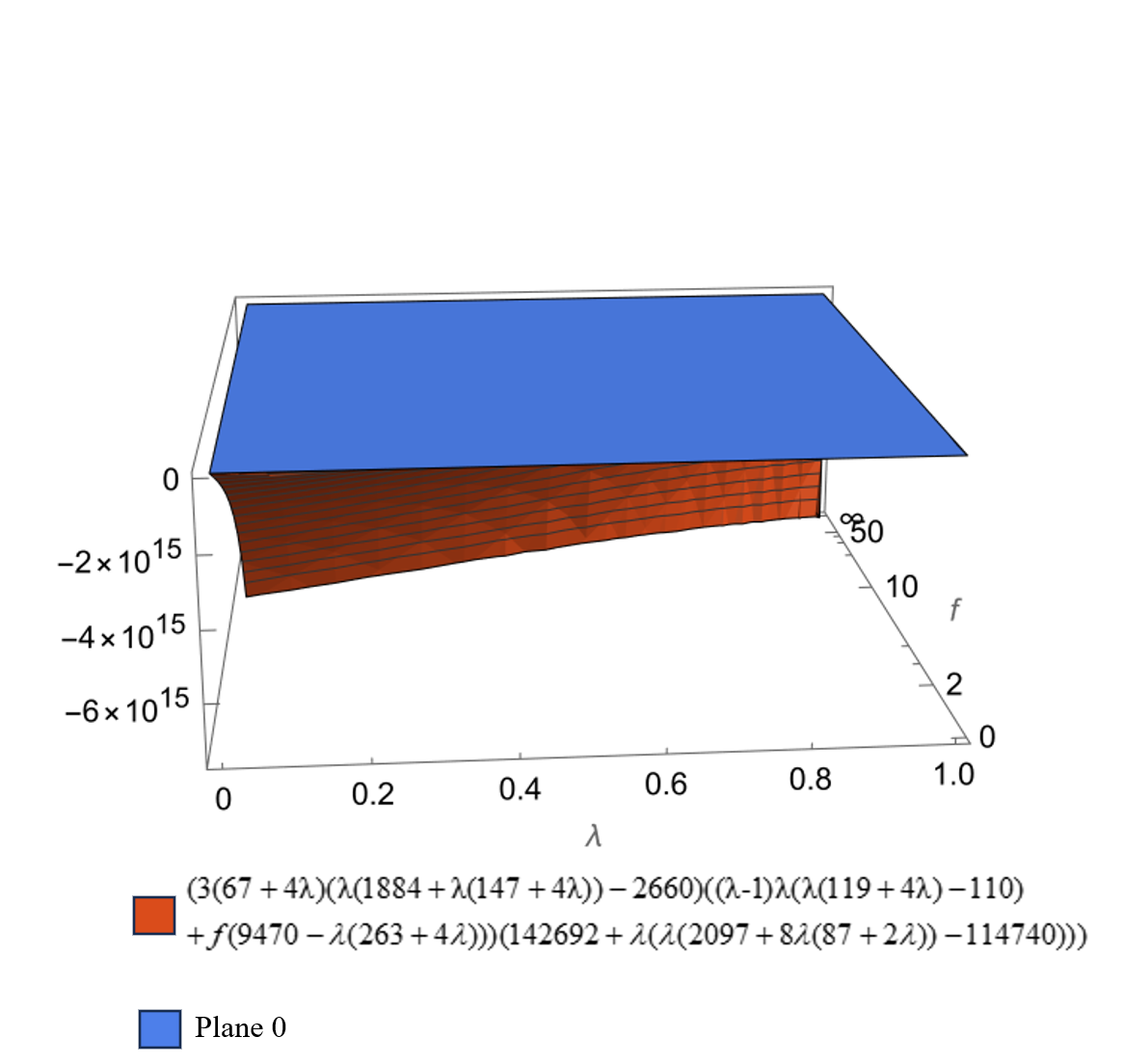


**Fig S5.** Comparison of the size of plane 4 and plane 0

we have $\triangle\text{s}>0\text{、}f>0$ when $H-L=\triangle\text{s}$ and $\frac{t}{H-L}=f$.

$$(3(67+4\lambda)(\lambda\text{（}1884+\lambda\text{（}147+4\lambda))-2660)(\text{Δs}(\lambda-1)\lambda(\lambda(119+4\lambda))-110+t(9470$$

$$\begin{matrix} -\lambda(263+4\lambda)))(142692+\lambda(\lambda(2097+8\lambda(87+2\lambda))-114740)))/\triangle\text{s}=(3(67+4\lambda)(\lambda(1884+ \\ \lambda(147+4\lambda))-2660)((\lambda\text{-1})\lambda(\lambda(119+4\lambda)-110)+f(9470-\lambda(263+4\lambda)))(142692+\lambda(\lambda\\ (2097+8\lambda(87+2\lambda))-114740)\text{)) } \end{matrix}$$

From Fig S5, we have:

$$(3(67+4\text{λ})(\text{λ}(1884+\text{λ}(147+4\text{λ}))-2660)((\text{λ-1})\text{λ}(\text{λ}(119+4\text{λ})-110)+f(9470-\lambda$$

$(263+4\lambda)))(142692+\lambda(\lambda(2097+8\lambda(87+2\lambda))-114740)))<0$.

It can be shown that:$\frac{\text{θ}_{i}^{S}-\text{θ}_{A}^{D}}{\triangle\text{s}}<0$,i.e.$\text{θ}_{A}^{D}>\text{θ}_{i}^{S}$. Similarly, we can prove$\text{θ}_{A}^{D}-\text{θ}_{B}^{D}>0\text{、θ}_{A}^{D}-\text{θ}_{i}^{S}>0\text{、θ}_{B}^{D}-\text{θ}_{i}^{S}>0$, i.e. $\text{θ}_{A}^{D}>\text{θ}_{B}^{D}>\text{θ}_{i}^{S}$. The proof is complete.

Appendix S8. Proof of Theorem 4

$$\begin{matrix} \text{ e}_{A}^{D}-\text{e}_{i}^{S}=-(((67+4\lambda)(\lambda(1884+\lambda(147+4\lambda))-2660)(\triangle s(\lambda-1)\lambda(\lambda(119+4\lambda)-110) \\ +t(9470-\lambda(263+4\lambda)))(540716+\lambda(5\lambda(4785+8\lambda(95+2\lambda))-486976)))/4(\lambda-1)(\lambda(95 \\ +4\lambda)-512)(854387017616+\lambda(\lambda(216667089416+\lambda(6230316280+\lambda(16\lambda(2\lambda\text{（}28903 \\ +8\lambda\text{（}391+5\lambda))-5547017)-1895596843)))))-949817488736)) \end{matrix}$$

The image is plotted with the help of Mathematica scientific computing software and is obtained from Fig S6:

$$4(\lambda-1)(\lambda(95+4\lambda)-512)(854387017616+\lambda(\lambda(216667089416+\lambda(6230316$$

$280+\lambda(16\lambda(2\lambda\text{（}28903+8\lambda\text{（}391+5\lambda))-5547017)-1895596843)))))-949817488736))>0$.


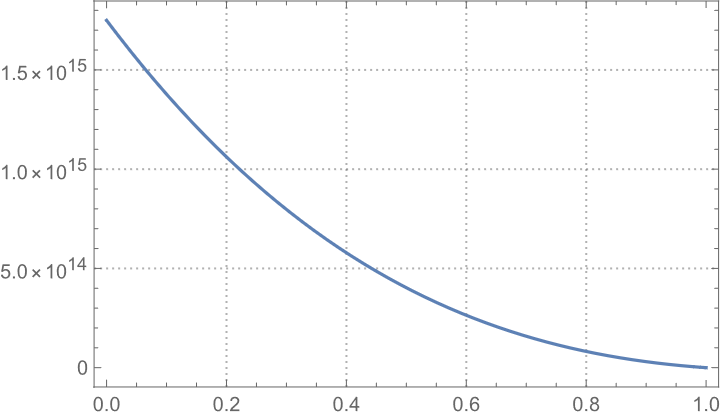


**Fig S6.**$\mathrm{e}_{\mathrm{A}}^{\mathrm{D}}-\mathrm{e}_{\mathrm{i}}^{\mathrm{S}}$positive and negative judgement of the denominator part

we have$\triangle\text{s}>0$and$f>0$when$H-L=\triangle\text{s}$and$\frac{t}{H-L}=f$.

$$-((67+4\lambda)(\lambda(1884+\lambda(147+4\lambda))-2660)(\triangle s(\lambda-1)\lambda(\lambda(119+4\lambda)-110)+t$$

$$\begin{matrix} (9470-\lambda(263+4\lambda)))(540716+\lambda(5\lambda(4785+8\lambda(95+2\lambda)))))/\triangle s=-((67+4\lambda)(\lambda\\ (1884+\lambda(147+4\lambda))-2660)((\lambda-1)\lambda(\lambda(119+4\lambda)-110)+f(9470-\lambda(263+4\lambda))) \\ (540716+\lambda(5\lambda(4785+8\lambda(95+2\lambda))))). \end{matrix}$$

From Fig S7, we have:

$$-((67+4\lambda)(\lambda(1884+\lambda(147+4\lambda))-2660)((\lambda-1)\lambda(\lambda(119+4\lambda)-110)+f(9470-\lambda$$

$$(263+4\lambda)))(540716+\lambda(5\lambda(4785+8\lambda(95+2\lambda)))))>0\text{ }$$

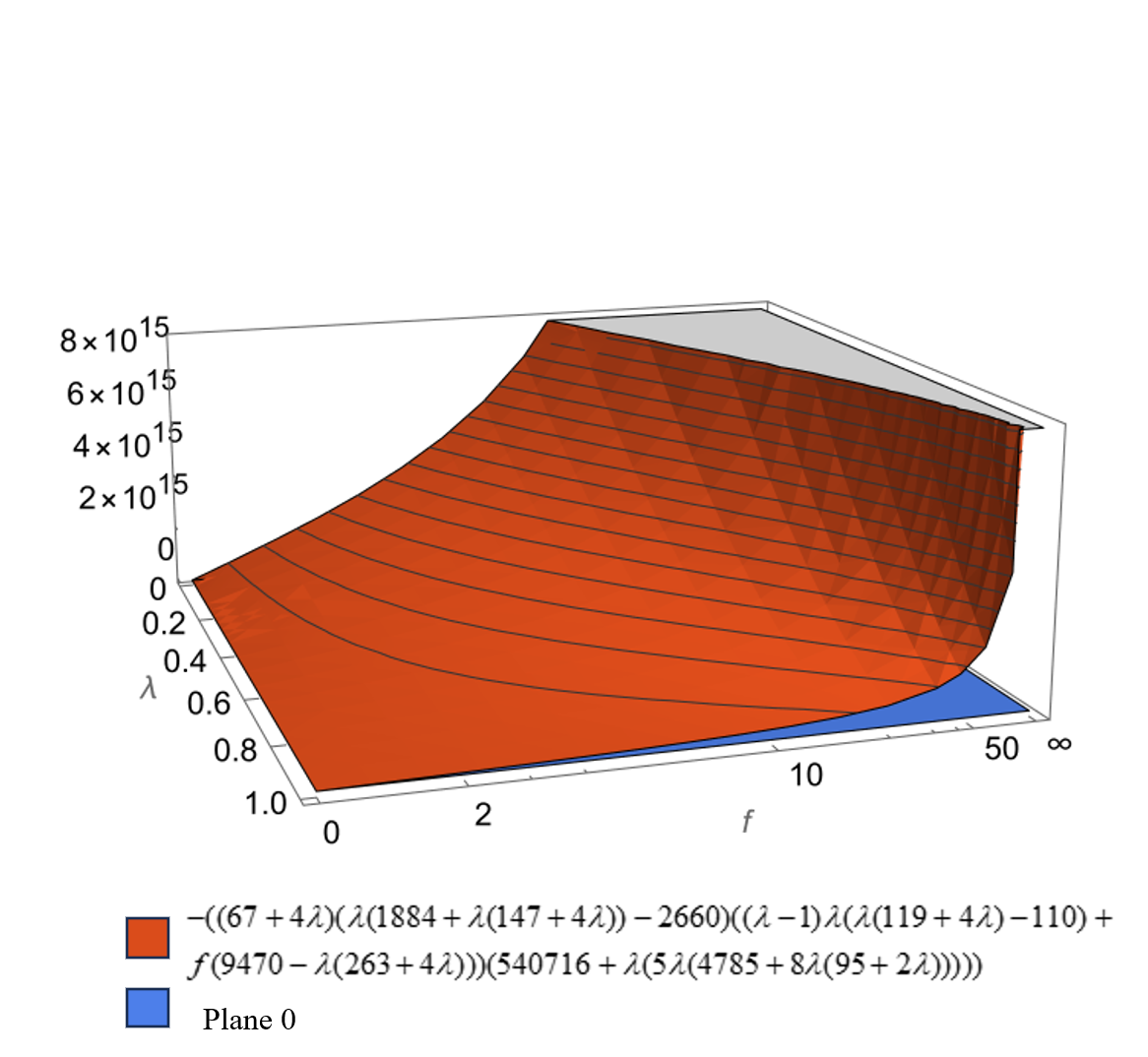


**Fig S7.** Comparison of Plane 5 and Plane 0 Size

This yields$\frac{\text{e}_{A}^{D}-\text{e}_{i}^{S}}{\triangle\text{s}}>0$, i.e.$\text{e}_{A}^{D}>\text{e}_{i}^{S}$. Similarly,$\text{e}_{A}^{D}-\text{e}_{B}^{D}>0\text{、e}_{B}^{D}-\text{e}_{i}^{S}>0$, i.e.,$\text{e}_{A}^{D}>\text{e}_{B}^{D}>\text{e}_{i}^{S}$is obtained by proving. The proof is complete.

Appendix S9. Proof of Theorem 5

$$\begin{matrix} a_{AR}^{D}-a_{BR}^{D}=\frac{\text{Δs}\left(\lambda-1\right)\lambda\left(17955+4\lambda\left(\lambda\left(24\lambda\left(13+2\lambda\right)-721\right)-3559\right)\right)}{192\left(\lambda-1\right)\left(\lambda\left(3+\lambda\right)-5\right)\left(4\lambda\left(5+\lambda\right)-95\right)}+ \\ \frac{t\left(\lambda\left(55743+2\lambda\left(2\lambda\left(2\lambda\left(421+72\lambda\right)-1727\right)-15979\right)\right)-14915\right)}{192\left(\lambda-1\right)\left(\lambda\left(3+\lambda\right)-5\right)\left(4\lambda\left(5+\lambda\right)-95\right)} \end{matrix}$$

From$0<\lambda<1$, we know that $192\left(\lambda-1\right)\left(\lambda\left(3+\lambda\right)-5\right)\left(4\lambda\left(5+\lambda\right)-95\right)<0$. Let$f=\frac{t}{H-L}$, then:

$$\begin{matrix} (\text{Δs}\left(\lambda-1\right)\lambda(17955+4\lambda(\lambda(24\lambda(13+2\lambda)-721-3559))+t(\lambda(55743+2\lambda(2\lambda(2\lambda\\ (421+72\lambda)-1727)-15979))-14915))/\triangle s=(\left(\lambda-1\right)\lambda(17955+4\lambda(\lambda(24\lambda(13+2\lambda)- \\ 721-3559))+f(\lambda(55743+2\lambda(2\lambda(2\lambda(421+72\lambda)-1727)-15979))-14915)) \end{matrix}$$

Solve inequalities with the help of Mathematic scientific computing software:

$$\begin{matrix} (\left(\lambda-1\right)\lambda(17955+4\lambda(\lambda(24\lambda(13+2\lambda)-721-3559))+f(\lambda(55743+2\lambda(2\lambda(2\lambda(421 \\ +72\lambda)-1727)-15979))-14915))<0\text{ }; \end{matrix}$$

$$\begin{matrix} (\left(\lambda-1\right)\lambda(17955+4\lambda(\lambda(24\lambda(13+2\lambda)-721-3559))+f(\lambda(55743+2\lambda(2\lambda(2\lambda(421 \\ +72\lambda)-1727)-15979))-14915))>0\text{ ；} \end{matrix}$$

Seek separately:

$$0<f<\frac{\left(1-\text{λ}\right)\text{λ}\left(17955+4\text{λ}\left(\text{λ}\left(24\text{λ}\left(13+2\text{λ}\right)-721\right)-3559\right)\right)}{\text{λ}\left(55743+2\text{λ}\left(2\text{λ}\left(2\text{λ}\left(421+72\text{λ}\right)-1727\right)-15979\right)\right)-14915}$$

$$f>\frac{\left(1-\text{λ}\right)\text{λ}\left(17955+4\text{λ}\left(\text{λ}\left(24\text{λ}\left(13+2\text{λ}\right)-721\right)-3559\right)\right)}{\text{λ}\left(55743+2\text{λ}\left(2\text{λ}\left(2\text{λ}\left(421+72\text{λ}\right)-1727\right)-15979\right)\right)-14915}>0$$

Let $l1=\frac{\left(1-\text{λ}\right)\text{λ}\left(17955+4\text{λ}\left(\text{λ}\left(24\text{λ}\left(13+2\text{λ}\right)-721\right)-3559\right)\right)}{\text{λ}\left(55743+2\text{λ}\left(2\text{λ}\left(2\text{λ}\left(421+72\text{λ}\right)-1727\right)-15979\right)\right)-14915}$ , we have $a_{AR}^{D}>a_{BR}^{D}$ when$0<f<l1$, $a_{AR}^{D}<a_{BR}^{D}$ when$f>l1>0$.

Similarly, we have$b_{AR}^{D}<b_{BR}^{D}$when$0<f<l2$, $b_{AR}^{D}>b_{BR}^{D}$when $f>l2>0$,$c_{AR}^{D}>c_{BR}^{D}$ when$0<f<l3$, and$c_{AR}^{D}<c_{BR}^{D}$ when$f>l3>0$. Among them:

$$\begin{matrix} l2=\left(\lambda-1\right)\lambda\left(10140+\lambda\left(4\lambda\left(\lambda\left(227+24\lambda\right)-171\right)-9557\right)\right)/(\lambda(\lambda(2\lambda(2163+ \\ 2\lambda(643+70\lambda))-7247)-3361)-980) \end{matrix}$$

$$\begin{matrix} \text{ l}3=\left(1-\text{λ}\right)\text{λ}\left(9855+\text{λ}\left(4\text{λ}\left(\text{λ}\left(157+24\text{λ}\right)-390\right)-7647\right)\right)/(\lambda(18606+\lambda(2\lambda(14\lambda\\ (67+10\lambda)-643)-13165))-4815) \end{matrix}$$

The proof is complete.

Appendix S10. Proof of Theorem 6

$\begin{matrix} f1=((\lambda-1)^{2}\text{（}399774099022596970997248+\lambda(\lambda(804100963412334028991040+\lambda(\lambda(2747 \\ 7368645150004307184+\lambda(3611168493396082390800+\lambda(\lambda(\lambda(3534231669392985939+16 \\ \lambda(17069241885408355+2\lambda(32\lambda(\lambda(4\lambda(2\lambda(1438371+40\lambda(782+5\lambda))-37634281)-3021490 \\ 2121)923529209040)-140183505478279)))-32421564081006385136)-518567421643441 \\ 654724)))-280547319474850298634112))-947338618196438316730112)))/(381108652820 \\ 327310817664+\lambda(\lambda(1633913351813036806996320+\lambda(\lambda(190953474798460250637496+\lambda\\ (12033587461920552407304+\lambda(\lambda(\lambda(1615518011464249315+32\lambda(52463823742425415+\lambda\\ (685713684209791+8\lambda(8\lambda(4\lambda(\lambda(6509637+40\lambda(3643+25\lambda))-42086884)-48122627065) \\ -9359301002913))))-210604404137003695996)-3395201236256200240438)))-9342515 \\ 51637504172299008))-1278755212770931524171136)\text{) } \end{matrix}$ $\begin{matrix} \text{f2}=((\lambda-1)^{2}(134553333394603470409216+\lambda(\lambda(279588542950150119725504+\lambda(\lambda(1048 \\ 8795556160676372368+\lambda(1589006969645840793200+\lambda(\lambda(\lambda(383371187304279153+256 \\ \lambda(449536551200560+\lambda(16381571480613+2\lambda(8636568127+\lambda(32\lambda(1245569+2\lambda(80365 \\ +2\lambda(782+5\lambda)))-3882475959)))))-23485332369560677456)-211714965070023227260)))) \\ -101222244316843952724096))-322584889839277760073984))/(66952490752322609251968 \\ +\lambda(\lambda(307436169930979993332640+\lambda(\lambda(45300927066653784187784+\lambda(1883802420101927 \\ 65816+\lambda(\lambda(1174295928893405916+\lambda(9149328594511691433+32\lambda(17307872516705415+ \\ 8\lambda(137390746433+2\lambda(\lambda(16\lambda(12277843+4\lambda(719093+10\lambda(1408+9\lambda)))-63192146807)- \\ 2025290656648))))-856485189880706331610))))-187364293210934383800064))-231469 \\ 924559800290484864)) \end{matrix}$ Let $f=\frac{t}{H-L}$, then:

$$\begin{matrix} \frac{\partial e_{A}^{D}}{\partial\text{λ}}=(t(381108652820327310817664+\lambda(\lambda(1633913351813036806996320+\lambda(\lambda\\ (190953474798460250637496+\lambda(12033587461920552407304+\lambda(\lambda(\lambda(161555180 \\ 11464249315+32\lambda(52463823742425415+\lambda(685713684209791+8\lambda(8\lambda(4\lambda(\lambda(650 \\ 9637+40\lambda(3643+25\lambda))-42086884)-48122627065)-9359301002913))))-21060 \\ 4404137003695996)-3395201236256200240438)))-934251551637504172299008)) \\ -1278755212770931524171136))-(\lambda-1)^{2}(399774099022596970997248+\lambda(\lambda\text{（}8041 \\ 00963412334028991040+\lambda\text{（}\lambda(27477368645150004307184+\lambda(3611168493396082390800 \\ +\lambda(\lambda(\lambda(3534231669392985939+16\lambda(17069241885408355+2\lambda(32\lambda(\lambda(4\lambda(2\lambda(1438371+ \\ 40\lambda(782+5\lambda))-37634281)-30214902121)-923529209040)-140183505478279)))- \\ 32421564081006385136)-518567421643441654724)))-280547319474850298634112))- \\ 947338618196438316730112))\triangle s)/((\lambda-1)^{2}(854387017616+\lambda(\lambda(216667089416+\lambda(623 \\ 0316280+\lambda(+16\lambda(2\lambda(28903+8\lambda(391+5\lambda))-5547017)-1895596843)))-949817488736))^{2}) \end{matrix}$$

It is easy to know that the denominator is positive and:

$$\begin{matrix} (t(381108652820327310817664+\lambda(\lambda(1633913351813036806996320+\lambda(\lambda(19095347 \\ 4798460250637496+\lambda(12033587461920552407304+\lambda(\lambda(\lambda(16155518011464249315+ \\ 32\lambda(52463823742425415+\lambda(685713684209791+8\lambda(8\lambda(4\lambda(\lambda(6509637+40\lambda(3643+ \\ 25\lambda))-42086884)-48122627065)-9359301002913))))-210604404137003695996) \\ -3395201236256200240438)))-934251551637504172299008))-12787552127709315 \\ 24171136))-(\lambda-1)^{2}(399774099022596970997248+\lambda(\lambda\text{（}804100963412334028991040 \\ +\lambda\text{（}\lambda(27477368645150004307184+\lambda(3611168493396082390800+\lambda(\lambda(\lambda(353423166 \\ 9392985939+16\lambda(17069241885408355+2\lambda(32\lambda(\lambda(4\lambda(2\lambda(1438371+40\lambda(782+5\lambda)) \\ -37634281)-30214902121)-923529209040)-140183505478279)))-3242156408100 \\ 6385136)-518567421643441654724)))-280547319474850298634112))-9473386181 \\ 96438316730112))\triangle s)/\triangle s=f(381108652820327310817664+\lambda(\lambda(16339133518130368 \\ 06996320+\lambda(\lambda(190953474798460250637496+\lambda(12033587461920552407304+\lambda(\lambda(\lambda\\ (16155518011464249315+32\lambda(52463823742425415+\lambda(685713684209791+8\lambda(8\lambda(4\lambda\\ (\lambda(6509637+40\lambda(3643+25\lambda))-42086884)-48122627065)-9359301002913))))- \\ 210604404137003695996)-3395201236256200240438)))-934251551637504172299008)) \\ -1278755212770931524171136))-(\lambda-1)^{2}(399774099022596970997248+\lambda(\lambda\text{（}804100963 \\ 412334028991040+\lambda\text{（}\lambda(27477368645150004307184+\lambda(3611168493396082390800+\lambda(\lambda\\ (\lambda(3534231669392985939+16\lambda(17069241885408355+2\lambda(32\lambda(\lambda(4\lambda(2\lambda(1438371+40\lambda(782 \\ +5\lambda))-37634281)-30214902121)-923529209040)-140183505478279)))-324215640810 \\ 06385136)-518567421643441654724)))-280547319474850298634112))-9473386181964 \\ 38316730112))=C \end{matrix}$$

Solve inequalities with the help of Mathematic scientific computing software:

$C>0$;

$C<0$;

Find$f>f1>0$ and $0<f<f1$ respectively. That is to say, when$f>f1>0$, it satisfies $\frac{\partial e_{A}^{D}}{\partial\text{λ}}>0$; when $0<f<f1$, it satisfies $\frac{\partial e_{A}^{D}}{\partial\text{λ}}<0$. Similarly, when$f>f2>0$ ,$\frac{\partial e_{B}^{D}}{\partial\text{λ}}>0$ is satisfied; when$0<f<f2$ ,$\frac{\partial e_{B}^{D}}{\partial\text{λ}}<0$is satisfied.

Plotting the image of $f1\text{、}f2$ reveals$f1<f2$. The proof is complete.

Proofs of Equations

Appendix S11. Proof of Equation (1) to Equation (22)

For Equation (1), using the Hotelling linear urban model, consumers compare the utility difference between the two options: “staying on the original platform” and “switching platforms” in the second period. Assume that the consumer's distance to platform $E_{A}$ is $x$, and to platform $E_{B}$ is $1-x$. As assumed in the model description, only a subset of consumers who perceive low product quality in the first period may switch channels for purchase. Under the self-operated model, the platform offers a price discount to new consumers, with a discount coefficient of . The utility functions for consumers repeating purchases or switching platforms in the second period can be specified as follows:

Utility from repeat purchasing product$A$:$L-b_{AR}^{S}-tx$

Utility from switching to purchase product$B$:$\bar{v}-c_{BR}^{S}-t\left(1-x\right)+\beta^{k}c_{BR}^{S}$

Utility from repeat purchasing product$B$:$L-b_{BR}^{S}-t\left(1-x\right)$

Utility from switching to purchase product$A$:$\bar{v}-c_{AR}^{S}-tx+\beta^{k}c_{AR}^{S}$

Following the approach of Ma et al. [1], we derive the indifference point between consumers repeating and switching purchases in the second period by comparing consumer utilities, as shown in Equations (1) and (2):

| $L-b_{AR}^{S}-tx_{0}=\bar{v}-c_{BR}^{S}-t\left(1-x_{0}\right)+\beta^{k}c_{BR}^{S}$ |
| --- |
| $L-b_{BR}^{S}-t\left(1-x_{1}\right)=\bar{v}-c_{AR}^{S}-tx_{1}+\beta^{k}c_{AR}^{S}$ |

The solution yields two utility indifference points: , representing the threshold where consumers are indifferent between continuing to purchase product$A$on platform $E_{A}$ and switching to purchase product$B$on platform $E_{B}$; and $x_{1}$, representing the threshold where consumers are indifferent between continuing to purchase product$B$on platform $E_{B}$ and switching to purchase product$A$on platform $E_{A}$, as specified in Equations (3) and (4).

$$x_{0}=\frac{t-b_{AR}^{S}-\lambda(H-L)-c_{BR}^{S}\left(\beta^{S}-1\right)}{2t}$$

$$x_{1}=\frac{t+b_{BR}^{S}+\lambda(H-L)+c_{AR}^{S}\left(\beta^{S}-1\right)}{2t}$$

Strategic consumers make decisions by maximizing their total utility across both periods [1,2]. Therefore, the utility indifference point $y$ in the first period must satisfy the condition where the cumulative utility of "purchasing on platform $E_{A}$ in Period 1 and switching to platform $E_{B}$ in Period 2" equals that of "purchasing on platform $E_{B}$ in Period 1 and switching to platform $E_{A}$ in Period 2."The product$A$first-period market demand is$d_{A}=\text{y}$, and the second-period market demand includes both repeat and switching consumers, i.e. $D_{A}=\lambda\text{y}+\left(1-\lambda\right)x_{0}+\left(1-\lambda\right)\left(x_{1}-y\right)$; similarly, the product$B$first-period market demand is$d_{B}=\left(1-\text{y}\right)$, and the second-period market demand is$D_{B}=\lambda(1-\text{y)}+(1-\lambda)(1-x_{1})+(1-\lambda)(y-x_{0})$. The above derivation leads to Equation (5):

$$\bar{v}-c_{BR}^{S}-t\left(1-x_{0}\right)+\beta^{S}c_{BR}^{S}+\bar{v}-a_{AR}^{S}-td_{A}=(\bar{v}-c_{AR}^{S}-tx_{1}+\beta^{S}c_{AR}^{S})+(\bar{v}-a_{BR}^{S}-td_{B})$$

Based on the Nash equilibrium solution, we derive the utility indifference point for purchasing the two products in the first period, as formalized in Equation (6):

$$\text{y}=\frac{t\left(\lambda-2\right)-a_{AR}^{S}\left(\lambda-1\right)+a_{BR}^{S}\left(\lambda-1\right)}{2t\left(\lambda-2\right)}$$

The profit of the self-operated platform is calculated as (selling price - wholesale price - price discount) × market demand. Since only consumers who switch platforms in the second period are eligible for the price discount, we derive the platform's second-period profit and total two-period profit, as specified in Equations (7)-(10):

$$\pi(E_{AR}^{S})=\left(b_{AR}^{S}-\omega_{A}^{S}\right)\left(\lambda y+\left(1-\lambda\right)x_{0}\right)+\left(1-\lambda\right)\left(x_{1}-y\right)\left(c_{AR}^{S}-\omega_{A}^{S}-\beta^{S}c_{AR}^{S}\right)$$

$$\Pi(E_{AR}^{S})=\left(a_{AR}^{S}-\omega_{A}^{S}\right)d_{A}+\left(b_{AR}^{S}-\omega_{A}^{S}\right)\left(\lambda y+\left(1-\lambda\right)x_{0}\right)+\left(1-\lambda\right)\left(x_{1}-y\right)\left(c_{AR}^{S}-\omega_{A}^{S}-\beta^{S}c_{AR}^{S}\right)$$

$$\Pi(E_{BR}^{S})=d_{B}(a_{BR}^{S}-\omega_{B}^{S})+(b_{BR}^{S}-\omega_{B}^{S})(\lambda(1-\text{y)+(1-}\lambda)(1-x_{1}))+(1-\lambda)(y-x_{0})(c_{BR}^{S}-\omega_{B}^{S}-\beta^{S}c_{BR}^{S})$$

The manufacturer$M_{j}$generates profit by charging the platform wholesale prices, from which we derive the manufacturer's second-period profit and total two-period profit, as formalized in Equations (11)-(14):

$$\pi(M_{AR}^{S})=\omega_{A}^{S}D_{A}$$

$$\pi(M_{BR}^{S})=w_{B}^{S}D_{B}$$

$$\Pi(M_{AR}^{S})=\omega_{A}^{S}d_{A}+\omega_{A}^{S}D_{A}$$

$$\Pi(M_{BR}^{S})=\omega_{B}^{S}d_{B}+\omega_{B}^{S}D_{B}$$

Using backward induction, we obtain the Nash equilibrium solution as specified in Equations (15)-(22), with the detailed derivation process provided in Appendix S1.

Appendix S12. Proof of Equation (23) to Equation (41)

Similar to Appendix S1, both platforms make decisions simultaneously, with the key difference lying in their operational models. In the commission-based model, the platform gives consumers a discount amount $e_{i}^{k},i=(A,B),k=(S,D)$to attract consumers. The utility functions for consumers repeating purchases or switching platforms in the second period can be specified as follows:

Utility from repeat purchasing product$A$:$L-b_{AC}^{S}-tx_{0}$

Utility from switching to purchase product$B$:$\bar{v}-c_{BC}^{S}-t\left(1-x_{0}\right)+e_{i}^{k}$

Utility from repeat purchasing product$B$:$L-b_{BC}^{S}-t\left(1-x_{1}\right)$

Utility from switching to purchase product$A$:

By comparing consumer utilities, we derive the utility indifference points for consumers repeating purchases or switching platforms in the second period, as formalized in Equations (23) and (24):

| $L-b_{AC}^{S}-tx_{0}=\bar{v}-c_{BC}^{S}-t\left(1-x_{0}\right)+e_{i}^{k}$ |
| --- |
|  |

The solution yields two utility indifference points: $x_{0}$, representing the threshold where consumers are indifferent between continuing to purchase product$A$on platform $E_{A}$ and switching to purchase product$B$on platform $E_{B}$; and $x_{1}$, representing the threshold where consumers are indifferent between continuing to purchase product$B$on platform $E_{B}$ and switching to purchase product$A$on platform $E_{A}$, as specified in Equations (25) and (26).

$$x_{0}=\frac{t-(H-L)\lambda-b_{AC}^{S}+c_{BC}^{S}-e_{B}^{S}}{2t}$$

$$x_{1}=\frac{t+(H-L)\lambda+b_{BC}^{S}-c_{AC}^{S}+e_{A}^{S}}{2t}$$

Following the approach in Appendix S11, the utility indifference point$y$in the first period must satisfy the condition where the cumulative utility of "purchasing on platform $E_{A}$ in Period 1 and switching to platform $E_{B}$ in Period 2" equals that of "purchasing on platform $E_{B}$ in Period 1 and switching to platform $E_{A}$ in Period 2."As demonstrated in Equations (27) and (28):

|  |  |
| --- | --- |

$$\text{y}=\frac{6t\left(\lambda-2\right)-4\left(\lambda-1\right)a_{AC}^{S}+4\left(\lambda-1\right)a_{BC}^{S}-3\left(\lambda-1\right)\theta_{A}^{S}+3\left(\lambda-1\right)\theta_{B}^{S}}{12t\left(\lambda-2\right)}$$

The profit of the commission-based platform is calculated as (commission - price discount) × market demand, where only consumers who switch platforms in the second period qualify for the price discount. This yields the platform's second-period profit and total two-period profit, as specified in Equations (29)-(32):

$$\pi(E_{AC}^{S})=\theta_{A}^{S}\left(\lambda y+\left(1-\lambda\right)x_{0}\right)+\left(\theta_{A}^{S}-e_{A}^{S}\right)\left(1-\lambda\right)\left(x_{1}-y\right)$$

$$\pi(E_{BC}^{S})=\theta_{B}^{S}\left(\lambda\left(1-\text{y}\right)+\left(1-\lambda\right)\left(1-x_{1}\right)\right)+\left(\theta_{B}^{S}-e_{B}^{S}\right)\left(1-\lambda\right)\left(y-x_{0}\right)$$

$$\Pi(E_{AC}^{S})=\theta_{A}^{S}y+\pi(E_{AC}^{S})$$

$$\Pi(E_{BC}^{S})=\theta_{B}^{S}\left(1-y\right)+\pi(E_{BC}^{S})$$

Under the commission-based model, the manufacturer's profit equals sales revenue minus commissions paid to the platform. This yields the manufacturer's second-period profit and total two-period profit, as formalized in Equations (33)-(36):

$$\pi(M_{AC}^{S})=\left(b_{AC}^{S}-\theta_{A}^{S}\right)\left(\lambda y+\left(1-\lambda\right)x_{0}\right)+\left(c_{AC}^{S}-\theta_{A}^{S}\right)\left(1-\lambda\right)\left(x_{1}-y\right)$$

$$\pi(M_{BC}^{S})=\left(b_{BC}^{S}-\theta_{B}^{S}\right)\left(\lambda\left(1-y\right)+\left(1-\lambda\right)\left(1-x_{1}\right)\right)+\left(c_{BC}^{S}-\theta_{B}^{S}\right)\left(1-\lambda\right)\left(y-x_{0}\right)$$

$$\Pi(M_{AC}^{S})=\left(a_{AC}^{S}-\theta_{A}^{S}\right)y+\pi(M_{AC}^{S})$$

$$\Pi(M_{BC}^{S})=\left(a_{BC}^{S}-\theta_{B}^{S}\right)\left(1-\text{y}\right)+\pi(M_{BC}^{S})$$

Through backward induction, we obtain the Nash equilibrium solution as specified in Equations (37)-(41), with the complete derivation process detailed in Appendix S2.

Appendix S13. Proof of Equation (42) to Equation (60)

The proofs for Equations (42)-(60) follow a similar approach to Appendix S11, with the only difference being the platforms' decision sequence, which alters the backward induction process for equilibrium derivation: the leader platform makes decisions first, followed by the follower platform. The detailed proofs are provided in Appendix S3.

Appendix S14. Proof of Equation (61) to Equation (71)

The proofs for Equations (61)-(71) follow a similar approach to Appendix S12, with the only difference being the platforms' decision sequence, which alters the backward induction process for equilibrium derivation: the leader platform makes decisions first, followed by the follower platform. The detailed proofs are provided in Appendix S4.

Equilibrium solutions

Appendix S15. Summary of equilibrium solutions

**Table 3.** The game equilibrium solution of self-operated platforms

| 变量 | S-R | D-R |
| --- | --- | --- |
| $a_{A,g}^{k}$ | $\frac{2}{3}\left((H-L)\lambda+\frac{t\left(90-\lambda\left(67+7\lambda\right)\right)}{\left(\lambda-1\right)\left(2\lambda-13\right)}\right)$ |  |
| $a_{B,g}^{k}$ | $\frac{2}{3}\left((H-L)\lambda+\frac{t\left(90-\lambda\left(67+7\lambda\right)\right)}{\left(\lambda-1\right)\left(2\lambda-13\right)}\right)$ | $\frac{11147t-\frac{11558t}{\lambda-1}+71\lambda\left(59H-59L+2t\right)+\frac{142\left(17t+\lambda\left(L-H+4t\right)\right)}{\lambda\left(3+\lambda\right)-5}-\frac{24\left(497\lambda\left(L-H\right)+4t\left(18446+4589\lambda\right)\right)}{4\lambda\left(5+\lambda\right)-95}}{6816}$ |
| $b_{A,g}^{k}$ | $\frac{1}{3}\left[\frac{t\left(128-\lambda\left(61+24\lambda\right)\right)}{\left(\lambda-1\right)\left(2\lambda-13\right)}-(H-L)\lambda\right]$ | $\begin{matrix} \frac{\left(\text{λ}\left(\text{H-L}\right)\left(\text{1-λ}\right)\left(\text{11145+λ}\left(\text{4λ}\left(\text{3λ}\left(\text{67+8λ}\right)\text{-292}\right)\text{-9379}\right)\right)\right)}{48\left(\lambda-1\right)\left(\lambda\left(3+\lambda\right)-5\right)\left(4\lambda\left(5+\lambda\right)-95\right)}+ \\ \frac{t\left(\lambda\left(109188+\lambda\left(3293+2\lambda\left(2\lambda\left(18\lambda-653\right)-9485\right)\right)\right)-98215\right)}{48\left(\lambda-1\right)\left(\lambda\left(3+\lambda\right)-5\right)\left(4\lambda\left(5+\lambda\right)-95\right)} \end{matrix}$ |
| $b_{B,g}^{k}$ | $\frac{1}{3}\left[\frac{t\left(128-\lambda\left(61+24\lambda\right)\right)}{\left(\lambda-1\right)\left(2\lambda-13\right)}-(H-L)\lambda\right]$ |  |
| $c_{A,g}^{k}$ |  |  |
| $c_{B,g}^{k}$ |  |  |
| $\omega_{A}^{k}$ | $\frac{t\left(34-\lambda\left(19+8\lambda\right)\right)}{\left(\lambda-1\right)\left(2\lambda-13\right)}$ | $\frac{\left(\lambda\left(H-L\right)\left(1-\lambda\right)\left(3+2\lambda\right)\right)+t\left(899+\lambda\left(4\left(\lambda-41\right)\lambda-545\right)\right)}{3\left(\lambda-1\right)\left(4\lambda\left(5+\lambda\right)-95\right)}$ |
| $\omega_{B}^{k}$ | $\frac{t\left(34-\lambda\left(19+8\lambda\right)\right)}{\left(\lambda-1\right)\left(2\lambda-13\right)}$ | $\frac{1021t+\lambda\left(H-L\right)\left(\lambda-1\right)\left(3+2\lambda\right)-t\lambda\left(607+4\lambda\left(55+\lambda\right)\right)}{3\left(\lambda-1\right)\left(4\lambda\left(5+\lambda\right)-95\right)}$ |

**Table 4.** The game equilibrium solution of commission-based platforms

| 变量 | S-C | D-C |
| --- | --- | --- |
| $a_{A,g}^{k}$ | $\frac{2\lambda\left(H-L\right)\left(1-\lambda\right)\left(292+\lambda\left(143+4\lambda\right)\right)+t\left(17416+\lambda\left(+\lambda\left(629+44\lambda\right)-14840\right)\right)}{6\left(\lambda-1\right)\left(\lambda\left(95+4\lambda\right)-512\right)}$ | $\begin{matrix} (((H-L)(1-\lambda)\lambda(\lambda(531431374384+\lambda(278396126768+\lambda(\lambda(4\lambda(4\lambda(418961 \\ +4\lambda(5041+56\lambda))-38673485)-6733603987)-47198747420)))-1022023303312)) \\ +t(-33150803072688+\lambda(56507026168288+\lambda(\lambda(2255244859288+\lambda\text{（}255864960371 \\ +\lambda\text{（}32\lambda\text{（}\lambda\text{（}3359+40\lambda\text{（}757+11\lambda))-33429575)-12444370925)))-27188422935136)) \\ -33150803072688))/(6(\lambda-1)(854387017616+\lambda(\lambda(216667089416+\lambda(6230316280+ \\ \lambda(16\lambda(2\lambda(28903+8\lambda(391+5\lambda))-5547017)-1895596843)))-949817488736))) \end{matrix}$ |
| $a_{B,g}^{k}$ | $\frac{2\lambda\left(H-L\right)\left(1-\lambda\right)\left(292+\lambda\left(143+4\lambda\right)\right)+t\left(17416+\lambda\left(+\lambda\left(629+44\lambda\right)-14840\right)\right)}{6\left(\lambda-1\right)\left(\lambda\left(95+4\lambda\right)-512\right)}$ | $\begin{matrix} (((H-L)(1-\lambda)\lambda(\lambda(533060839504+\lambda(270438966704+\lambda(\lambda(4\lambda(4\lambda(509303 \\ +4\lambda(5311+56\lambda))-27078659)-6057290545)-43930520324)))-1019906049712)) \\ +t(\lambda(56839435942528+\lambda(\lambda(2218476834004+\lambda(255051057845+\lambda(32\lambda(\lambda(3899 \\ +40\lambda(757+11\lambda))-33364964)-12404896709)))-27511199806624))-3296852678 \\ 5488))/(6(\lambda-1)(854387017616+\lambda(\lambda(216667089416+\lambda(6230316280+\lambda(16\lambda(2\lambda\\ (28903+8\lambda(391+5\lambda))-5547017)-1895596843)))-949817488736))) \end{matrix}$ |
| $b_{A,g}^{k}$ | $\frac{\lambda\left(H-L\right)\left(\lambda-1\right)\left(\lambda\left(785+28\lambda\right)-1574\right)+5t\left(\lambda\left(2207+100\lambda\right)-3842\right)}{12\left(1-\lambda\right)\left(\lambda\left(95+4\lambda\right)-512\right)}$ | $\begin{matrix} (\left(H-L\right)\left(\lambda-1\right)\lambda(638781615208+\lambda(\lambda\text{ }(318882388426+\lambda(\lambda(16\lambda(2\lambda(212267+ \\ 8\lambda(1160+13\lambda))-4238899)-3383206091)-5115392899))-891241448548))+t(955 \\ 1765639720+\lambda(\lambda(5269008760574+\lambda(86785414771+8\lambda(8\lambda(4\lambda(93785+\lambda(11365+ \\ 152\lambda))-39407831)-6698702351)))-14226916556060)))/(3\left(1-\lambda\right)(854387017616+ \\ \lambda(\lambda\text{ }(216667089416+\lambda(6230316280+\lambda(16\lambda(2\lambda(28903+8\lambda(391+5\lambda))-5547017- \\ 1895596843)))-949817488736))) \end{matrix}$ |
| $b_{B,g}^{k}$ | $\frac{\lambda\left(H-L\right)\left(\lambda-1\right)\left(\lambda\left(785+28\lambda\right)-1574\right)+5t\left(\lambda\left(2207+100\lambda\right)-3842\right)}{12\left(1-\lambda\right)\left(\lambda\left(95+4\lambda\right)-512\right)}$ | $\begin{matrix} (((H-L)(1-\lambda)\lambda(645486251608+\lambda(\lambda(340129529470+\lambda(\lambda(8\lambda(8\lambda(14\lambda(151+2\lambda) \\ -1097)-26757551)-4879041347)-9344291641))-913194084268)))+t(\lambda(129453983 \\ 85620+\lambda(\lambda(10812994871-8\lambda(\lambda(4\lambda(982885+4\lambda(24425+316\lambda))-315356287)-7312 \\ 402544))-4752960387626))-8974557396920))/(3(\lambda-1)(854387017616+\lambda(\lambda(21666 \\ 7089416+\lambda(6230316280+\lambda(16\lambda(2\lambda(28903+8\lambda(391+5\lambda))-5547017)-1895596843))) \\ -949817488736))) \end{matrix}$ |
| $c_{A,g}^{k}$ | $\frac{\left(\lambda\left(H-L\right)\left(1-\lambda\right)\left(127+4\lambda\right)\left(2+5\lambda\right)\right)+t\left(20750-\lambda\left(14309+652\lambda\right)\right)}{12\left(\lambda-1\right)\left(\lambda\left(95+4\lambda\right)-512\right)}$ |  |
| $c_{B,g}^{k}$ | $\frac{\left(\lambda\left(H-L\right)\left(1-\lambda\right)\left(127+4\lambda\right)\left(2+5\lambda\right)\right)+t\left(20750-\lambda\left(14309+652\lambda\right)\right)}{12\left(\lambda-1\right)\left(\lambda\left(95+4\lambda\right)-512\right)}$ | $\begin{matrix} (((H-L)(1-\lambda)\lambda(\lambda(\lambda(213761409572+\lambda(\lambda(32\lambda(\lambda(7537+40\lambda(80+\lambda)) \\ -5025862)-4193432095)-19089212774))-140883448808)-118984151968)) \\ +t(\lambda(15321222294328+\lambda(\lambda(12072655186+\lambda(75222109847-32\lambda(\lambda(1239545 \\ +8\lambda(15752+205\lambda))-102151112)))-6148175618044))-9777424528000))/(3(\lambda-1) \\ (854387017616+\lambda(\lambda(216667089416+\lambda(6230316280+\lambda(16\lambda(2\lambda(28903+8\lambda\\ (391+5\lambda))-5547017)-1895596843)))-949817488736))) \end{matrix}$ |
| $\theta_{A}^{k}$ | $\frac{\left(\lambda\left(H-L\right)\left(1-\lambda\right)\left(\lambda\left(119+4\lambda\right)-110\right)\right)+t\left(2818-\lambda\left(2017+92\lambda\right)\right)}{2\left(\lambda-1\right)\left(\lambda\left(95+4\lambda\right)-512\right)}$ | $\begin{matrix} (4(H-L)(1-\lambda)\lambda(\lambda(119+4\lambda)-110)(\lambda(169584228+\lambda(4\lambda(\lambda(7173+8\lambda(123+2\lambda)) \\ -411802)-14744913))-189964676)-4t(764195571128+\lambda(\lambda(466526148878+\lambda\\ (6291464287+\lambda(4\lambda(4\lambda(139427+4\lambda(4183+56\lambda))-57485015)-4922881933))) \\ -1193529117260)))/((\lambda-1)(854387017616+\lambda(\lambda(216667089416+\lambda(6230316280+ \\ \lambda(16\lambda(2\lambda(28903+8\lambda(391+5\lambda))-5547017)-1895596843)))-949817488736))) \end{matrix}$ |
| $\theta_{B}^{k}$ | $\frac{\left(\lambda\left(H-L\right)\left(1-\lambda\right)\left(\lambda\left(119+4\lambda\right)-110\right)\right)+t\left(2818-\lambda\left(2017+92\lambda\right)\right)}{2\left(\lambda-1\right)\left(\lambda\left(95+4\lambda\right)-512\right)}$ | $\begin{matrix} (2(H-L)(1-\lambda)\lambda(\lambda(95+4\lambda)-512)(\lambda(119+4\lambda)-110)(784516+\lambda(\lambda(8\lambda(47+2\lambda) \\ -28479)-575396))+2t(\lambda(2097587265080+\lambda(\lambda(3618760874+\lambda(10676407123-16\lambda\\ (2\lambda(178303+232\lambda(77+\lambda))-28772369)))-854674442468))-1322486447456))/ \\ ((\lambda-1)(854387017616+\lambda(\lambda(216667089416+\lambda(6230316280+\lambda(16\lambda(2\lambda(28903+8\lambda\\ (391+5\lambda))-5547017)-1895596843)))-949817488736))) \end{matrix}$ |
| $e_{A}^{k}$ | $\frac{3t\left(598-7\lambda\left(87+4\lambda\right)\right)-3\lambda\left(H-L\right)\left(\lambda-1\right)\left(\lambda\left(103+4\lambda\right)-378\right)}{4\left(\lambda-1\right)\left(\lambda\left(95+4\lambda\right)-512\right)}$ | $\begin{matrix} (((H-L)(1-\lambda)\lambda(467907506528+\lambda(\lambda(147827416844+\lambda(1785266974 \\ +\lambda(8\lambda(8\lambda(35429+2\lambda(869+10\lambda))-4614529)-1306174489)))-554396660216)) \\ +t(1194023520448+\lambda(\lambda(881839729108+\lambda(19611283226+\lambda(8\lambda(16\lambda(25487+ \\ \lambda(3643+50\lambda))-52182263)-8437427009)))-2075352184552)))/((\lambda-1) \\ (854387017616+\lambda(\lambda(216667089416+\lambda(6230316280+\lambda(16\lambda(2\lambda(28903+ \\ 8\lambda(391+5\lambda))-5547017)-1895596843))))-949817488736))) \end{matrix}$ |
| $e_{B}^{k}$ | $\frac{3t\left(598-7\lambda\left(87+4\lambda\right)\right)-3\lambda\left(H-L\right)\left(\lambda-1\right)\left(\lambda\left(103+4\lambda\right)-378\right)}{4\left(\lambda-1\right)\left(\lambda\left(95+4\lambda\right)-512\right)}$ | $\begin{matrix} (3(H-L)(1-\lambda)\lambda(157485226976+\lambda(\lambda(52436212756+\lambda(102349714 \\ +\lambda(32\lambda(\lambda(8\lambda(72+\lambda)-1907)-942314)-620917491)))-188781479112)) \\ +3t(\lambda(486491942104+\lambda(\lambda(5625810602+\lambda(3396382379-32\lambda(\lambda(62293 \\ +8\lambda(704+9\lambda))-4312788)))-229739973900))-267489017216))/((\lambda-1) \\ (854387017616+\lambda(\lambda(216667089416+\lambda(6230316280+\lambda(16\lambda(2\lambda(28903 \\ +8\lambda(391+5\lambda))-5547017)-1895596843)))-949817488736))) \end{matrix}$ |

Numerical simulation

Appendix S16. Platform operation mode selection

**Table 5.** Platform profits when consumers have a low probability of perceiving high quality

|  | $\Delta s=0.1$ | $\Delta s=5$ | $\Delta s=10$ |
| --- | --- | --- | --- |
| $\Pi(E_{i,C}^{S})$ | $\frac{47707654577371889}{18177555715200000}$ | $\frac{19175194352729}{7271022286080}$ | $\frac{98994338441}{37097052480}$ |
| $\Pi(E_{i,R}^{S})$ | $\frac{10768081}{8100000}$ | $\frac{4741}{3240}$ | $\frac{668}{405}$ |
| $\Pi(E_{AC}^{D})$ | $\frac{71284530657725321337574141}{25703256808346666571000000}$ | $\frac{28658133233837506112101}{10281302723338666628400}$ | $\frac{7248623784695970202021}{2570325680834666657100}$ |
| $\Pi(E_{BC}^{D})$ | $\frac{137672358675306903127935231802031095043447861}{40781321639248103004101463484410425805000000}$ | $\frac{55388135706757690205077164846627001589021}{16312528655699241201640585393764170322000}$ | $\frac{14003572475407496863248041888420045469341}{4078132163924810300410146348441042580500}$ |
| $\Pi(E_{AR}^{D})$ | $\frac{7530382029145853}{3908146233600000}$ | $\frac{3274992684197}{1563258493440}$ | $\frac{907684245389}{390814623360}$ |
| $\Pi(E_{BR}^{D})$ | $\frac{5374417971181724131}{3142149571814400000}$ | $\frac{460520997353087}{251371965745152}$ | $\frac{623449661103067}{314214957181440}$ |

**Note:** $\Pi(E_{BC}^{D})>\Pi(E_{AC}^{D})>\Pi(E_{i,C}^{S})>\Pi(E_{AR}^{D})>\Pi(E_{BR}^{D})>\Pi(E_{i,R}^{S})$

**Table 6.** Platform profits when consumers perceive a moderate probability of high quality

|  | $\Delta s=0.1$ | $\Delta s=5$ | $\Delta s=10$ |
| --- | --- | --- | --- |
| $\Pi(E_{i,C}^{S})$ | $\frac{197184213}{54318080}$ | $\frac{10467945}{2715904}$ | $\frac{2946825}{678976}$ |
| $\Pi(E_{i,R}^{S})$ | $\frac{12481}{7200}$ | $\frac{21}{8}$ | $\frac{38}{9}$ |
| $\Pi(E_{AC}^{D})$ | $\frac{6595451523327}{1694581102400}$ | $\frac{69810520095}{16945811024}$ | $\frac{19525497375}{4236452756}$ |
| $\Pi(E_{BC}^{D})$ | $\frac{8772975593609213262053}{1794753195381999553600}$ | $\frac{92046603044408339205}{17947531953819995536}$ | $\frac{25266854015492685125}{4486882988454998884}$ |
| $\Pi(E_{AR}^{D})$ | $\frac{330606581}{125798400}$ | $\frac{172863}{46592}$ | $\frac{251267}{44928}$ |
| $\Pi(E_{BR}^{D})$ | $\frac{103452823969}{45790617600}$ | $\frac{153212489}{50878464}$ | $\frac{9739081}{2336256}$ |

**Note:** If$\lambda=0.5,\Delta s=10$, then $\Pi(E_{BC}^{D})>\Pi(E_{AR}^{D})>\Pi(E_{AC}^{D})>\Pi(E_{i,C}^{S})>\Pi(E_{i,R}^{S})>\Pi(E_{BR}^{D})$ ; If$\lambda=0.5,\Delta s=0.1$or$\lambda=0.5,\Delta s=5$, then $\Pi(E_{BC}^{D})>\Pi(E_{AC}^{D})>\Pi(E_{i,C}^{S})>\Pi(E_{AR}^{D})>\Pi(E_{BR}^{D})>\Pi(E_{i,R}^{S})$

**Table 7.** Platform profits when consumers have a high probability of perceiving high quality

|  | $\Delta s=0.1$ | $\Delta s=5$ | $\Delta s=10$ |
| --- | --- | --- | --- |
| $\Pi(E_{i,C}^{S})$ | $\frac{15604534722191649}{1433192220800000}$ | $\frac{6501766663689}{573276888320}$ | $\frac{1715699412969}{143319222080}$ |
| $\Pi(E_{i,R}^{S})$ | $\frac{4768081}{900000}$ | $\frac{2341}{360}$ | $\frac{368}{45}$ |
| $\Pi(E_{AC}^{D})$ | $\frac{7936951308287822117273821}{668617321069327827000000}$ | $\frac{76652231776414975567}{6219696009947235600}$ | $\frac{866116639958220538501}{66861732106932782700}$ |
| $\Pi(E_{BC}^{D})$ | $\frac{3830685357340996224510875010083476273166901}{248360623352180340513216267553634405000000}$ | $\frac{853059608781817187910313457194864389}{53728636744657726449587077891538000}$ | $\frac{409964007220628164431301597255623501981}{24836062335218034051321626755363440500}$ |
| $\Pi(E_{AR}^{D})$ | $\frac{14208215504629553}{1823940230400000}$ | $\frac{6758412278297}{729576092160}$ | $\frac{2056033035089}{182394023040}$ |
| $\Pi(E_{BR}^{D})$ | $\frac{2759307782200958273}{362356125772800000}$ | $\frac{254404629670549}{28988490061824}$ | $\frac{370140726880121}{36235612577280}$ |

**Note:** $\Pi(E_{BC}^{D})>\Pi(E_{AC}^{D})>\Pi(E_{i,C}^{S})>\Pi(E_{AR}^{D})>\Pi(E_{BR}^{D})>\Pi(E_{i,R}^{S})$

References

[1] Ma D, Song H, Zhao J, Zhu Y. Research on behavior-based pricing strategies and contractual models for service differentiated e-commerce platforms. China Manag Sci. 2023;31(2):215-225.

[2] Liu W, Zhang J. Research on price discrimination strategy considering consumers' expected regret. China Manag Sci. 2018;26(5):1-8.
