# Supplementary material for: Two-period e-commerce platforms operation strategies considering the difference in product quality perception
Source: PLoS One. 2026 Mar 26;21(3):e0327280. doi: 10.1371/journal.pone.0327280 (PMC13020826; doi:10.1371/journal.pone.0327280)
Supplement: Attachment — Submitted filename: Response to Reviewers.docx [file pone.0327280.s002.docx]

**Response to the Reviewer’s Comments**

**Manuscript ID:** PONE-D-25-31916R1

**Title:** Two-period e-commerce platforms operation strategies considering the difference in product quality perception

Dear Editors and Reviewers,

We appreciate the opportunity to revise our manuscript titled “Two-period e-commerce platforms operation strategies considering the difference in product quality perception” and are grateful for the insight comments provided by the reviewers. These comments are all valuable and very helpful for revising and improving our paper, as well as the important guiding significance to our research. In the following, we have provided detailed responses to each of the reviewer’s comments. Revised portions are marked in red on the paper. Additionally, we have conducted a comprehensive revision of the entire manuscript. In this response letter, the reviewer’s comments are presented in italica, and our corresponding changes and additions to the manuscript are highlighted in red text. We have tried our best to make all revisions clear, and we hope that the revised manuscript meets the requirements for publication.

The funders had no role in study design, data collection and analysis, decision to publish, or preparation of the manuscript.

Here below is our description on revision according to your comments.

**Additional Editor:**

*The paper is about “Two-period e-commerce platforms operation strategies considering the difference in product quality perception”. I have the following comments:*

**Response:** We feel great thanks for your professional review work on our article. As you are concerned, there are several problems that need to be addressed. According to your nice suggestions, we have made extensive corrections to our previous draft, the detailed corrections are listed below.

**Additional Editor Comments:***1. There is no big improvement in e-commerce platforms. How do you prove product quality perception?*

**The authors’ Answer:** Thank you for your valuable comments. we have revised the paper according to your suggestions, and made a reference index in the manuscript. Please refer to the manuscript for details. The relevant issues are supplemented as follows:

In response to your questions about e-commerce platform improvement, our study focuses on operational strategy innovation rather than platform technology upgrades. We have constructed a two-period dynamic game framework that simultaneously considers quality perception heterogeneity (H/L) and dual platform competition (four scenarios: S-R, S-C, D-R, D-C). The purpose is to explore how the difference in product quality perception and decision-making order affect pricing, commission rate and profit distribution, and provide strategic guidance for the platform in the context of consumer channel conversion. The author has supplemented the above in the introduction, emphasizing innovation at the strategic level.

**The revised content is as follows:** Currently, research on two-period pricing typically assumes that consumer valuations are fixed or that price is the only motivation for transfer [24,25]. Although some literature has considered consumers' strategic behavior, few have incorporated “perceived quality updates” into dynamic pricing models. Although product quality perception is widely recognized as a key factor influencing consumer decision-making [26,27], most studies focus on the formation mechanisms of quality perception in static environments (such as online reviews and brand awareness), focusing on product quality itself or price competition [8,9]. For example, improving objective product quality through supply chain optimization, quality certification, or technological upgrades [27,28] while ignoring its impact on platform operational strategies (such as pricing and commission models) in multi-cycle dynamic competition [28,29], and systematic exploration of how differences in consumer quality perception affect platform pricing and model selection at the strategic level remains insufficient.

(Page4, Line:64-75)

In building the model, we introduce consumer product quality perception probability$\lambda$ as a core variable. Drawing on existing research, the valuation of consumer product quality perception adopts a discrete value distribution, distinguishing between high experience and low experience, which reflects the differences in product quality perception. As $\lambda$ increases (consumers are more likely to perceive higher quality), the platform can maintain demand and increase profits by raising prices (Theorem 1 and Theorem 2). This positive relationship between price and product quality reflects the platform's strategic enhancement of quality perception. In addition, the numerical simulation results (6.1, 6.2) also show that with the increase of $\lambda$, the profit of the platform and the manufacturer increases significantly under both modes, which further verifies the promotion effect of product quality perception improvement on the profitability of the platform.

Thank you again for your careful corrections. If you have any questions or suggestions regarding the revised settings, please feel free to let us know. We are willing to provide further explanations or supplementary experimental verification at any time.

**Additional Editor Comments:***2. There are several equations. But how do you prove that those equations are correct? The derivation of each equation is needed in the revised version.*

**The authors’ Answer:** Thank you for your valuable comments. Based on your comments, we have added an equation proof section to the appendix section to supplement the proof of equations in the paper. The specific derivation process is shown in the appendix.

Proofs of Equations

Appendix S11. Proof of Equation (1) to Equation (22)

For Equation (1), using the Hotelling linear urban model, consumers compare the utility difference between the two options: “staying on the original platform” and “switching platforms” in the second period. Assume that the consumer's distance to platform $E_{A}$ is $x$, and to platform $E_{B}$ is $1-x$. As assumed in the model description, only a subset of consumers who perceive low product quality in the first period may switch channels for purchase. Under the self-operated model, the platform offers a price discount to new consumers, with a discount coefficient of. The utility functions for consumers repeating purchases or switching platforms in the second period can be specified as follows:

Utility from repeat purchasing product$A$:$L-b_{AR}^{S}-tx$

Utility from switching to purchase product$B$:$\bar{v}-c_{BR}^{S}-t\left(1-x\right)+\beta^{k}c_{BR}^{S}$

Utility from repeat purchasing product$B$:$L-b_{BR}^{S}-t\left(1-x\right)$

Utility from switching to purchase product$A$:$\bar{v}-c_{AR}^{S}-tx+\beta^{k}c_{AR}^{S}$

Following the approach of Ma et al. [1], we derive the indifference point between consumers repeating and switching purchases in the second period by comparing consumer utilities, as shown in Equations (1) and (2):

The solution yields two utility indifference points: $x_{0}$, representing the threshold where consumers are indifferent between continuing to purchase product$A$ on platform $E_{A}$ and switching to purchase product$B$ on platform $E_{B}$; and $x_{1}$, representing the threshold where consumers are indifferent between continuing to purchase product$B$ on platform $E_{B}$ and switching to purchase product$A$ on platform $E_{A}$, as specified in Equations (3) and (4).

$$x_{0}=\frac{t-b_{AR}^{S}-\lambda(H-L)-c_{BR}^{S}\left(\beta^{S}-1\right)}{2t}$$

$$x_{1}=\frac{t+b_{BR}^{S}+\lambda(H-L)+c_{AR}^{S}\left(\beta^{S}-1\right)}{2t}$$

Strategic consumers make decisions by maximizing their total utility across both periods [1,2]. Therefore, the utility indifference point y in the first period must satisfy the condition where the cumulative utility of "purchasing on platform $E_{A}$ in Period 1 and switching to platform $E_{B}$ in Period 2" equals that of "purchasing on platform $E_{B}$ in Period 1 and switching to platform $E_{A}$ in Period 2."The product$A$ first-period market demand is$d_{A}=\text{y}$, and the second-period market demand includes both repeat and switching consumers, i.e. $D_{A}=\lambda\text{y}+\left(1-\lambda\right)x_{0}+\left(1-\lambda\right)\left(x_{1}-y\right)$; similarly, the product$B$ first-period market demand is$d_{B}=\left(1-\text{y}\right)$, and the second-period market demand is$D_{B}=\lambda(1-\text{y)}+(1-\lambda)(1-x_{1})+(1-\lambda)(y-x_{0})$. The above derivation leads to Equation (5):

$$\bar{v}-c_{BR}^{S}-t\left(1-x_{0}\right)+\beta^{S}c_{BR}^{S}+\bar{v}-a_{AR}^{S}-td_{A}=(\bar{v}-c_{AR}^{S}-tx_{1}+\beta^{S}c_{AR}^{S})+(\bar{v}-a_{BR}^{S}-td_{B})$$

Based on the Nash equilibrium solution, we derive the utility indifference point for purchasing the two products in the first period, as formalized in Equation (6):

$$\text{y}=\frac{t\left(\lambda-2\right)-a_{AR}^{S}\left(\lambda-1\right)+a_{BR}^{S}\left(\lambda-1\right)}{2t\left(\lambda-2\right)}$$

The profit of the self-operated platform is calculated as (selling price - wholesale price - price discount) × market demand. Since only consumers who switch platforms in the second period are eligible for the price discount, we derive the platform's second-period profit and total two-period profit, as specified in Equations (7)-(10):

$$\pi(E_{AR}^{S})=\left(b_{AR}^{S}-\omega_{A}^{S}\right)\left(\lambda y+\left(1-\lambda\right)x_{0}\right)+\left(1-\lambda\right)\left(x_{1}-y\right)\left(c_{AR}^{S}-\omega_{A}^{S}-\beta^{S}c_{AR}^{S}\right)$$

 (8)

$$\Pi(E_{AR}^{S})=\left(a_{AR}^{S}-\omega_{A}^{S}\right)d_{A}+\left(b_{AR}^{S}-\omega_{A}^{S}\right)\left(\lambda y+\left(1-\lambda\right)x_{0}\right)+\left(1-\lambda\right)\left(x_{1}-y\right)\left(c_{AR}^{S}-\omega_{A}^{S}-\beta^{S}c_{AR}^{S}\right)$$

$$\Pi(E_{BR}^{S})=d_{B}(a_{BR}^{S}-\omega_{B}^{S})+(b_{BR}^{S}-\omega_{B}^{S})(\lambda(1-\text{y)+(1-}\lambda)(1-x_{1}))+(1-\lambda)(y-x_{0})(c_{BR}^{S}-\omega_{B}^{S}-\beta^{S}c_{BR}^{S})$$

The manufacturer$M_{j}$ generates profit by charging the platform wholesale prices, from which we derive the manufacturer's second-period profit and total two-period profit, as formalized in Equations (11)-(14):

$$\pi(M_{AR}^{S})=\omega_{A}^{S}D_{A}$$

$$\pi(M_{BR}^{S})=w_{B}^{S}D_{B}$$

$$\Pi(M_{AR}^{S})=\omega_{A}^{S}d_{A}+\omega_{A}^{S}D_{A}$$

$$\Pi(M_{BR}^{S})=\omega_{B}^{S}d_{B}+\omega_{B}^{S}D_{B}$$

Using backward induction, we obtain the Nash equilibrium solution as specified in Equations (15)-(22), with the detailed derivation process provided in Appendix S1.

*Appendix S12. Proof of Equation (23) to Equation (41)*

Similar to Appendix S1, both platforms make decisions simultaneously, with the key difference lying in their operational models. In the commission-based model, the platform gives consumers a discount amount $e_{i}^{k},i=(A,B),k=(S,D)$to attract consumers. The utility functions for consumers repeating purchases or switching platforms in the second period can be specified as follows:

Utility from repeat purchasing product$A$:$L-b_{AC}^{S}-tx_{0}$

Utility from switching to purchase product$B$:$\bar{v}-c_{BC}^{S}-t\left(1-x_{0}\right)+e_{i}^{k}$

Utility from repeat purchasing product$B$:$L-b_{BC}^{S}-t\left(1-x_{1}\right)$

Utility from switching to purchase product$A$:

By comparing consumer utilities, we derive the utility indifference points for consumers repeating purchases or switching platforms in the second period, as formalized in Equations (23) and (24):

The solution yields two utility indifference points: $x_{0}$, representing the threshold where consumers are indifferent between continuing to purchase product$A$ on platform $E_{A}$ and switching to purchase product$B$ on platform $E_{B}$; and $x_{1}$, representing the threshold where consumers are indifferent between continuing to purchase product$B$ on platform $E_{B}$ and switching to purchase product$A$ on platform $E_{A}$, as specified in Equations (25) and (26).

$$x_{0}=\frac{t-(H-L)\lambda-b_{AC}^{S}+c_{BC}^{S}-e_{B}^{S}}{2t}$$

$$x_{1}=\frac{t+(H-L)\lambda+b_{BC}^{S}-c_{AC}^{S}+e_{A}^{S}}{2t}$$

Following the approach in Appendix S11, the utility indifference point$y$ in the first period must satisfy the condition where the cumulative utility of "purchasing on platform $E_{A}$ in Period 1 and switching to platform $E_{B}$ in Period 2" equals that of "purchasing on platform $E_{B}$ in Period 1 and switching to platform $E_{A}$ in Period 2."As demonstrated in Equations (27) and (28):

|  |  |
| --- | --- |

$$\text{y}=\frac{6t\left(\lambda-2\right)-4\left(\lambda-1\right)a_{AC}^{S}+4\left(\lambda-1\right)a_{BC}^{S}-3\left(\lambda-1\right)\theta_{A}^{S}+3\left(\lambda-1\right)\theta_{B}^{S}}{12t\left(\lambda-2\right)}$$

The profit of the commission-based platform is calculated as (commission - price discount) × market demand, where only consumers who switch platforms in the second period qualify for the price discount. This yields the platform's second-period profit and total two-period profit, as specified in Equations (29)-(32):

$$\pi(E_{AC}^{S})=\theta_{A}^{S}\left(\lambda y+\left(1-\lambda\right)x_{0}\right)+\left(\theta_{A}^{S}-e_{A}^{S}\right)\left(1-\lambda\right)\left(x_{1}-y\right)$$

$$\pi(E_{BC}^{S})=\theta_{B}^{S}\left(\lambda\left(1-\text{y}\right)+\left(1-\lambda\right)\left(1-x_{1}\right)\right)+\left(\theta_{B}^{S}-e_{B}^{S}\right)\left(1-\lambda\right)\left(y-x_{0}\right)$$

$$\Pi(E_{AC}^{S})=\theta_{A}^{S}y+\pi(E_{AC}^{S})$$

$$\Pi(E_{BC}^{S})=\theta_{B}^{S}\left(1-y\right)+\pi(E_{BC}^{S})$$

Under the commission-based model, the manufacturer's profit equals sales revenue minus commissions paid to the platform. This yields the manufacturer's second-period profit and total two-period profit, as formalized in Equations (33)-(36):

$\pi(M_{AC}^{S})=\left(b_{AC}^{S}-\theta_{A}^{S}\right)\left(\lambda y+\left(1-\lambda\right)x_{0}\right)+\left(c_{AC}^{S}-\theta_{A}^{S}\right)\left(1-\lambda\right)\left(x_{1}-y\right)$ $\pi(M_{BC}^{S})=\left(b_{BC}^{S}-\theta_{B}^{S}\right)\left(\lambda\left(1-y\right)+\left(1-\lambda\right)\left(1-x_{1}\right)\right)+\left(c_{BC}^{S}-\theta_{B}^{S}\right)\left(1-\lambda\right)\left(y-x_{0}\right)$

$$\Pi(M_{AC}^{S})=\left(a_{AC}^{S}-\theta_{A}^{S}\right)y+\pi(M_{AC}^{S})$$

$$\Pi(M_{BC}^{S})=\left(a_{BC}^{S}-\theta_{B}^{S}\right)\left(1-\text{y}\right)+\pi(M_{BC}^{S})$$

Through backward induction, we obtain the Nash equilibrium solution as specified in Equations (37)-(41), with the complete derivation process detailed in Appendix S2.

Appendix S13. Proof of Equation (42) to Equation (60)

The proofs for Equations (42)-(60) follow a similar approach to Appendix S11, with the only difference being the platforms' decision sequence, which alters the backward induction process for equilibrium derivation: the leader platform makes decisions first, followed by the follower platform. The detailed proofs are provided in Appendix S3.

Appendix S14. Proof of Equation (61) to Equation (71)

The proofs for Equations (61)-(71) follow a similar approach to Appendix S12, with the only difference being the platforms' decision sequence, which alters the backward induction process for equilibrium derivation: the leader platform makes decisions first, followed by the follower platform. The detailed proofs are provided in Appendix S4.

References

[1] Ma D, Song H, Zhao J, Zhu Y. Research on behavior-based pricing strategies and contractual models for service differentiated e-commerce platforms. China Manag Sci. 2023;31(2):215-225.

[2] Liu W, Zhang J. Research on price discrimination strategy considering consumers' expected regret. China Manag Sci. 2018;26(5):1-8.

(Appendix, Page12-16, Line:220-326)

Once again, thank you for your professional insights and valuable comments. If there are any areas that require further clarification, please do not hesitate to let us know, and we will do our utmost to make timely revisions.

**Additional Editor Comments:***3. How is the authenticity of the model confirmed? How is the data authenticated?*

**The authors’ Answer:** Thank you for your question and valuable suggestions.

Firstly, in terms of model rationality and economic foundations, we use a two-period dynamic game framework to characterize the pricing behavior of platforms and manufacturers. The core assumptions follow the Hotelling linear city model and the bilateral market pricing logic in platform economics [24]. The Nash equilibrium game structure is adopted when the platform makes decisions at the same time, and the Stackelberg game structure is used when the platform makes decisions successively, and the model construction and the decision-making order of participants are in line with the game characteristics. We have expanded the appendix to include detailed derivations, providing supplementary explanations for the construction of the demand functions, consumer utility functions, and profit functions for both platforms and manufacturers in the model, thereby verifying the correctness of the equations. You can find the full proof process in our answer to question 3. In terms of operational model characterization, platforms are divided into commission-based platforms and self-operated platforms. Existing research [17, 33, 68] has verified their practical applicability.

Secondly, we have a solid basis for selecting simulation data. We assigned values to the unit search cost of online shopping $(t)$ and consumers' perceptions of product quality differences $(\Delta s=H-L)$ based on relevant literature [24,53]. You mentioned the data sources of the numerical experiments in question 4, and our answer to question 4 just explains how this article authenticates the data. Considering the space issue, I will not repeat it here, thank you for your understanding.

[17] Abhishek V, Jerath K, Zhang ZJ. Agency Selling or Reselling? Channel Structures in Electronic Retailing. Manag Sci. 2016;62(8):2259-2280. doi:10.1287/mnsc.2015.2230

[24] Liu W, Zhang J. Research on price discrimination strategy considering consumers' expected regret. China Manag Sci. 2018;26(5):1-8.

[33] Bian Y, Cheng W. Research on offline channel strategy and operation mode selection of platform supply chain. China Manag Sci. 2022:1-26.

[53] Ma D, Song H, Zhao J, Zhu Y. Research on behavior-based pricing strategies and contractual models for service differentiated e-commerce platforms. China Manag Sci. 2023;31(2):215-225.

[68] Zhang C, Ma H. Sales model selection and information sharing strategy of dominant e-retailers in a competitive environment. China Manag Sci. 2021;29(12):115-124.

**Additional Editor Comments:***4. What is the data source of the numerical experiment? Please note that the data is sourced from industry, literature, or any other relevant source, indicating whether it is accurate or artificial. Compare the results with the existing models.*

**The authors’ Answer:** Thank you for your correction!

In the numerical experiment part, we assign values to the unit search cost of online shopping $(t)$ and the difference between consumers' perception of product quality$(\Delta s=H-L)$. Ma Dongsheng et al. [53] assume $t=1$ in the Hotelling model, study the impact of service differentiation on the pricing of e-commerce platforms, and compare the equilibrium profits under the self-operated and commission-based models. This paper draws on its approach to assign t to 1 to simplify the model derivation and focus on the core competitive strategy analysis. Xie Guangming et al. [71] conducted an empirical analysis based on data from Amazon China, indicating that the coefficient of variation of word-of-mouth discrepancy for practical attribute products is below 0.2. Liu Weiqi et al. [24] establish $H=6.0$ and $L=1.0\text{ (}\Delta\text{s=5)}$ in the context of pricing discrimination strategies, representing consumer disagreement on the functionality and durability of electronic products (such as headphones). Ma Dongsheng et al. [53] used $\Delta\text{s=8.0}$ in the luxury e-commerce scenario in the service differentiation model to verify the pricing resilience of the leader platform under high differentiation. Therefore, we assign $\Delta\text{s}$to 0.1, 5, and 10, respectively, which represent the three levels of low, medium, and high perceived differences in product quality.

The numerical simulation results in this paper provide a theoretical basis for the selection of platform operating models. The study shows that differences in consumer perceptions of product quality and the order of platform decisions are both factors influencing the selection of platform operating models. Regardless of whether the platform adopts a self-operated business model or a commission-based business model, the platform's profits under simultaneous decision-making are lower than those under sequential decision-making. When competing platforms make decisions simultaneously, choosing a self-operated model can yield higher profits; when competing platforms make decisions sequentially, choosing a commission-based model allows follower platforms to earn higher profits.

Existing research indicates that, in addition to channel competition, commission fees are also an important factor influencing retailers' choice of operating model [31]. When there are differences in market competitiveness among companies, leading e-retailers should choose the self-operated model [68]. Zhen and Gu [32] considered the showrooming behavior of consumers when purchasing experiential products. They found that when the commission ratio is within a reasonable range, retailers choose the commission-based model if the consumer satisfaction rate of the product is very low, and conversely, they choose the self-operated model.

[24] Liu W, Zhang J. Research on price discrimination strategy considering consumers' expected regret. China Manag Sci. 2018;26(5):1-8.

[31] Zhang Z, Liu W. Research on channel mode selection of online platform product competition. China Manag Sci. 2023;31(2):245-254.

[32] Zhen X, Gu Y. Research on retailers' sales model selection considering consumers' showrooming behavior. Syst Eng Theory Pract. 2023;43(7):1996-2014. doi:10.12011/SETP2022-1971

[53] Ma D, Song H, Zhao J, Zhu Y. Research on behavior-based pricing strategies and contractual models for service differentiated e-commerce platforms. China Manag Sci. 2023;31(2):215-225.

[68] Zhang C, Ma H. Sales model selection and information sharing strategy of dominant e-retailers in a competitive environment. China Manag Sci. 2021;29(12):115-124.

[71] Xie G, Qiu D, Jiang Y. A Research on the e-WOM's Dispersive Coefficient of Variation from the Perspective of Endogeneity. Management Review. 2018,30(04):94-10.

**Additional Editor Comments:** *5. Avoid writing abbreviations in the abstract. Rewrite the abstract, mentioning the results obtained from your study. What research questions are you trying to solve in this model?*

**The authors’ Answer:** Sincerely thank the reviewers for their careful reading and valuable comments. We have rewritten the abstract based on the reviewers' suggestions, including the research conclusions of this paper and the research questions we try to address in this model. please refer to the manuscript for details.

**The revised content is as follows:** The difference in consumer perceptions of product quality influences consumers' propensity to purchase through various channels, thereby affecting the profit of e-commerce platforms and manufacturers. This factor is a critical consideration for e-commerce platforms when formulating their operational strategies in a multi-channel competitive environment. This study focuses on a product retail supply chain comprising two e -commerce platforms, two manufacturers, and strategic consumers. Innovatively, we construct a two-period dynamic pricing game model to compare commission-based and self-operated models for the platforms and deeply explore the impact of consumers' product quality perception differences on pricing strategies and platform operation model choices. Four scenarios are considered: self-operated model under simultaneous decision-making, commission-based model under simultaneous decision-making, self-operated model under successive decision-making, and commission-based model under successive decision-making. Results show that as the probability of consumers perceiving high-quality products increases, both e-commerce platforms and manufacturers can enhance their profit by raising prices. Moreover, when platforms make decisions simultaneously, they should choose the self-operated model. In contrast, when platforms make decisions sequentially, the choice of platform operation mode must account for both the difference in perceived product quality and the probability of consumers obtaining high-quality perceptions. In particular, in the context of successive decision-making, after attracting consumers through the low-price strategy in the first period, the leader platform can still effectively maintain market share even if the price is raised in the second period.

(Page1-2, Line:10-29)

**Additional Editor Comments:***6. What is the novelty of this study? Prove it by comparing it with the related newly introduced study. In the result section, put a subsection of discussions, where the main results obtained in this model are presented.*

**The authors’ Answer:** We sincerely appreciate your valuable feedback. We have adopted the reviewers' suggestions and revised and improved the conclusion section to obtain more details and enrich the depth and breadth of our analysis.

**The revised content is as follows:** Different from previous literature that focused on objective quality improvement or static price discrimination [28,31], this paper combines a two-period model to make consumer quality perception differences an endogenous core variable in two-period dynamic games, while also considering the interaction between platform operating models (self-operated/commission-based) and decision-making sequences (simultaneous/sequential). We respectively construct dynamic pricing game models under commission-based and self-operated models, and conduct a comparative analysis of decision variables such as product prices, commission rates, and discount amounts in four cases: self-operated model under simultaneous decision-making by both platforms (S-R), commission-based model under simultaneous decision-making by both platforms (S-C), self-operated model under sequential decision-making by both platforms (D-R), and commission-based model under sequential decision-making by both platforms (D-C). We explore the impact of consumer product quality perception differences and channel competition on supply chain equilibrium decision-making, and explore the impact of the probability change of consumers' high-quality perception on platform profit, manufacturers' profit and platform operation mode choices through numerical simulation, so as to provide a theoretical basis for platforms to formulate effective operation strategies under different market factors.

The main conclusions of this study are as follows: 1) Both in the commission-based (prices are decided by the manufacturers) model and the proprietary (prices are decided by the platforms) model, when the probability of consumers obtaining high-quality perception is high, the manufacturing enterprises and the platforms can increase the market price to obtain higher profit. 2) In the commission-based model, platforms can attract consumers by raising the commission rate and offering greater price concessions, thereby enhancing their profit and market competitiveness. 3) After locking in consumers with low prices in the first cycle, the leader platform can maintain its market share in the second cycle even if it raises the price for repeat purchases, thanks to its perceived advantage. This explains the sustainability of the “subsidize first, raise prices later” strategy in reality. 4)The widening perception gap has a positive effect on the profits of both platforms and manufacturers, and this conclusion holds true in both self-operated and commission-based models, indicating that differentiated experiences have become a new profit lever. 5) When platforms compete simultaneously, higher profit can be achieved by adopting a self-managed model. In sequential decision-making scenarios, the leader platform must consider both product quality differences and the probability of consumers perceiving high quality in selecting an operating model. Follower platforms can achieve higher profit when the leader platform prefers the commission-based model.

(Page46-47, Line:826-859)

Thank you again for your careful comments. If you have any questions or suggestions regarding the revised content, please continue to provide your feedback, and we will be happy to provide further clarification at any time.

**Additional Editor Comments:***7. Before the conclusion section, put the managerial sections and write how this study will benefit which type of industry and why. What are the limitations of studying? Put references in each future extension.*

**The authors’ Answer:** Thank you for your valuable comments. We have added management insights after the conclusion summary, clarifying the guidance role of the research conclusions for decision-making by e-commerce platforms and manufacturers, and supplementing the practical significance of this research for the industry. Furthermore, we have combined existing research to further discuss the limitations of this study, providing a reference for future expansion studies.

**The revised content is as follows:** This study has important management implications for e-commerce platforms and manufacturers. Self-operated platforms should continue to invest in quality traceability systems and brand storytelling to amplify consumers' perception of high quality, thereby supporting subsequent price increases. Commission-based platforms can use high commission income to subsidize new users, attract low-perception migrants with coupons, and encourage manufacturers to disclose quality signals, creating a virtuous cycle. For manufacturers, when working with commission-based platforms, they can enhance consumer perception by strengthening digital quality control, thereby gaining higher commission tolerance and pricing flexibility from the platform. When working with self-operated platforms, they should focus on ensuring stable supply so that the platform can implement dynamic high-price strategies. This study is not only applicable to e-commerce platforms but also has significant implications for other industries such as retail and services that face multi-channel competition and consumer strategic behavior. Among these, the 3C electronics, beauty, and luxury goods categories stand out for their rapid iteration. In these industries, consumers have a fast-paced perception of updates and high price elasticity, and adopting the strategies outlined in this paper can quickly amplify profits.

We examine the impact of price factors on channel competition within the context of variations in consumer perception of product quality. However, it is essential to acknowledge that buyer feedback [72] and return rates [73] also play significant roles in consumers' decisions regarding purchasing channels. Consequently, future research endeavors will delve deeper into online reviews and return policies to more precisely forecast market responses. Such research will comprehensively encapsulate the multifaceted factors influencing consumer perception of product quality, thereby affording enterprises more profound market insights and strategic directives.

(Page47-48, Line:860-882)

**Additional Editor Comments:** *8. The way of abstract writing is good, but it should contain the details of the study and the findings in a very constructive manner. The entire paper contains several grammatical and spelling errors in English. Could you correct them all?*

**The authors’ Answer:** Thanks to your valuable comments and affirmation of the summary writing method. We have revised the abstract section in accordance with your suggestions, enhancing it with additional research details and significant findings. The specific corrections are as follows:

**The revised content is as follows:** The difference in consumer perceptions of product quality influences consumers' propensity to purchase through various channels, thereby affecting the profit of e-commerce platforms and manufacturers. This factor is a critical consideration for e-commerce platforms when formulating their operational strategies in a multi-channel competitive environment. This study focuses on a product retail supply chain comprising two e -commerce platforms, two manufacturers, and strategic consumers. Innovatively, we construct a two-period dynamic pricing game model to compare commission-based and self-operated models for the platforms and deeply explore the impact of consumers' product quality perception differences on pricing strategies and platform operation model choices. Four scenarios are considered: self-operated model under simultaneous decision-making, commission-based model under simultaneous decision-making, self-operated model under successive decision-making, and commission-based model under successive decision-making. Results show that as the probability of consumers perceiving high-quality products increases, both e-commerce platforms and manufacturers can enhance their profit by raising prices. Moreover, when platforms make decisions simultaneously, they should choose the self-operated model. In contrast, when platforms make decisions sequentially, the choice of platform operation mode must account for both the difference in perceived product quality and the probability of consumers obtaining high-quality perceptions. In particular, in the context of successive decision-making, after attracting consumers through the low-price strategy in the first period, the leader platform can still effectively maintain market share even if the price is raised in the second period.

(Page1-2, Line:10-29)

We are grateful for the reviewer's meticulous attention to detail, which has helped us refine our work to a higher standard. We are sorry for our carelessness and thank you for your reminder. Based on your comments, we have made the corrections and marked in red in the revised paper. We appreciate for reviewers’ warm work earnestly and hope that the correction will meet with approval.

This factor is a crucial consideration for e-commerce platforms when devising their operational strategies in a multi-channel competitive environment.

This factor is a critical consideration for e-commerce platforms when formulating their operational strategies in a multi-channel competitive environment. (Page:1, Line:12-13)

In Section 4, we propose a theoretical model framework for the combination of four strategies, and derive the equilibrium solutions under the four operational strategies through game equilibrium analysis;

In Section 4, we develop a theoretical framework encompassing four strategic combinations and derive their respective equilibrium solutions through game-theoretic analysis; (Page:6, Line:108-110)

The platform $E_{i}$ attracts consumers from other channels (new consumers) to the channel through the purchase price of the product in the second period, using a price discrimination strategy.

The platform $E_{i}$ employs a price discrimination strategy to attract consumers from competing channels (new consumers) by adjusting the product's purchase price in the second period. (Page:12, Line:264-266)

We construct a pricing game model under the two-period self-operated mode, in which two platforms make decisions simultaneously.

We develop a two-period pricing game under the self-operated mode, where both platforms move simultaneously. (Page:15, Line:290-291)

We construct a pricing game model under the two-period self-management mode, in which the two platforms make decisions successively.

We establish a two-period pricing game under the self-operated mode, where platforms decide sequentially. (Page:20, Line:369-370)

According to Equation (1) and Equation (2), consumers' utility nondifferential points for sticking with the original product and switching to the new one in the second cycle are as follows:

Based on Equations (1) and (2), the utility indifference points for consumers choosing between repeat purchases and switching purchases in the second cycle are as follows: (Page:20, Line:377-378)

**Additional Editor Comments:** *9. The research question is established in the introduction section. It should be rewritten. The introduction should be explained constructively but not in a lengthy manner. The introduction should be divided into paragraphs based on the research gap and the concept of the constructive model.*

**The authors’ Answer:** We sincerely appreciate your valuable feedback and thanks for your correction. We have carefully revised the manuscript based on your comments, following the logic of “research necessity-research gaps-research directions.” We have divided it into multiple paragraphs based on the concepts of research gaps and constructive models, and restructured the introduction section.

**The revised content is as follows:** Currently, research on two-period pricing typically assumes that consumer valuations are fixed or that price is the only motivation for transfer [24,25]. Although some literature has considered consumers' strategic behavior, few have incorporated “perceived quality updates” into dynamic pricing models. Although product quality perception is widely recognized as a key factor influencing consumer decision-making [26,27], most studies focus on the formation mechanisms of quality perception in static environments (such as online reviews and brand awareness), focusing on product quality itself or price competition [8,9]. For example, improving objective product quality through supply chain optimization, quality certification, or technological upgrades [27,28] while ignoring its impact on platform operational strategies (such as pricing and commission models) in multi-cycle dynamic competition [28,29], and systematic exploration of how differences in consumer quality perception affect platform pricing and model selection at the strategic level remains insufficient.

Although there is a wealth of research on price discrimination and platform operating models [17,30], studies on the choice between platform self-operation and commission models have mainly focused on aspects such as channel power, commission rates, and showroom behavior [31,32]. Few scholars have combined research on consumer quality perception differences with dynamic two-period game analysis, and have not fully explored how consumers' channel switching behavior based on quality perception in the second period affects the platform's optimal pricing and model selection. Most of the current research is based on a single platform or static model [33], and there's a lack of theoretical modeling of dynamic interactions under dual-platform competition. Few scholars have done a systematic analysis of the interaction between quality perception differences and platform decision order.

In response to the shortcomings of existing research, we construct a two-period dynamic game model, which classifies strategic consumers into two types of high and low perception according to the different valuations of consumers' perception of product quality [24,34]. Considering the impact of price factors on channel competition in the context of differences in consumers' perception of product quality, we divide the enterprise strategy portfolio into four types: self-operated model under simultaneous decision-making by both platforms (S-R), commission-based model under simultaneous decision-making by both platforms (S-C), self-operated model under sequential decision-making by both platforms (D-R), and commission-based model under sequential decision-making by both platforms (D-C). Considering the uncertainty factors of product price and consumer product quality perception at different stages, we provide a new theoretical framework for studying the operational decisions of different types of platforms in different periods, which helps platforms and manufacturers to under-stand the market dynamics and consumer behaviors better so that they can flexibly adjust their pricing strategies, commission policies and incentives in actual operations. This paper clearly answers the following research questions: 1) the choice of pricing strategies of manufacturers and platforms under different operating modes. 2) The impact of commission rate and discount amount on decision-making under different decision sequences of enterprises under the same operating mode. 3) The impact of the probability changes of consumer product quality perception difference on the profit of manufacturers and platforms.

(Page4-5, Line:64-104)

Thank you again for your careful comments. If you have any questions or suggestions regarding the revised content, please continue to provide your feedback, and we will be happy to provide further clarification at any time.

**Additional Editor Comments:** *10. The introduction must be broken into three parts: the necessity of the research, the research gap, and the research orientation. The literature review must be based on keywords.*

**The authors’ Answer:** Thanks to your valuable comments. We think this is an excellent suggestion and have rewritten this part according to the Reviewer’s suggestion. The revised introduction is based on keywords and is divided into three sections: the necessity of the research, research gaps, and research directions. The change can be found in the revised manuscript.

**The revised content is as follows:** The transformation of e-commerce platforms from a single-channel to multi-channel coexistence has intensified channel competition [1,2], and consumers frequently switch purchasing channels during the multi-period buying process to seek the best choice [3]. For example, Walmart's Walmart+ Assist program and Amazon Prime compete for users with exclusive discounts [4], demonstrating the prevalence of consumer switching between different platforms in a multi-channel coexistence environment. Especially during large-scale promotional events such as "Double 11", the number of users and orders on Jingdong and Tmall increased significantly [5], highlighting consumers' behavior of searching for the best purchasing channels across multiple platforms. Consumer strategy-based purchasing behavior places higher demands on the operational strategies of e-commerce platforms.

It has been shown that during multiple product purchases, consumer buying behavior exhibits strategic consumption characteristics, where product quality [6], the price [7,8], service [9], and factors such as out-of-stock [10], utility [11], retailer-driven [12], and discount period cut-off [13] can be the determinants of consumers' switching channels, with the price factor playing a significant role. Strategic consumers tend to favor more competitively priced channels and seek discounts and promotions more aggressively [14,15]. Manufacturers and platforms adopt price discrimination strategies to enhance competitiveness [16]. E-commerce platforms can be categorized into self-operated platforms and commission-based platforms according to the different roles they play in sales [17]. Jingdong has created a self-operated brand called "Made in Jingdong", and at the same time allows third-party merchants to set up and operate their own shops [18]. Under the self-operated model, platforms attract consumers through price discounts. For example, Apple launched the “Apple Trade In” under the national subsidy policy in May 2025, which allows consumers to purchase iPhones from Jingdong with both national subsidies and trade-in subsidies [19]. Under the commission-based model, platforms attract consumers by issuing coupons. For instance, Tmall invested a total of 30 billion in consumer coupons during the "Double 11" period in 2024, which boosted consumers' incentives to shop on Tmall [20]. Through in-depth analysis of the behavioral patterns of target consumer groups, e-commerce platforms can accurately grasp their consumption psychology and purchasing decision-making process [21,22]. At the same time, the platforms develop operational strategies to attract and motivate consumers to make purchases, which in turn drives the platform's profit growth [23].

Currently, research on two-period pricing typically assumes that consumer valuations are fixed or that price is the only motivation for transfer [24,25]. Although some literature has considered consumers' strategic behavior, few have incorporated “perceived quality updates” into dynamic pricing models. Although product quality perception is widely recognized as a key factor influencing consumer decision-making [26,27], most studies focus on the formation mechanisms of quality perception in static environments (such as online reviews and brand awareness), focusing on product quality itself or price competition [8,9]. For example, improving objective product quality through supply chain optimization, quality certification, or technological upgrades [27,28] while ignoring its impact on platform operational strategies (such as pricing and commission models) in multi-cycle dynamic competition [28,29], and systematic exploration of how differences in consumer quality perception affect platform pricing and model selection at the strategic level remains insufficient.

Although there is a wealth of research on price discrimination and platform operating models [17,30], studies on the choice between platform self-operation and commission models have mainly focused on aspects such as channel power, commission rates, and showroom behavior [31,32]. Few scholars have combined research on consumer quality perception differences with dynamic two-period game analysis, and have not fully explored how consumers' channel switching behavior based on quality perception in the second period affects the platform's optimal pricing and model selection. Most of the current research is based on a single platform or static model [33], and there's a lack of theoretical modeling of dynamic interactions under dual-platform competition. Few scholars have done a systematic analysis of the interaction between quality perception differences and platform decision order.

In response to the shortcomings of existing research, we construct a two-period dynamic game model, which classifies strategic consumers into two types of high and low perception according to the different valuations of consumers' perception of product quality [24,34]. Considering the impact of price factors on channel competition in the context of differences in consumers' perception of product quality, we divide the enterprise strategy portfolio into four types: self-operated model under simultaneous decision-making by both platforms (S-R), commission-based model under simultaneous decision-making by both platforms (S-C), self-operated model under sequential decision-making by both platforms (D-R), and commission-based model under sequential decision-making by both platforms (D-C). Considering the uncertainty factors of product price and consumer product quality perception at different stages, we provide a new theoretical framework for studying the operational decisions of different types of platforms in different periods, which helps platforms and manufacturers to under-stand the market dynamics and consumer behaviors better so that they can flexibly adjust their pricing strategies, commission policies and incentives in actual operations. This paper clearly answers the following research questions: 1) the choice of pricing strategies of manufacturers and platforms under different operating modes. 2) The impact of commission rate and discount amount on decision-making under different decision sequences of enterprises under the same operating mode. 3) The impact of the probability changes of consumer product quality perception difference on the profit of manufacturers and platforms.

The rest of the paper is organized as follows. In Section 2, we systematically sort out the theoretical literature related to this study and establishes the theoretical base of the study; In Section 3, we construct the basic framework of the mathematical model, clarify the assumptions of the research problem and standardizes the model parameter notation system; In Section 4, we develop a theoretical framework encompassing four strategic combinations and derive their respective equilibrium solutions through game-theoretic analysis; In Section 5, we conduct a multi-dimensional comparative study, focusing on the analysis of the dynamic changes of product full-period pricing, channel incentive strategy and supply chain profit distribution under the self-management mode and commission mode, and reveal the role of quality perception difference and channel competition intensity on the platform's operation mode selection and the implementation effect of the price discrimination strategy; In Section 6, we use numerical simulation to verify the validity of the theoretical model, and through sensitivity analysis, explore the influence of key parameter changes on supply chain equilibrium decision-making and optimal profit, and provide model support and suggestions for the selection of optimal operation strategies for e-commerce platforms under different circumstances; In Section 7,we summarize the main findings and put forward the managerial implications. The equilibrium-solving process and proof of theorems in this paper are shown in the appendix.

(Page2-6, Line:33-121)

**Additional Editor Comments:** *11. Keywords should be in the direction of the research. Write assumptions in more detail with references if available. Please write the significant findings in the conclusions. Do not mention all assumptions that have been indicated within the model.*

**The authors’ Answer:** We sincerely appreciate the time and effort you have dedicated to reviewing our manuscript. Your insightful comments and constructive suggestions have significantly improved the quality of our work. Below, we provide a point-by-point response to your feedback.

*a. Keywords should be in the direction of the research. Write assumptions in more detail with references if available.*

**Response a:** After a thorough consideration of the core and direction of our research, we meticulously revised the keywords, changing "dynamic pricing" to "price discrimination". "Dynamic pricing" is too general and can be expanded in too many directions, and the updated keywords better reflect the key topics of the research. In the introduction, we demonstrate the price discrimination strategy under different platform operation models by giving examples. In the literature review section, we organized the existing research based on "price discrimination" and summarized the different types of price discrimination strategies employed by online retailers, namely proportional preference and monetary preference, which provided a theoretical basis for constructing the consumer utility function in this paper. It can be seen that "price discrimination" is more suitable as the keyword of this article.

**The revised content is as follows:** Strategic consumers tend to favor more competitively priced channels and seek discounts and promotions more aggressively [14,15]. Manufacturers and platforms adopt price discrimination strategies to enhance competitiveness [16]. E-commerce platforms can be categorized into self-operated platforms and commission-based platforms according to the different roles they play in sales [17]. Jingdong has created a self-operated brand called "Made in Jingdong", and at the same time allows third-party merchants to set up and operate their own shops [18]. Under the self-operated model, platforms attract consumers through price discounts. For example, Apple launched the “Apple Trade In” under the national subsidy policy in May 2025, which allows consumers to purchase iPhones from Jingdong with both national subsidies and trade-in subsidies [19]. Under the commission-based model, platforms attract consumers by issuing coupons. For instance, Tmall invested a total of 30 billion in consumer coupons during the "Double 11" period in 2024, which boosted consumers' incentives to shop on Tmall [20].

(Page3, Line:47-59)

Strategic consumers dynamically adjust their purchasing channels based on price and perceived product quality. Online retailers have increasingly adopted price discrimination strategies to reward new consumers and incentivize channel-switching behavior in product purchases [24]. Furthermore, consumer segments can be categorized as high-value and low-value groups based on their purchasing patterns [52,53]. Existing research classifies discount strategies into proportional preference and monetary preference [14,54,55]. Coupon distribution can effectively attract new consumers [15], bring stronger consumer engagement [56], increase consumer purchase impulse [57] and promote social empowerment [58], etc. Discount rate and coupon ease of use affect consumer purchase decisions [59]. Zhang et al. [55] empirically demonstrate that proportional discounts are more effective for low-grade products, whereas monetary discounts better suit high-grade products. Zhang et al. [60] established two promotional models, price discount and cash coupon, which are decided by the platform and consider the merchant's advertisement investment strategy, and found that the platform should use the cash coupon strategy when the transaction rate is high, and the price of the products is low. Otherwise, it should use the price discount strategy. By comparing the two scenarios of platform-issued and non-coupon issued, Wang and Yang [61] found that platform-issued coupons are beneficial to the platform and manufacturers' profit but may harm retailers' interests. Chen et al. [62] further reveal that intensified price discounts diminish retailers’ incentives to invest in advertising when online and offline channels compete on both price and promotional efforts. Li et al. [63] discussed the issue of issuing coupons in the online channel under omnichannel retailing. They found that retailers should implement a low-price strategy in the pure online channel to attract consumers with high travelling costs and issue coupons with substantial discounts in the BOPS (order online, pick up offline) channel to attract consumers with a high perceived value of product experience. In addition, Li et al. [64] analyzed the optimal promotional strategies of manufacturers and retailers before and after the introduction of private labels by retailers for an omnichannel supply chain providing BOPS services. The implementation of these promotional strategies can stimulate consumers' purchasing behavior to a certain extent and, at the same time, play a role in pulling new products and promotions [15,65]. These studies mostly assume that consumer valuations are exogenous, ignoring the dynamic impact of differences in quality perceptions on price sensitivity [24]. We combine a two-period model to analyze how consumers' channel switching behavior based on quality perceptions affects product pricing.

(Page8-9, Line:159-190)

*b. Please write the significant findings in the conclusions. Do not mention all assumptions that have been indicated within the model.*

**Response b:** Thank you very much for your valuable feedback. In response to your suggestions, we have carefully revised the conclusion section. In the revised conclusions, we clearly summarized the key findings of the study, emphasizing their guiding significance for the choice of platform operational models, while omitting the mention of all the assumptions indicated in the model.

**The revised content is as follows:** The main conclusions of this study are as follows: 1) Both in the commission-based (prices are decided by the manufacturers) model and the proprietary (prices are decided by the platforms) model, when the probability of consumers obtaining high-quality perception is high, the manufacturing enterprises and the platforms can increase the market price to obtain higher profit. 2) In the commission-based model, platforms can attract consumers by raising the commission rate and offering greater price concessions, thereby enhancing their profit and market competitiveness. 3) After locking in consumers with low prices in the first cycle, the leader platform can maintain its market share in the second cycle even if it raises the price for repeat purchases, thanks to its perceived advantage. This explains the sustainability of the “subsidize first, raise prices later” strategy in reality. 4)The widening perception gap has a positive effect on the profits of both platforms and manufacturers, and this conclusion holds true in both self-operated and commission-based models, indicating that differentiated experiences have become a new profit lever. 5) When platforms compete simultaneously, higher profit can be achieved by adopting a self-managed model. In sequential decision-making scenarios, the leader platform must consider both product quality differences and the probability of consumers perceiving high quality in selecting an operating model. Follower platforms can achieve higher profit when the leader platform prefers the commission-based model.

(Page47, Line:843-859)

Thank you again for your valuable comments. Your rigorous attitude and professional perspective greatly enhance the scientific and readability of this research. If there are any further suggestions, we will still accept them with humility and make timely improvements.

**Reviewer #1:**

*The study examines how consumer perceptions of product quality influence purchasing decisions on e-commerce platforms. The study has some merits in the present literature but there are some drawbacks which I have pointed out are given below.*

**Response:** Thank you for the comments and suggestions on the manuscript. We have carefully revised the manuscript and provided the point-by-point response below.

**Reviewers' comments:***1. Although the authors titled it "Two platform operation modes." (a) Self-employed model; (b) Commission-based model," however it appears to be reselling and agency selling modes in two parallel supply chains. If so, why is this type of name defined? Are authors attempted to demonstrate any particular situation that differed from the reselling and agency selling approaches?*

**The authors’ Answer:** Thank you for your correction!

As you can understand, the self-employed model and the commission-based model correspond to reselling and agency selling, respectively, in two parallel supply chains. The reasons for defining this type name are as follows:

On the one hand, this is done to emphasize pricing power, which is directly related to the equilibrium solution of the game model, the e-commerce platform decides on product pricing under the self-operated model, and the manufacturer decides on product pricing under the commission model [17]. On the other hand, the price discrimination strategies implemented under the two operating models are different, which further affects the utility function and model construction [14,54,55]. Under the self-operated mode, the platform attracts consumers through price discounts with a price discount factor of , In the commission-based model, the platform gives consumers a discount amount $e_{i}^{k}$ to attract consumers. In addition, this article aims to explore which mode of self-employed and commission-based can obtain higher profits in the two markets where the platform makes decisions at the same time and in succession.

We sincerely look forward to your continued guidance. If you have any further questions, please do not hesitate to contact us, and we will immediately make further corrections.

[14] Duan S, Wang Z, Li Y. Relative or Absolute Discounts? Consumer preferences for discount presentation in different purchase types. J Syst Manag. 2021;30(6):1025-1040. doi:10.3969/j.issn1005-2542.2021.06.001

[17] Abhishek V, Jerath K, Zhang ZJ. Agency Selling or Reselling? Channel Structures in Electronic Retailing. Manag Sci. 2016;62(8):2259-2280. doi:10.1287/mnsc.2015.2230

[54] Hao L, Gao C, Jia J. An empirical study on the effect of price discount presentation on promotion effect. Manag World. 2008(10):106-114+126+187-188.

[55] Zhang Z, Fang X, Han B. A study of framing effects on the value of product coupons. Manag Sci. 2011;24(1):47-55. doi:10.3969/j.issn.1672-0334.2011.01.006

**Reviewers' comments:***2. Research questions are unclear. These should be highlighted more explicitly. Are your conclusions suitable for answering these questions?*

**The authors’ Answer:** Thank you for your valuable comments. We have made structural adjustments to the introduction based on your suggestions, highlighting the gaps in existing research at the quality perception strategy level. By clearly stating the research question, a clear theoretical and problem-oriented foundation is laid for subsequent model construction and conclusion derivation.

**The revised content is as follows:** Currently, research on two-period pricing typically assumes that consumer valuations are fixed or that price is the only motivation for transfer [24,25]. Although some literature has considered consumers' strategic behavior, few have incorporated “perceived quality updates” into dynamic pricing models. Although product quality perception is widely recognized as a key factor influencing consumer decision-making [26,27], most studies focus on the formation mechanisms of quality perception in static environments (such as online reviews and brand awareness), focusing on product quality itself or price competition [8,9]. For example, improving objective product quality through supply chain optimization, quality certification, or technological upgrades [27,28] while ignoring its impact on platform operational strategies (such as pricing and commission models) in multi-cycle dynamic competition [28,29], and systematic exploration of how differences in consumer quality perception affect platform pricing and model selection at the strategic level remains insufficient.

Although there is a wealth of research on price discrimination and platform operating models [17,30], studies on the choice between platform self-operation and commission models have mainly focused on aspects such as channel power, commission rates, and showroom behavior [31,32]. Few scholars have combined research on consumer quality perception differences with dynamic two-period game analysis, and have not fully explored how consumers' channel switching behavior based on quality perception in the second period affects the platform's optimal pricing and model selection. Most of the current research is based on a single platform or static model [33], and there's a lack of theoretical modeling of dynamic interactions under dual-platform competition. Few scholars have done a systematic analysis of the interaction between quality perception differences and platform decision order.

In response to the shortcomings of existing research, we construct a two-period dynamic game model, which classifies strategic consumers into two types of high and low perception according to the different valuations of consumers' perception of product quality [24,34]. Considering the impact of price factors on channel competition in the context of differences in consumers' perception of product quality, we divide the enterprise strategy portfolio into four types: self-operated model under simultaneous decision-making by both platforms (S-R), commission-based model under simultaneous decision-making by both platforms (S-C), self-operated model under sequential decision-making by both platforms (D-R), and commission-based model under sequential decision-making by both platforms (D-C). Considering the uncertainty factors of product price and consumer product quality perception at different stages, we provide a new theoretical framework for studying the operational decisions of different types of platforms in different periods, which helps platforms and manufacturers to under-stand the market dynamics and consumer behaviors better so that they can flexibly adjust their pricing strategies, commission policies and incentives in actual operations. This paper clearly answers the following research questions: 1) the choice of pricing strategies of manufacturers and platforms under different operating modes. 2) The impact of commission rate and discount amount on decision-making under different decision sequences of enterprises under the same operating mode. 3) The impact of the probability changes of consumer product quality perception difference on the profit of manufacturers and platforms.

(Page4-5, Line:64-104)

This paper reveals the interactive mechanism between decision-making sequence, quality perception, and operating mode by constructing Stackelberg and Nash equilibrium models. Through multidimensional considerations in the analysis, it provides a theoretical basis and decision-making suggestions for e-commerce platforms to formulate operation strategies. In order to further highlight the research conclusions and their guidance for decision-making by e-commerce platforms and manufacturers, we have revised the conclusions section. We have supplemented the practical significance of this study for the industry. Furthermore, we have combined existing research to further discuss the limitations of this study, providing a reference for future expansion studies.

**The revised content is as follows:** Different from previous literature that focused on objective quality improvement or static price discrimination [28,31], this paper combines a two-period model to make consumer quality perception differences an endogenous core variable in two-period dynamic games, while also considering the interaction between platform operating models (self-operated/commission-based) and decision-making sequences (simultaneous/sequential). We respectively construct dynamic pricing game models under commission-based and self-operated models, and conduct a comparative analysis of decision variables such as product prices, commission rates, and discount amounts in four cases: self-operated model under simultaneous decision-making by both platforms (S-R), commission-based model under simultaneous decision-making by both platforms (S-C), self-operated model under sequential decision-making by both platforms (D-R), and commission-based model under sequential decision-making by both platforms (D-C). We explore the impact of consumer product quality perception differences and channel competition on supply chain equilibrium decision-making, and explore the impact of the probability change of consumers' high-quality perception on platform profit, manufacturers' profit and platform operation mode choices through numerical simulation, so as to provide a theoretical basis for platforms to formulate effective operation strategies under different market factors.

The main conclusions of this study are as follows: 1) Both in the commission-based (prices are decided by the manufacturers) model and the proprietary (prices are decided by the platforms) model, when the probability of consumers obtaining high-quality perception is high, the manufacturing enterprises and the platforms can increase the market price to obtain higher profit. 2) In the commission-based model, platforms can attract consumers by raising the commission rate and offering greater price concessions, thereby enhancing their profit and market competitiveness. 3) After locking in consumers with low prices in the first cycle, the leader platform can maintain its market share in the second cycle even if it raises the price for repeat purchases, thanks to its perceived advantage. This explains the sustainability of the “subsidize first, raise prices later” strategy in reality. 4)The widening perception gap has a positive effect on the profits of both platforms and manufacturers, and this conclusion holds true in both self-operated and commission-based models, indicating that differentiated experiences have become a new profit lever. 5) When platforms compete simultaneously, higher profit can be achieved by adopting a self-managed model. In sequential decision-making scenarios, the leader platform must consider both product quality differences and the probability of consumers perceiving high quality in selecting an operating model. Follower platforms can achieve higher profit when the leader platform prefers the commission-based model.

This study has important management implications for e-commerce platforms and manufacturers. Self-operated platforms should continue to invest in quality traceability systems and brand storytelling to amplify consumers' perception of high quality, thereby supporting subsequent price increases. Commission-based platforms can use high commission income to subsidize new users, attract low-perception migrants with coupons, and encourage manufacturers to disclose quality signals, creating a virtuous cycle. For manufacturers, when working with commission-based platforms, they can enhance consumer perception by strengthening digital quality control, thereby gaining higher commission tolerance and pricing flexibility from the platform. When working with self-operated platforms, they should focus on ensuring stable supply so that the platform can implement dynamic high-price strategies. This study is not only applicable to e-commerce platforms but also has significant implications for other industries such as retail and services that face multi-channel competition and consumer strategic behavior. Among these, the 3C electronics, beauty, and luxury goods categories stand out for their rapid iteration. In these industries, consumers have a fast-paced perception of updates and high price elasticity, and adopting the strategies outlined in this paper can quickly amplify profits.

We examine the impact of price factors on channel competition within the context of variations in consumer perception of product quality. However, it is essential to acknowledge that buyer feedback [72] and return rates [73] also play significant roles in consumers' decisions regarding purchasing channels. Consequently, future research endeavors will delve deeper into online reviews and return policies to more precisely forecast market responses. Such research will comprehensively encapsulate the multifaceted factors influencing consumer perception of product quality, thereby affording enterprises more profound market insights and strategic directives.

(Page46-48, Line:826-882)

Thank you again for your valuable feedback. We sincerely look forward to your continued guidance. If you have any further questions, please do not hesitate to contact us, and we will immediately make further corrections.

**Reviewers' comments:***3. Figure 1 is unclear and difficult to understand. Please redraw it to demonstrate the clarity of your proposed model.*

**The authors’ Answer:** Thank you for your careful review and valuable comments on our manuscript. Your criticism that "Figure 1 is unclear and difficult to understand." is pertinent and profound, and we fully accept and deeply feel guilty. Following your suggestion, we have redrawn Figure 1. In order to make it easier for readers to understand, we have added some necessary symbolic explanations, and the logic is clearer. The revised Figure 1 is shown in the following figure and has replaced the original figure.

**Figure 1 Schematic diagram of the operating model**

(Page13, Line:276-277)

We sincerely look forward to your continued correction, if there are still any unclear points, please do not hesitate to tell us, and we will correct it again immediately.

**Reviewers' comments:***4. The authors claim that two supply chains made choices simultaneously or sequentially. But how do the two players' decisions flow in each supply chain? Are they playing the Nash and Stackelberg game? Nothing is clearly stated in this study. Also, what about the two-period decision sequence? The research provides no obvious answers.*

**The authors’ Answer:** We sincerely appreciate your rigorous questioning of the decision-making sequence and game settings in this manuscript. Your comments have made us keenly aware that there are critical omissions in our model settings and textual logic, which may cause readers to misunderstand the game structure within and between supply chains. We sincerely apologize to the editorial department and to you for this oversight.

This paper considers two scenarios: simultaneous decision-making and sequential decision-making. Specifically, it considers simultaneous decision-making and sequential decision-making separately under the commission-based model and under the self-operated model. When decisions are made simultaneously on both platforms, manufacturers and platforms on two parallel supply chains make decisions at the same time, with no order of precedence, forming a Nash equilibrium. When the two platforms make decisions sequentially, this paper assumes that platform $E_{A}$ is the leader platform and platform $E_{B}$ is the follower platform. The leader platform makes its decision first, followed by the follower platform, forming a Stackelberg game. According to the above problem description, it can be seen that the platform has the following four strategy combinations to choose from: self-operated model under simultaneous decision-making by both platforms (S-R), commission-based model under simultaneous decision-making by both platforms (S-C), self-operated model under sequential decision-making by both platforms (D-R), and commission-based model under sequential decision-making by both platforms (D-C). The two-period decision sequence in the four situations is as follows:

(1) **self-operated model under simultaneous decision-making by both platforms (S-R)**

Manufacturers $M_{A}$ and $M_{B}$decide on wholesale prices simultaneously. Manufacturer $M_{A}$ decides on wholesale price $\omega_{A}^{S}$, and manufacturer $M_{B}$ decides on wholesale price $\omega_{B}^{S}$.

Platform $E_{A}$ and platform $E_{B}$ simultaneously decide on the retail price of the first period products. Platform $E_{A}$ decides on the retail price $a_{AR}^{S}$, and platform $E_{B}$ decides on the retail price $a_{BR}^{S}$.

Platform $E_{A}$ and platform $E_{B}$ simultaneously decide on the retail price of products repurchased in the second period and the retail price of products switched to. Platform $E_{A}$ decides on the retail price $b_{AR}^{S}$ for repeat purchases and the retail price $c_{AR}^{S}$ for switching purchases in the second period, while platform $E_{B}$ decides on the retail price $b_{BR}^{S}$ for repeat purchases and the retail price $c_{BR}^{S}$ for switching purchases in the second period.

(2) **commission-based model under simultaneous decision-making by both platforms (S-C)**

Platform $E_{A}$ and platform $E_{B}$ simultaneously decide on commission rates. Platform $E_{A}$ decides on commission rate $\theta_{A}^{S}$, and platform $E_{B}$ decides on commission rate $\theta_{B}^{S}$.

Manufacturers $M_{A}$ and $M_{B}$simultaneously decide on the retail price of the first period products. Manufacturer $M_{A}$ decides on the retail price $a_{AC}^{S}$, and manufacturer$M_{B}$ decides on the retail price $a_{BC}^{S}$.

Platform $E_{A}$ and platform $E_{B}$ simultaneously decide on the price discount amount for new consumers. Platform $E_{A}$ decides on the price discount $e_{A}^{S}$, and platform $E_{B}$ decides on the price discount amount $e_{B}^{S}$.

Manufacturers $M_{A}$ and $M_{B}$ simultaneously decide on the retail price of products repurchased in the second period and the retail price of products switched to. Manufacturer $M_{A}$ decides on the retail price $b_{AC}^{S}$ for repeat purchases and the retail price $c_{AC}^{S}$ for switching purchases in the second period, while manufacturer $M_{B}$ decides on the retail price $b_{BC}^{S}$ for repeat purchases and the retail price $c_{BC}^{S}$ for switching purchases in the second period.

(3) **self-operated model under sequential decision-making by both platforms (D-R)**

Manufacturers $M_{A}$ and $M_{B}$decide on wholesale prices simultaneously. Manufacturer $M_{A}$ decides on wholesale price $\omega_{A}^{D}$, and manufacturer $M_{B}$ decides on wholesale price $\omega_{B}^{D}$.

Platform $E_{A}$ decides the retail price $a_{AR}^{D}$ for the first period of the product first, and platform $E_{B}$ decides the retail price $a_{BR}^{D}$ for the first period of the product later.

Platform $E_{A}$ decides on the retail price $b_{AR}^{D}$ for repeat purchases and the retail price $c_{AR}^{D}$ for switching purchases in the second period first, and platform $E_{B}$ decides on the retail price $b_{BR}^{D}$ for repeat purchases and the retail price $c_{BR}^{D}$ for switching purchases in the second period later.

(4) **commission-based model under sequential decision-making by both platforms (D-C)**

Platform $E_{A}$ decides on commission rate $\theta_{A}^{D}$ first, and platform $E_{B}$ decides on commission rate $\theta_{B}^{D}$ later.

Manufacturers $M_{A}$ and $M_{B}$simultaneously decide on the retail price of the first period products. Manufacturer $M_{A}$ decides on the retail price $a_{AC}^{D}$, and manufacturer$M_{B}$ decides on the retail price $a_{BC}^{D}$.

Platform $E_{A}$ decides on the price discount $e_{A}^{D}$ first, and platform $E_{B}$ decides on the price discount amount $e_{B}^{D}$ later.

Manufacturers $M_{A}$ and $M_{B}$ simultaneously decide on the retail price of products repurchased in the second period and the retail price of products switched to. Manufacturer $M_{A}$ decides on the retail price $b_{AC}^{D}$ for repeat purchases and the retail price $c_{AC}^{D}$ for switching purchases in the second period, while manufacturer $M_{B}$ decides on the retail price $b_{BC}^{D}$ for repeat purchases and the retail price $c_{BC}^{D}$ for switching purchases in the second period.

We understand that a clear game construction is the cornerstone of model credibility. Thank you again for helping us address this significant vulnerability with such professional insight. If you have any questions or suggestions regarding the revised settings, please feel free to let us know. We are willing to provide further explanations or supplementary experimental verification at any time.

**Reviewers' comments:***5.* *There is no analysis that compares all four scenarios. Provide some analyses that contain all four models' optimal outcomes.*

**The authors’ Answer:** Thank you for your careful review and valuable suggestions on our manuscript. We fully acknowledge your concerns regarding the " there is no analysis that compares all four scenarios." In section 6.3, we conducted independent discussions on the optimal results under simultaneous decision-making and sequential decision-making on the platform. We failed to systematically compare the similarities and differences among the four scenarios from a global perspective, thereby undermining the completeness and persuasiveness of the research conclusions.

In response to your feedback, we have supplemented a new set of integrated analyses. We separately illustrated the platform profits when the perceived differences in product quality are low, medium, and high. By more intuitively comparing and analyzing platform profits under different circumstances, this study is more comprehensive and specific, makes up for the theoretical lack of platform operation mode selection under simultaneous and sequential decision-making, and further complements and improves the conclusions of 6.3.

**The revised content is as follows:**

(a)Low difference in perceived quality

(b)Medium difference in perceived quality

(c)High difference in perceived quality

**Figure 11. Platform operation mode selection.** (“─”indicates the platform profit in the self-managed model, “**┄”** indicates the platform profit in the commission-based model)

(Page43, Line:745-747)

Figures 11(a), 11(b) and 11(c) represent the platform profits under the four operational strategies when the perceived difference in product quality is low, the perceived difference in product quality is medium and the perceived difference in product quality is high, respectively. In this case, the solid line indicates the platform profit in the self-managed model and the dashed line indicates the platform profit in the commission-based model, with different colors to distinguish the platform decision order. The figure above summarizes the profit levels of platform $E_{A}$ and platform $E_{B}$ under four combinations of operational strategies. Theoretically, the four strategies correspond to eight profit curves across two platforms. However, in the “simultaneous decision-making” strategy combination, the optimal response functions of the two platforms are symmetrical, resulting in a unique and identical Nash equilibrium solution. The result is that the profit curves of platform $E_{A}$ and platform $E_{B}$ are completely overlapping. Therefore, only six distinguishable profit curves are actually plotted in the figure. As can be seen from the figure, regardless of whether the platform adopts the self-operating mode or the commission-based mode, the platform profit under simultaneous decision-making is lower than that under sequential decision-making. This is because when making simultaneous decisions, sellers and platforms are unable to observe each other's final strategies and can only make one-off pricing or commission settings through anticipation, leading to a tendency for both sides to adopt more aggressive low-price or low-commission strategies in order to capture demand and compress profit margins. Under sequential decision-making, the later actors can optimize their strategies based on the strategies of the first actors to achieve complementary strategies. It can be seen that the e-commerce platform, while choosing the optimal operation strategy in conjunction with the market environment, can enhance its market positioning by clarifying its strategic positioning and differentiation advantages, branding and trust system construction, and expanding its innovative business model, thus reducing risks such as demand forecasting errors and enhancing the platform's profits.

(Page45-46, Line:800-824)

All added figures and discussions have been integrated into the revised manuscript. We are aware that there may still be omissions, and we urge you to continue to criticize and correct us. Your professional input has greatly enhanced the rigor and depth of this study, and is greatly appreciated!

**Reviewers' comments:***6. Provide a table comparing the optimal numerical results for all scenarios.*

**The authors’ Answer:** Thank you for your suggestions and feedback. Due to the extensive nature of some game equilibrium solutions, a single consolidated table cannot fully display all results. Therefore, we present two new tables in the manuscript: one for the equilibrium solutions under the self-operated platform model, and another for the commission-based platform model. These tables collectively encompass the optimal numerical outcomes across all four operational strategies. Considering that the table is too long to be included in the main text without inconveniencing readers, we have placed it in Appendix S15.The table is as follows:

**Table 3.** The game equilibrium solution of self-operated platforms

| 变量 | S-R | D-R |
| --- | --- | --- |
| $a_{A,g}^{k}$ | $\frac{2}{3}\left((H-L)\lambda+\frac{t\left(90-\lambda\left(67+7\lambda\right)\right)}{\left(\lambda-1\right)\left(2\lambda-13\right)}\right)$ |  |
| $a_{B,g}^{k}$ | $\frac{2}{3}\left((H-L)\lambda+\frac{t\left(90-\lambda\left(67+7\lambda\right)\right)}{\left(\lambda-1\right)\left(2\lambda-13\right)}\right)$ | $\frac{11147t-\frac{11558t}{\lambda-1}+71\lambda\left(59H-59L+2t\right)+\frac{142\left(17t+\lambda\left(L-H+4t\right)\right)}{\lambda\left(3+\lambda\right)-5}-\frac{24\left(497\lambda\left(L-H\right)+4t\left(18446+4589\lambda\right)\right)}{4\lambda\left(5+\lambda\right)-95}}{6816}$ |
| $b_{A,g}^{k}$ | $\frac{1}{3}\left[\frac{t\left(128-\lambda\left(61+24\lambda\right)\right)}{\left(\lambda-1\right)\left(2\lambda-13\right)}-(H-L)\lambda\right]$ | $\begin{matrix} \frac{\left(\text{λ}\left(\text{H-L}\right)\left(\text{1-λ}\right)\left(\text{11145+λ}\left(\text{4λ}\left(\text{3λ}\left(\text{67+8λ}\right)\text{-292}\right)\text{-9379}\right)\right)\right)}{48\left(\lambda-1\right)\left(\lambda\left(3+\lambda\right)-5\right)\left(4\lambda\left(5+\lambda\right)-95\right)}+ \\ \frac{t\left(\lambda\left(109188+\lambda\left(3293+2\lambda\left(2\lambda\left(18\lambda-653\right)-9485\right)\right)\right)-98215\right)}{48\left(\lambda-1\right)\left(\lambda\left(3+\lambda\right)-5\right)\left(4\lambda\left(5+\lambda\right)-95\right)} \end{matrix}$ |
| $b_{B,g}^{k}$ | $\frac{1}{3}\left[\frac{t\left(128-\lambda\left(61+24\lambda\right)\right)}{\left(\lambda-1\right)\left(2\lambda-13\right)}-(H-L)\lambda\right]$ |  |
| $c_{A,g}^{k}$ |  |  |
| $c_{B,g}^{k}$ |  |  |
| $\omega_{A}^{k}$ | $\frac{t\left(34-\lambda\left(19+8\lambda\right)\right)}{\left(\lambda-1\right)\left(2\lambda-13\right)}$ | $\frac{\left(\lambda\left(H-L\right)\left(1-\lambda\right)\left(3+2\lambda\right)\right)+t\left(899+\lambda\left(4\left(\lambda-41\right)\lambda-545\right)\right)}{3\left(\lambda-1\right)\left(4\lambda\left(5+\lambda\right)-95\right)}$ |
| $\omega_{B}^{k}$ | $\frac{t\left(34-\lambda\left(19+8\lambda\right)\right)}{\left(\lambda-1\right)\left(2\lambda-13\right)}$ | $\frac{1021t+\lambda\left(H-L\right)\left(\lambda-1\right)\left(3+2\lambda\right)-t\lambda\left(607+4\lambda\left(55+\lambda\right)\right)}{3\left(\lambda-1\right)\left(4\lambda\left(5+\lambda\right)-95\right)}$ |

**Table 4.** The game equilibrium solution of commission-based platforms

| 变量 | S-C | D-C |
| --- | --- | --- |
| $a_{A,g}^{k}$ | $\frac{2\lambda\left(H-L\right)\left(1-\lambda\right)\left(292+\lambda\left(143+4\lambda\right)\right)+t\left(17416+\lambda\left(+\lambda\left(629+44\lambda\right)-14840\right)\right)}{6\left(\lambda-1\right)\left(\lambda\left(95+4\lambda\right)-512\right)}$ | $\begin{matrix} (((H-L)(1-\lambda)\lambda(\lambda(531431374384+\lambda(278396126768+\lambda(\lambda(4\lambda(4\lambda(418961 \\ +4\lambda(5041+56\lambda))-38673485)-6733603987)-47198747420)))-1022023303312)) \\ +t(-33150803072688+\lambda(56507026168288+\lambda(\lambda(2255244859288+\lambda\text{（}255864960371 \\ +\lambda\text{（}32\lambda\text{（}\lambda\text{（}3359+40\lambda\text{（}757+11\lambda))-33429575)-12444370925)))-27188422935136)) \\ -33150803072688))/(6(\lambda-1)(854387017616+\lambda(\lambda(216667089416+\lambda(6230316280+ \\ \lambda(16\lambda(2\lambda(28903+8\lambda(391+5\lambda))-5547017)-1895596843)))-949817488736))) \end{matrix}$ |
| $a_{B,g}^{k}$ | $\frac{2\lambda\left(H-L\right)\left(1-\lambda\right)\left(292+\lambda\left(143+4\lambda\right)\right)+t\left(17416+\lambda\left(+\lambda\left(629+44\lambda\right)-14840\right)\right)}{6\left(\lambda-1\right)\left(\lambda\left(95+4\lambda\right)-512\right)}$ | $\begin{matrix} (((H-L)(1-\lambda)\lambda(\lambda(533060839504+\lambda(270438966704+\lambda(\lambda(4\lambda(4\lambda(509303 \\ +4\lambda(5311+56\lambda))-27078659)-6057290545)-43930520324)))-1019906049712)) \\ +t(\lambda(56839435942528+\lambda(\lambda(2218476834004+\lambda(255051057845+\lambda(32\lambda(\lambda(3899 \\ +40\lambda(757+11\lambda))-33364964)-12404896709)))-27511199806624))-3296852678 \\ 5488))/(6(\lambda-1)(854387017616+\lambda(\lambda(216667089416+\lambda(6230316280+\lambda(16\lambda(2\lambda\\ (28903+8\lambda(391+5\lambda))-5547017)-1895596843)))-949817488736))) \end{matrix}$ |
| $b_{A,g}^{k}$ | $\frac{\lambda\left(H-L\right)\left(\lambda-1\right)\left(\lambda\left(785+28\lambda\right)-1574\right)+5t\left(\lambda\left(2207+100\lambda\right)-3842\right)}{12\left(1-\lambda\right)\left(\lambda\left(95+4\lambda\right)-512\right)}$ | $\begin{matrix} (\left(H-L\right)\left(\lambda-1\right)\lambda(638781615208+\lambda(\lambda\text{ }(318882388426+\lambda(\lambda(16\lambda(2\lambda(212267+ \\ 8\lambda(1160+13\lambda))-4238899)-3383206091)-5115392899))-891241448548))+t(955 \\ 1765639720+\lambda(\lambda(5269008760574+\lambda(86785414771+8\lambda(8\lambda(4\lambda(93785+\lambda(11365+ \\ 152\lambda))-39407831)-6698702351)))-14226916556060)))/(3\left(1-\lambda\right)(854387017616+ \\ \lambda(\lambda\text{ }(216667089416+\lambda(6230316280+\lambda(16\lambda(2\lambda(28903+8\lambda(391+5\lambda))-5547017- \\ 1895596843)))-949817488736))) \end{matrix}$ |
| $b_{B,g}^{k}$ | $\frac{\lambda\left(H-L\right)\left(\lambda-1\right)\left(\lambda\left(785+28\lambda\right)-1574\right)+5t\left(\lambda\left(2207+100\lambda\right)-3842\right)}{12\left(1-\lambda\right)\left(\lambda\left(95+4\lambda\right)-512\right)}$ | $\begin{matrix} (((H-L)(1-\lambda)\lambda(645486251608+\lambda(\lambda(340129529470+\lambda(\lambda(8\lambda(8\lambda(14\lambda(151+2\lambda) \\ -1097)-26757551)-4879041347)-9344291641))-913194084268)))+t(\lambda(129453983 \\ 85620+\lambda(\lambda(10812994871-8\lambda(\lambda(4\lambda(982885+4\lambda(24425+316\lambda))-315356287)-7312 \\ 402544))-4752960387626))-8974557396920))/(3(\lambda-1)(854387017616+\lambda(\lambda(21666 \\ 7089416+\lambda(6230316280+\lambda(16\lambda(2\lambda(28903+8\lambda(391+5\lambda))-5547017)-1895596843))) \\ -949817488736))) \end{matrix}$ |
| $c_{A,g}^{k}$ | $\frac{\left(\lambda\left(H-L\right)\left(1-\lambda\right)\left(127+4\lambda\right)\left(2+5\lambda\right)\right)+t\left(20750-\lambda\left(14309+652\lambda\right)\right)}{12\left(\lambda-1\right)\left(\lambda\left(95+4\lambda\right)-512\right)}$ |  |
| $c_{B,g}^{k}$ | $\frac{\left(\lambda\left(H-L\right)\left(1-\lambda\right)\left(127+4\lambda\right)\left(2+5\lambda\right)\right)+t\left(20750-\lambda\left(14309+652\lambda\right)\right)}{12\left(\lambda-1\right)\left(\lambda\left(95+4\lambda\right)-512\right)}$ | $\begin{matrix} (((H-L)(1-\lambda)\lambda(\lambda(\lambda(213761409572+\lambda(\lambda(32\lambda(\lambda(7537+40\lambda(80+\lambda)) \\ -5025862)-4193432095)-19089212774))-140883448808)-118984151968)) \\ +t(\lambda(15321222294328+\lambda(\lambda(12072655186+\lambda(75222109847-32\lambda(\lambda(1239545 \\ +8\lambda(15752+205\lambda))-102151112)))-6148175618044))-9777424528000))/(3(\lambda-1) \\ (854387017616+\lambda(\lambda(216667089416+\lambda(6230316280+\lambda(16\lambda(2\lambda(28903+8\lambda\\ (391+5\lambda))-5547017)-1895596843)))-949817488736))) \end{matrix}$ |
| $\theta_{A}^{k}$ | $\frac{\left(\lambda\left(H-L\right)\left(1-\lambda\right)\left(\lambda\left(119+4\lambda\right)-110\right)\right)+t\left(2818-\lambda\left(2017+92\lambda\right)\right)}{2\left(\lambda-1\right)\left(\lambda\left(95+4\lambda\right)-512\right)}$ | $\begin{matrix} (4(H-L)(1-\lambda)\lambda(\lambda(119+4\lambda)-110)(\lambda(169584228+\lambda(4\lambda(\lambda(7173+8\lambda(123+2\lambda)) \\ -411802)-14744913))-189964676)-4t(764195571128+\lambda(\lambda(466526148878+\lambda\\ (6291464287+\lambda(4\lambda(4\lambda(139427+4\lambda(4183+56\lambda))-57485015)-4922881933))) \\ -1193529117260)))/((\lambda-1)(854387017616+\lambda(\lambda(216667089416+\lambda(6230316280+ \\ \lambda(16\lambda(2\lambda(28903+8\lambda(391+5\lambda))-5547017)-1895596843)))-949817488736))) \end{matrix}$ |
| $\theta_{B}^{k}$ | $\frac{\left(\lambda\left(H-L\right)\left(1-\lambda\right)\left(\lambda\left(119+4\lambda\right)-110\right)\right)+t\left(2818-\lambda\left(2017+92\lambda\right)\right)}{2\left(\lambda-1\right)\left(\lambda\left(95+4\lambda\right)-512\right)}$ | $\begin{matrix} (2(H-L)(1-\lambda)\lambda(\lambda(95+4\lambda)-512)(\lambda(119+4\lambda)-110)(784516+\lambda(\lambda(8\lambda(47+2\lambda) \\ -28479)-575396))+2t(\lambda(2097587265080+\lambda(\lambda(3618760874+\lambda(10676407123-16\lambda\\ (2\lambda(178303+232\lambda(77+\lambda))-28772369)))-854674442468))-1322486447456))/ \\ ((\lambda-1)(854387017616+\lambda(\lambda(216667089416+\lambda(6230316280+\lambda(16\lambda(2\lambda(28903+8\lambda\\ (391+5\lambda))-5547017)-1895596843)))-949817488736))) \end{matrix}$ |
| $e_{A}^{k}$ | $\frac{3t\left(598-7\lambda\left(87+4\lambda\right)\right)-3\lambda\left(H-L\right)\left(\lambda-1\right)\left(\lambda\left(103+4\lambda\right)-378\right)}{4\left(\lambda-1\right)\left(\lambda\left(95+4\lambda\right)-512\right)}$ | $\begin{matrix} (((H-L)(1-\lambda)\lambda(467907506528+\lambda(\lambda(147827416844+\lambda(1785266974 \\ +\lambda(8\lambda(8\lambda(35429+2\lambda(869+10\lambda))-4614529)-1306174489)))-554396660216)) \\ +t(1194023520448+\lambda(\lambda(881839729108+\lambda(19611283226+\lambda(8\lambda(16\lambda(25487+ \\ \lambda(3643+50\lambda))-52182263)-8437427009)))-2075352184552)))/((\lambda-1) \\ (854387017616+\lambda(\lambda(216667089416+\lambda(6230316280+\lambda(16\lambda(2\lambda(28903+ \\ 8\lambda(391+5\lambda))-5547017)-1895596843))))-949817488736))) \end{matrix}$ |
| $e_{B}^{k}$ | $\frac{3t\left(598-7\lambda\left(87+4\lambda\right)\right)-3\lambda\left(H-L\right)\left(\lambda-1\right)\left(\lambda\left(103+4\lambda\right)-378\right)}{4\left(\lambda-1\right)\left(\lambda\left(95+4\lambda\right)-512\right)}$ | $\begin{matrix} (3(H-L)(1-\lambda)\lambda(157485226976+\lambda(\lambda(52436212756+\lambda(102349714 \\ +\lambda(32\lambda(\lambda(8\lambda(72+\lambda)-1907)-942314)-620917491)))-188781479112)) \\ +3t(\lambda(486491942104+\lambda(\lambda(5625810602+\lambda(3396382379-32\lambda(\lambda(62293 \\ +8\lambda(704+9\lambda))-4312788)))-229739973900))-267489017216))/((\lambda-1) \\ (854387017616+\lambda(\lambda(216667089416+\lambda(6230316280+\lambda(16\lambda(2\lambda(28903 \\ +8\lambda(391+5\lambda))-5547017)-1895596843)))-949817488736))) \end{matrix}$ |

(Appendix, Page16-18, Line:327-332)

**Reviewers' comments:***7. Why are Fig. 4(a) and (b) plotted in two parts? It would be preferable to plot both scenarios together to compare their variations. Similar comments apply to Figs. 5(a-d).*

**The authors’ Answer:** We sincerely thank the reviewer for this constructive suggestion.

We fully agree that a unified presentation would facilitate direct comparison and avoid unnecessary fragmentation of the data. In the revised manuscript, we have added Fig. 4(a) and Fig. 4(b) combined into a single figure. We use different line styles to distinguish the platform operating model and different colors to distinguish different levels of quality perception differences, and maintain consistency throughout the paper. In the same way, we redraw Figure 5 using the same method. We plot Fig. 5(a) and (c) together to observe the impact of consumer quality perception differences on the leader platform's profit during sequential platform decision-making, and we plot Fig. 5(b) and (d) together to examine the influence of consumer quality perception differences on the follower platform's profit under the same conditions. The redrawn Figures are below and have been updated in the manuscript.

**The revised content is as follows:** We analyze the impact of consumers' quality perception differences on platform profit when platforms make simultaneous and sequential decisions. Assign values to the unit search cost of online shopping $(t)$ and the difference in consumers' quality perception of products $(\Delta s=H-L)$, set the relevant parameters $t=1$, and make$\Delta s=0.1$,$\Delta s=5$, and$\Delta s=10$, respectively, to represent the low, medium, and high levels of the difference in product quality perception[24,53,71]. The probability of consumers obtaining high-quality perception$\lambda\in(0,1)$, draw the profit curves of commission-based platforms and self-operated platforms in different cases. We obtain Fig 4 (a), Fig 5(a) and Fig 5(b).

**Fig 4. Impact of differences in consumer quality perception on platform profit when platforms make simultaneous decisions.** (“─”indicates the platform profit in the self-managed model, “**┄”** indicates the platform profit in the commission-based model)

**Fig 5. Impact of differences in consumer quality perception on platform profit when platforms make sequential decisions.** (“─”indicates the platform profit in the self-managed model, “**┄”** indicates the platform profit in the commission-based model)

As illustrated in Fig 4, the above figure shows the impact of consumer quality perception differences on platform profit when two platforms make decisions at the same time. The dashed line represents the profit curve of commission-based platforms, while the solid line represents the profit curve of self-operated platforms. The three different colors correspond to low, medium, and high levels of perceived quality differences. The figure above summarizes the profit levels of platform $E_{A}$ and platform $E_{B}$ under four combinations of operational strategies. Theoretically, the four strategies correspond to eight profit curves across two platforms. However, in the “simultaneous decision-making” strategy combination, the optimal response functions of the two platforms are symmetrical, resulting in a unique and identical Nash equilibrium solution. The result is that the profit curves of platform $E_{A}$ and platform $E_{B}$ are completely overlapping. Therefore, only six distinguishable profit curves are actually plotted in the figure. Fig 4 shows the profits of self-operated platforms and commission-based platforms under three different quality perception values. From the figure, it can be seen that platform profit increases with the increase of consumer quality perception difference regardless of whether the platform chooses a self-managed model or a commission model.

As illustrated in Fig 5, the above figure shows the impact of consumer quality perception differences on platform profit when two platforms make decisions successively. Fig 5(a) represents the profits of self-operated leader platforms and commission-based leader platforms under three different values of perceived quality differences. Fig 5(b) represents the profits of self-operated follower platforms and commission-based follower platforms under three values of perceived quality difference. As can be seen from the figure, leader platform profit and follower platform profit increase with the increase of consumer quality perception difference regardless of the platform's choice of self-managed model or commission-based model.

(Page33-36, Line:628-666)

Once again, we are very grateful for the insightful feedback that has helped us enhance the quality of our work.

**Reviewers' comments:***8.* *The placement of figures and titles can be confusing for readers. Provide both the title and the figure in the same place, either within the text or at the end of the text.*

**The authors’ Answer:** Thank you very much for your careful review and valuable feedback on our manuscript in your busy schedule. You pointed out that “The placement of figures and titles can be confusing for readers. Provide both the title and the figure in the same place, either within the text or at the end of the text.” We deeply agree with this point and sincerely appreciate your concern about the readability of our content.

We take full responsibility for this issue and sincerely apologize for it. In the initial submission, we didn't closely correspond the titles of the figures to the figures. As a result, readers need to go back and forth to understand the content of the illustrations. This oversight stemmed from our lack of experience in finalizing and submitting figures, and was entirely the fault of our team. In response to your suggestions, we have made adjustments in the revised manuscript. We provide titles and figures in the same location, and in the main text, each figure appears directly after the paragraph in which it is first referenced. In addition, we modified the font size in the table to make the paper more aesthetically pleasing.

Thank you again for pointing out this detail. It has not only significantly improved the readability of our paper, but also provided us with important guidance for our future writing and formatting. We have marked all relevant changes in red in the revised manuscript. We would appreciate your continued feedback.

**Reviewers' comments:***9. The authors' major conclusions highlight the study's important findings. However, those require a more sophisticated presentation.*

**The authors’ Answer:** We sincerely appreciate your valuable feedback. You pointed out that “The authors' major conclusions highlight the study's important findings. However, those require a more sophisticated presentation.” We fully agree with this assessment and sincerely accept your criticism. In response to this comment, we have made the following improvements in the revised manuscript, which are marked in red in the revision notes for your reference:

On the one hand, we have added Tables 3 and 4 to Appendix S15, which systematically list the equilibrium solutions under the four operational strategy combinations, making it easier to draw conclusions intuitively.

**The revised content is as follows:**

**Equilibrium solutions**

*Appendix S15. Summary of equilibrium solutions*

**Table 3.** The game equilibrium solution of self-operated platforms

| 变量 | S-R | D-R |
| --- | --- | --- |
| $a_{A,g}^{k}$ | $\frac{2}{3}\left((H-L)\lambda+\frac{t\left(90-\lambda\left(67+7\lambda\right)\right)}{\left(\lambda-1\right)\left(2\lambda-13\right)}\right)$ |  |
| $a_{B,g}^{k}$ | $\frac{2}{3}\left((H-L)\lambda+\frac{t\left(90-\lambda\left(67+7\lambda\right)\right)}{\left(\lambda-1\right)\left(2\lambda-13\right)}\right)$ | $\frac{11147t-\frac{11558t}{\lambda-1}+71\lambda\left(59H-59L+2t\right)+\frac{142\left(17t+\lambda\left(L-H+4t\right)\right)}{\lambda\left(3+\lambda\right)-5}-\frac{24\left(497\lambda\left(L-H\right)+4t\left(18446+4589\lambda\right)\right)}{4\lambda\left(5+\lambda\right)-95}}{6816}$ |
| $b_{A,g}^{k}$ | $\frac{1}{3}\left[\frac{t\left(128-\lambda\left(61+24\lambda\right)\right)}{\left(\lambda-1\right)\left(2\lambda-13\right)}-(H-L)\lambda\right]$ | $\begin{matrix} \frac{\left(\text{λ}\left(\text{H-L}\right)\left(\text{1-λ}\right)\left(\text{11145+λ}\left(\text{4λ}\left(\text{3λ}\left(\text{67+8λ}\right)\text{-292}\right)\text{-9379}\right)\right)\right)}{48\left(\lambda-1\right)\left(\lambda\left(3+\lambda\right)-5\right)\left(4\lambda\left(5+\lambda\right)-95\right)}+ \\ \frac{t\left(\lambda\left(109188+\lambda\left(3293+2\lambda\left(2\lambda\left(18\lambda-653\right)-9485\right)\right)\right)-98215\right)}{48\left(\lambda-1\right)\left(\lambda\left(3+\lambda\right)-5\right)\left(4\lambda\left(5+\lambda\right)-95\right)} \end{matrix}$ |
| $b_{B,g}^{k}$ | $\frac{1}{3}\left[\frac{t\left(128-\lambda\left(61+24\lambda\right)\right)}{\left(\lambda-1\right)\left(2\lambda-13\right)}-(H-L)\lambda\right]$ |  |
| $c_{A,g}^{k}$ |  |  |
| $c_{B,g}^{k}$ |  |  |
| $\omega_{A}^{k}$ | $\frac{t\left(34-\lambda\left(19+8\lambda\right)\right)}{\left(\lambda-1\right)\left(2\lambda-13\right)}$ | $\frac{\left(\lambda\left(H-L\right)\left(1-\lambda\right)\left(3+2\lambda\right)\right)+t\left(899+\lambda\left(4\left(\lambda-41\right)\lambda-545\right)\right)}{3\left(\lambda-1\right)\left(4\lambda\left(5+\lambda\right)-95\right)}$ |
| $\omega_{B}^{k}$ | $\frac{t\left(34-\lambda\left(19+8\lambda\right)\right)}{\left(\lambda-1\right)\left(2\lambda-13\right)}$ | $\frac{1021t+\lambda\left(H-L\right)\left(\lambda-1\right)\left(3+2\lambda\right)-t\lambda\left(607+4\lambda\left(55+\lambda\right)\right)}{3\left(\lambda-1\right)\left(4\lambda\left(5+\lambda\right)-95\right)}$ |

**Table 4.** The game equilibrium solution of commission-based platforms

| 变量 | S-C | D-C |
| --- | --- | --- |
| $a_{A,g}^{k}$ | $\frac{2\lambda\left(H-L\right)\left(1-\lambda\right)\left(292+\lambda\left(143+4\lambda\right)\right)+t\left(17416+\lambda\left(+\lambda\left(629+44\lambda\right)-14840\right)\right)}{6\left(\lambda-1\right)\left(\lambda\left(95+4\lambda\right)-512\right)}$ | $\begin{matrix} (((H-L)(1-\lambda)\lambda(\lambda(531431374384+\lambda(278396126768+\lambda(\lambda(4\lambda(4\lambda(418961 \\ +4\lambda(5041+56\lambda))-38673485)-6733603987)-47198747420)))-1022023303312)) \\ +t(-33150803072688+\lambda(56507026168288+\lambda(\lambda(2255244859288+\lambda\text{（}255864960371 \\ +\lambda\text{（}32\lambda\text{（}\lambda\text{（}3359+40\lambda\text{（}757+11\lambda))-33429575)-12444370925)))-27188422935136)) \\ -33150803072688))/(6(\lambda-1)(854387017616+\lambda(\lambda(216667089416+\lambda(6230316280+ \\ \lambda(16\lambda(2\lambda(28903+8\lambda(391+5\lambda))-5547017)-1895596843)))-949817488736))) \end{matrix}$ |
| $a_{B,g}^{k}$ | $\frac{2\lambda\left(H-L\right)\left(1-\lambda\right)\left(292+\lambda\left(143+4\lambda\right)\right)+t\left(17416+\lambda\left(+\lambda\left(629+44\lambda\right)-14840\right)\right)}{6\left(\lambda-1\right)\left(\lambda\left(95+4\lambda\right)-512\right)}$ | $\begin{matrix} (((H-L)(1-\lambda)\lambda(\lambda(533060839504+\lambda(270438966704+\lambda(\lambda(4\lambda(4\lambda(509303 \\ +4\lambda(5311+56\lambda))-27078659)-6057290545)-43930520324)))-1019906049712)) \\ +t(\lambda(56839435942528+\lambda(\lambda(2218476834004+\lambda(255051057845+\lambda(32\lambda(\lambda(3899 \\ +40\lambda(757+11\lambda))-33364964)-12404896709)))-27511199806624))-3296852678 \\ 5488))/(6(\lambda-1)(854387017616+\lambda(\lambda(216667089416+\lambda(6230316280+\lambda(16\lambda(2\lambda\\ (28903+8\lambda(391+5\lambda))-5547017)-1895596843)))-949817488736))) \end{matrix}$ |
| $b_{A,g}^{k}$ | $\frac{\lambda\left(H-L\right)\left(\lambda-1\right)\left(\lambda\left(785+28\lambda\right)-1574\right)+5t\left(\lambda\left(2207+100\lambda\right)-3842\right)}{12\left(1-\lambda\right)\left(\lambda\left(95+4\lambda\right)-512\right)}$ | $\begin{matrix} (\left(H-L\right)\left(\lambda-1\right)\lambda(638781615208+\lambda(\lambda\text{ }(318882388426+\lambda(\lambda(16\lambda(2\lambda(212267+ \\ 8\lambda(1160+13\lambda))-4238899)-3383206091)-5115392899))-891241448548))+t(955 \\ 1765639720+\lambda(\lambda(5269008760574+\lambda(86785414771+8\lambda(8\lambda(4\lambda(93785+\lambda(11365+ \\ 152\lambda))-39407831)-6698702351)))-14226916556060)))/(3\left(1-\lambda\right)(854387017616+ \\ \lambda(\lambda\text{ }(216667089416+\lambda(6230316280+\lambda(16\lambda(2\lambda(28903+8\lambda(391+5\lambda))-5547017- \\ 1895596843)))-949817488736))) \end{matrix}$ |
| $b_{B,g}^{k}$ | $\frac{\lambda\left(H-L\right)\left(\lambda-1\right)\left(\lambda\left(785+28\lambda\right)-1574\right)+5t\left(\lambda\left(2207+100\lambda\right)-3842\right)}{12\left(1-\lambda\right)\left(\lambda\left(95+4\lambda\right)-512\right)}$ | $\begin{matrix} (((H-L)(1-\lambda)\lambda(645486251608+\lambda(\lambda(340129529470+\lambda(\lambda(8\lambda(8\lambda(14\lambda(151+2\lambda) \\ -1097)-26757551)-4879041347)-9344291641))-913194084268)))+t(\lambda(129453983 \\ 85620+\lambda(\lambda(10812994871-8\lambda(\lambda(4\lambda(982885+4\lambda(24425+316\lambda))-315356287)-7312 \\ 402544))-4752960387626))-8974557396920))/(3(\lambda-1)(854387017616+\lambda(\lambda(21666 \\ 7089416+\lambda(6230316280+\lambda(16\lambda(2\lambda(28903+8\lambda(391+5\lambda))-5547017)-1895596843))) \\ -949817488736))) \end{matrix}$ |
| $c_{A,g}^{k}$ | $\frac{\left(\lambda\left(H-L\right)\left(1-\lambda\right)\left(127+4\lambda\right)\left(2+5\lambda\right)\right)+t\left(20750-\lambda\left(14309+652\lambda\right)\right)}{12\left(\lambda-1\right)\left(\lambda\left(95+4\lambda\right)-512\right)}$ |  |
| $c_{B,g}^{k}$ | $\frac{\left(\lambda\left(H-L\right)\left(1-\lambda\right)\left(127+4\lambda\right)\left(2+5\lambda\right)\right)+t\left(20750-\lambda\left(14309+652\lambda\right)\right)}{12\left(\lambda-1\right)\left(\lambda\left(95+4\lambda\right)-512\right)}$ | $\begin{matrix} (((H-L)(1-\lambda)\lambda(\lambda(\lambda(213761409572+\lambda(\lambda(32\lambda(\lambda(7537+40\lambda(80+\lambda)) \\ -5025862)-4193432095)-19089212774))-140883448808)-118984151968)) \\ +t(\lambda(15321222294328+\lambda(\lambda(12072655186+\lambda(75222109847-32\lambda(\lambda(1239545 \\ +8\lambda(15752+205\lambda))-102151112)))-6148175618044))-9777424528000))/(3(\lambda-1) \\ (854387017616+\lambda(\lambda(216667089416+\lambda(6230316280+\lambda(16\lambda(2\lambda(28903+8\lambda\\ (391+5\lambda))-5547017)-1895596843)))-949817488736))) \end{matrix}$ |
| $\theta_{A}^{k}$ | $\frac{\left(\lambda\left(H-L\right)\left(1-\lambda\right)\left(\lambda\left(119+4\lambda\right)-110\right)\right)+t\left(2818-\lambda\left(2017+92\lambda\right)\right)}{2\left(\lambda-1\right)\left(\lambda\left(95+4\lambda\right)-512\right)}$ | $\begin{matrix} (4(H-L)(1-\lambda)\lambda(\lambda(119+4\lambda)-110)(\lambda(169584228+\lambda(4\lambda(\lambda(7173+8\lambda(123+2\lambda)) \\ -411802)-14744913))-189964676)-4t(764195571128+\lambda(\lambda(466526148878+\lambda\\ (6291464287+\lambda(4\lambda(4\lambda(139427+4\lambda(4183+56\lambda))-57485015)-4922881933))) \\ -1193529117260)))/((\lambda-1)(854387017616+\lambda(\lambda(216667089416+\lambda(6230316280+ \\ \lambda(16\lambda(2\lambda(28903+8\lambda(391+5\lambda))-5547017)-1895596843)))-949817488736))) \end{matrix}$ |
| $\theta_{B}^{k}$ | $\frac{\left(\lambda\left(H-L\right)\left(1-\lambda\right)\left(\lambda\left(119+4\lambda\right)-110\right)\right)+t\left(2818-\lambda\left(2017+92\lambda\right)\right)}{2\left(\lambda-1\right)\left(\lambda\left(95+4\lambda\right)-512\right)}$ | $\begin{matrix} (2(H-L)(1-\lambda)\lambda(\lambda(95+4\lambda)-512)(\lambda(119+4\lambda)-110)(784516+\lambda(\lambda(8\lambda(47+2\lambda) \\ -28479)-575396))+2t(\lambda(2097587265080+\lambda(\lambda(3618760874+\lambda(10676407123-16\lambda\\ (2\lambda(178303+232\lambda(77+\lambda))-28772369)))-854674442468))-1322486447456))/ \\ ((\lambda-1)(854387017616+\lambda(\lambda(216667089416+\lambda(6230316280+\lambda(16\lambda(2\lambda(28903+8\lambda\\ (391+5\lambda))-5547017)-1895596843)))-949817488736))) \end{matrix}$ |
| $e_{A}^{k}$ | $\frac{3t\left(598-7\lambda\left(87+4\lambda\right)\right)-3\lambda\left(H-L\right)\left(\lambda-1\right)\left(\lambda\left(103+4\lambda\right)-378\right)}{4\left(\lambda-1\right)\left(\lambda\left(95+4\lambda\right)-512\right)}$ | $\begin{matrix} (((H-L)(1-\lambda)\lambda(467907506528+\lambda(\lambda(147827416844+\lambda(1785266974 \\ +\lambda(8\lambda(8\lambda(35429+2\lambda(869+10\lambda))-4614529)-1306174489)))-554396660216)) \\ +t(1194023520448+\lambda(\lambda(881839729108+\lambda(19611283226+\lambda(8\lambda(16\lambda(25487+ \\ \lambda(3643+50\lambda))-52182263)-8437427009)))-2075352184552)))/((\lambda-1) \\ (854387017616+\lambda(\lambda(216667089416+\lambda(6230316280+\lambda(16\lambda(2\lambda(28903+ \\ 8\lambda(391+5\lambda))-5547017)-1895596843))))-949817488736))) \end{matrix}$ |
| $e_{B}^{k}$ | $\frac{3t\left(598-7\lambda\left(87+4\lambda\right)\right)-3\lambda\left(H-L\right)\left(\lambda-1\right)\left(\lambda\left(103+4\lambda\right)-378\right)}{4\left(\lambda-1\right)\left(\lambda\left(95+4\lambda\right)-512\right)}$ | $\begin{matrix} (3(H-L)(1-\lambda)\lambda(157485226976+\lambda(\lambda(52436212756+\lambda(102349714 \\ +\lambda(32\lambda(\lambda(8\lambda(72+\lambda)-1907)-942314)-620917491)))-188781479112)) \\ +3t(\lambda(486491942104+\lambda(\lambda(5625810602+\lambda(3396382379-32\lambda(\lambda(62293 \\ +8\lambda(704+9\lambda))-4312788)))-229739973900))-267489017216))/((\lambda-1) \\ (854387017616+\lambda(\lambda(216667089416+\lambda(6230316280+\lambda(16\lambda(2\lambda(28903 \\ +8\lambda(391+5\lambda))-5547017)-1895596843)))-949817488736))) \end{matrix}$ |

(Appendix, Page16-18, Line:327-332)

On the other hand, we strengthened the textual description, supplemented the relevant conclusions, and highlighted the important findings of this paper.

**The revised content is as follows:**

**7. Conclusions**

Different from previous literature that focused on objective quality improvement or static price discrimination [28,31], this paper combines a two-period model to make consumer quality perception differences an endogenous core variable in two-period dynamic games, while also considering the interaction between platform operating models (self-operated/commission-based) and decision-making sequences (simultaneous/sequential). We respectively construct dynamic pricing game models under commission-based and self-operated models, and conduct a comparative analysis of decision variables such as product prices, commission rates, and discount amounts in four cases: self-operated model under simultaneous decision-making by both platforms (S-R), commission-based model under simultaneous decision-making by both platforms (S-C), self-operated model under sequential decision-making by both platforms (D-R), and commission-based model under sequential decision-making by both platforms (D-C). We explore the impact of consumer product quality perception differences and channel competition on supply chain equilibrium decision-making, and explore the impact of the probability change of consumers' high-quality perception on platform profit, manufacturers' profit and platform operation mode choices through numerical simulation, so as to provide a theoretical basis for platforms to formulate effective operation strategies under different market factors.

The main conclusions of this study are as follows: 1) Both in the commission-based (prices are decided by the manufacturers) model and the proprietary (prices are decided by the platforms) model, when the probability of consumers obtaining high-quality perception is high, the manufacturing enterprises and the platforms can increase the market price to obtain higher profit. 2) In the commission-based model, platforms can attract consumers by raising the commission rate and offering greater price concessions, thereby enhancing their profit and market competitiveness. 3) After locking in consumers with low prices in the first cycle, the leader platform can maintain its market share in the second cycle even if it raises the price for repeat purchases, thanks to its perceived advantage. This explains the sustainability of the “subsidize first, raise prices later” strategy in reality. 4)The widening perception gap has a positive effect on the profits of both platforms and manufacturers, and this conclusion holds true in both self-operated and commission-based models, indicating that differentiated experiences have become a new profit lever. 5) When platforms compete simultaneously, higher profit can be achieved by adopting a self-managed model. In sequential decision-making scenarios, the leader platform must consider both product quality differences and the probability of consumers perceiving high quality in selecting an operating model. Follower platforms can achieve higher profit when the leader platform prefers the commission-based model.

This study has important management implications for e-commerce platforms and manufacturers. Self-operated platforms should continue to invest in quality traceability systems and brand storytelling to amplify consumers' perception of high quality, thereby supporting subsequent price increases. Commission-based platforms can use high commission income to subsidize new users, attract low-perception migrants with coupons, and encourage manufacturers to disclose quality signals, creating a virtuous cycle. For manufacturers, when working with commission-based platforms, they can enhance consumer perception by strengthening digital quality control, thereby gaining higher commission tolerance and pricing flexibility from the platform. When working with self-operated platforms, they should focus on ensuring stable supply so that the platform can implement dynamic high-price strategies. This study is not only applicable to e-commerce platforms but also has significant implications for other industries such as retail and services that face multi-channel competition and consumer strategic behavior. Among these, the 3C electronics, beauty, and luxury goods categories stand out for their rapid iteration. In these industries, consumers have a fast-paced perception of updates and high price elasticity, and adopting the strategies outlined in this paper can quickly amplify profits.

(Page46-48, Line:826-874)

In addition, based on your suggestion, we have made a more complex demonstration in the 6.3 numerical simulation section, integrating the changes in platform profits under the four strategies into a single graph to intuitively show the differences in profits under each context and their significance.

**The revised content is as follows:**

**Fig 11. Platform operation mode selection.** (“─”indicates the platform profit in the self-managed model, “**┄”** indicates the platform profit in the commission-based model)

(Page43, Line:745-747)

Figures 11(a), 11(b) and 11(c) represent the platform profits under the four operational strategies when the perceived difference in product quality is low, the perceived difference in product quality is medium and the perceived difference in product quality is high, respectively. In this case, the solid line indicates the platform profit in the self-managed model and the dashed line indicates the platform profit in the commission-based model, with different colors to distinguish the platform decision order. The figure above summarizes the profit levels of platform $E_{A}$ and platform $E_{B}$ under four combinations of operational strategies. Theoretically, the four strategies correspond to eight profit curves across two platforms. However, in the “simultaneous decision-making” strategy combination, the optimal response functions of the two platforms are symmetrical, resulting in a unique and identical Nash equilibrium solution. The result is that the profit curves of platform $E_{A}$ and platform $E_{B}$ are completely overlapping. Therefore, only six distinguishable profit curves are actually plotted in the figure. As can be seen from the figure, regardless of whether the platform adopts the self-operating mode or the commission-based mode, the platform profit under simultaneous decision-making is lower than that under sequential decision-making. This is because when making simultaneous decisions, sellers and platforms are unable to observe each other's final strategies and can only make one-off pricing or commission settings through anticipation, leading to a tendency for both sides to adopt more aggressive low-price or low-commission strategies in order to capture demand and compress profit margins. Under sequential decision-making, the later actors can optimize their strategies based on the strategies of the first actors to achieve complementary strategies. It can be seen that the e-commerce platform, while choosing the optimal operation strategy in conjunction with the market environment, can enhance its market positioning by clarifying its strategic positioning and differentiation advantages, branding and trust system construction, and expanding its innovative business model, thus reducing risks such as demand forecasting errors and enhancing the platform's profits.

(Page45-46, Line:800-824)

We are deeply aware that the way conclusions are presented directly affects readers' judgment of the value of research. If there are still any shortcomings in this revision, we kindly ask for your continued criticism and guidance. Once again, we sincerely thank you for your patience and constructive feedback on our work!

**Reviewers' comments:***10. Add management implications in the conclusion section.*

**The authors’ Answer:** Thank you for your valuable comments! We have added management insights after the conclusion summary, clarifying the guidance role of the research conclusions for decision-making by e-commerce platforms and manufacturers, and supplementing the practical significance of this research for the industry.

**The revised content is as follows:** This study has important management implications for e-commerce platforms and manufacturers. Self-operated platforms should continue to invest in quality traceability systems and brand storytelling to amplify consumers' perception of high quality, thereby supporting subsequent price increases. Commission-based platforms can use high commission income to subsidize new users, attract low-perception migrants with coupons, and encourage manufacturers to disclose quality signals, creating a virtuous cycle. For manufacturers, when working with commission-based platforms, they can enhance consumer perception by strengthening digital quality control, thereby gaining higher commission tolerance and pricing flexibility from the platform. When working with self-operated platforms, they should focus on ensuring stable supply so that the platform can implement dynamic high-price strategies. This study is not only applicable to e-commerce platforms but also has significant implications for other industries such as retail and services that face multi-channel competition and consumer strategic behavior. Among these, the 3C electronics, beauty, and luxury goods categories stand out for their rapid iteration. In these industries, consumers have a fast-paced perception of updates and high price elasticity, and adopting the strategies outlined in this paper can quickly amplify profits.

(Page47-48, Line:860-874)

**Reviewers' comments:***11. Provide references to elaborate on the study's limitations and future directions.*

**The authors’ Answer:** Thank you for your valuable feedback!

In order to further highlight the research conclusions and their guidance for decision-making by e-commerce platforms and manufacturers, we have revised the conclusions section. We have supplemented the practical significance of this study for the industry. Furthermore, we have combined existing research to further discuss the limitations of this study, providing a reference for future expansion studies.

**The revised content is as follows:** This study has important management implications for e-commerce platforms and manufacturers. Self-operated platforms should continue to invest in quality traceability systems and brand storytelling to amplify consumers' perception of high quality, thereby supporting subsequent price increases. Commission-based platforms can use high commission income to subsidize new users, attract low-perception migrants with coupons, and encourage manufacturers to disclose quality signals, creating a virtuous cycle. For manufacturers, when working with commission-based platforms, they can enhance consumer perception by strengthening digital quality control, thereby gaining higher commission tolerance and pricing flexibility from the platform. When working with self-operated platforms, they should focus on ensuring stable supply so that the platform can implement dynamic high-price strategies. This study is not only applicable to e-commerce platforms but also has significant implications for other industries such as retail and services that face multi-channel competition and consumer strategic behavior. Among these, the 3C electronics, beauty, and luxury goods categories stand out for their rapid iteration. In these industries, consumers have a fast-paced perception of updates and high price elasticity, and adopting the strategies outlined in this paper can quickly amplify profits.

We examine the impact of price factors on channel competition within the context of variations in consumer perception of product quality. However, it is essential to acknowledge that buyer feedback [72] and return rates [73] also play significant roles in consumers' decisions regarding purchasing channels. Consequently, future research endeavors will delve deeper into online reviews and return policies to more precisely forecast market responses. Such research will comprehensively encapsulate the multifaceted factors influencing consumer perception of product quality, thereby affording enterprises more profound market insights and strategic directives.

(Page47-48, Line:860-882)

Thank you again for your patience and guidance on our manuscript! If there are still any inadequacies, please continue to criticize and correct them, and we will do our best to continue to modify them.

**Reviewers' comments:***12. The figures lack emphasis and clarity. Please redo these in a flawless manner.*

**The authors’ Answer:** Thank you very much for pointing out the key issue of "the figures lack emphasis and clarity". Your comments have made us realize that, as the "second language" of the thesis, the quality of graphs and charts has a direct impact on the readers' understanding of and trust in the research results. We would like to extend our sincerest apologies for any previous dyslexia caused by our omission. In response to your suggestion, we have systematically processed all the images into vector form. All updated images will be submitted with the response letter.

In addition, we have adjusted the font in Table 1 to make the table more aesthetically pleasing.

**Table 1.** The summary of literature and factors addressed by our study

| Reference | two-period | consumer preferences | price discrimination | quality perception | platform operations model | channel competition | e-commerce platform |
| --- | --- | --- | --- | --- | --- | --- | --- |
| Zhao and Wang [3] |  | √ |  |  | √ | √ | √ |
| Wang and Yan [10] |  | √ |  |  | √ | √ | √ |
| Sun et al [14] |  | √ |  |  |  | √ |  |
| Zhang and Wen [67] |  |  |  |  | √ | √ | √ |
| K Matsui [65] |  |  |  |  | √ | √ | √ |
| Reimers et al [20] | √ | √ | √ |  |  | √ |  |
| Liu and Zhang [24] | √ | √ | √ |  |  | √ |  |
| Meng et al [47] |  | √ |  | √ |  | √ | √ |
| Ma et al [49] | √ | √ | √ |  | √ | √ | √ |
| Zhang et al [63] | √ |  | √ |  | √ | √ | √ |
| Fudenberg et al [27] | √ | √ | √ |  |  | √ |  |
| Our study | √ | √ | √ | √ | √ | √ | √ |

(Page10, Line:219-220)

Thank you again for your patience and guidance on our manuscript! If there are still any inadequacies, please continue to criticize and correct them, and we will do our best to continue to modify them.

**Reviewer #2:**

*This study examines a product retail supply chain consisting of two e-commerce platforms, two manufacturers, and strategic consumers. The authors construct a two-period dynamic pricing game model to compare commission-based and self-operated models for the platforms. Based on different operating modes and decision sequences, four scenarios are considered. The research is relatively comprehensive and carries both theoretical significance and practical relevance. However, several key issues remain to be addressed. Therefore, a “Minor Revision” is recommended. The specific suggestions are as follows:*

**Response:** Thank you for the comments and suggestions on the manuscript. We have carefully revised the manuscript and provided the point-by-point response below.

**Reviewers' comments:** *1. The statement “we provide a new theoretical framework for…” does not sufficiently demonstrate how the study fills the theoretical gap. The authors should clearly articulate the innovations and contributions in the Introduction to enhance the paper’s theoretical value.*

**The authors’ Answer:** Thank you very much for your valuable feedback. We have revised the introduction according to your suggestions, pointing out the shortcomings of existing research, clearly stating the innovations and contributions of this paper, and clarifying the theoretical value of the paper.

**The revised content is as follows:** Currently, research on two-period pricing typically assumes that consumer valuations are fixed or that price is the only motivation for transfer [24,25]. Although some literature has considered consumers' strategic behavior, few have incorporated “perceived quality updates” into dynamic pricing models. Although product quality perception is widely recognized as a key factor influencing consumer decision-making [26,27], most studies focus on the formation mechanisms of quality perception in static environments (such as online reviews and brand awareness), focusing on product quality itself or price competition [8,9]. For example, improving objective product quality through supply chain optimization, quality certification, or technological upgrades [27,28] while ignoring its impact on platform operational strategies (such as pricing and commission models) in multi-cycle dynamic competition [28,29], and systematic exploration of how differences in consumer quality perception affect platform pricing and model selection at the strategic level remains insufficient.

Although there is a wealth of research on price discrimination and platform operating models [17,30], studies on the choice between platform self-operation and commission models have mainly focused on aspects such as channel power, commission rates, and showroom behavior [31,32]. Few scholars have combined research on consumer quality perception differences with dynamic two-period game analysis, and have not fully explored how consumers' channel switching behavior based on quality perception in the second period affects the platform's optimal pricing and model selection. Most of the current research is based on a single platform or static model [33], and there's a lack of theoretical modeling of dynamic interactions under dual-platform competition. Few scholars have done a systematic analysis of the interaction between quality perception differences and platform decision order.

In response to the shortcomings of existing research, we construct a two-period dynamic game model, which classifies strategic consumers into two types of high and low perception according to the different valuations of consumers' perception of product quality [24,34]. Considering the impact of price factors on channel competition in the context of differences in consumers' perception of product quality, we divide the enterprise strategy portfolio into four types: self-operated model under simultaneous decision-making by both platforms (S-R), commission-based model under simultaneous decision-making by both platforms (S-C), self-operated model under sequential decision-making by both platforms (D-R), and commission-based model under sequential decision-making by both platforms (D-C). Considering the uncertainty factors of product price and consumer product quality perception at different stages, we provide a new theoretical framework for studying the operational decisions of different types of platforms in different periods, which helps platforms and manufacturers to under-stand the market dynamics and consumer behaviors better so that they can flexibly adjust their pricing strategies, commission policies and incentives in actual operations. This paper clearly answers the following research questions: 1) the choice of pricing strategies of manufacturers and platforms under different operating modes. 2) The impact of commission rate and discount amount on decision-making under different decision sequences of enterprises under the same operating mode. 3) The impact of the probability changes of consumer product quality perception difference on the profit of manufacturers and platforms.

(Page4-5, Line:64-104)

Thank you again for your patience and guidance on our manuscript! If there are still any inadequacies, please continue to criticize and correct them, and we will do our best to continue to modify them.

**Reviewers' comments:***2. The literature review section requires appropriate adjustment to strengthen its alignment with the focus of the paper. In particular, the discussion should emphasize the three highly relevant streams of literature: strategic consumers (including purchase intention, quality perception, and price discrimination), operational models of e-commerce platforms, and decision-making sequences. A clearer integration of these themes would improve the coherence and academic contribution of the review.*

**The authors’ Answer:** Thank you for your valuable feedback! We have made appropriate adjustments to the literature review section based on your comments to better reveal the interactive mechanisms between decision-making sequence, quality perception, and operational models, thereby improving the consistency and academic contribution of the review.

**The revised content is as follows:** Consumers' perception of product quality is a key factor influencing their purchasing decisions. In early studies on consumers' two-period buying behavior, scholars argued that consumers' product preferences are fixed and that they switch purchasing channels solely due to price differences; the first purchase conveys the preference, and the only motivation for switching is the lower price [25,35-36]. Fudenberg et al. [25] constructed a two-period game model, proposing that consumers' preferences are completely independent over time. Shin and Sudhir [37] make a modified formulation of preference randomness to adapt it to the nature of correlation over time. Subsequent scholars pointed out that consumer valuations in multi-cycle purchases are uncertain [38], in a two-period purchasing supply chain where there is multi-channel competition, consumers are unable to accurately assess the quality of the product in the first period [39] and obtain the actual value of the product they have purchased in the second period [40], and if consumers realize a low value in the first period, switching purchases in the second period implies a higher expected product value [41]. Numerous studies have shown that product quality perception [6], product prices [8,42], platform live streaming [42,43], and online product reviews [44,45] are the critical factors influencing consumers' purchasing decisions. Among them, consumers' perception of online product quality is a subjective comparison of the quality and value of different products under the combined effect of personal factors and shopping situations, which is between objective and subjective [26] and is influenced by internal and external information of enterprises [46]. Consumers' expected valuation of product quality refers to consumers' prior beliefs about product quality, which can be divided into high prior quality and low prior quality [45]. Consumers' perception of product quality in online shopping is affected by a variety of factors, including product quality [47], service [9], the price [27], product ranking [48], and the degree of knowledge of product information [49,50]. In addition, enterprise image [51], brand perception [28], and online reviews [45] are also important factors affecting consumers' product quality perception. Han et al. [51] found that enterprise ethical attributes have a significant positive impact on the formation of consumers' perceived status. Li et al. [28] explored retailers' optimal own product quality positioning decisions; it was found that retailers would introduce low-quality own products when branded product quality is high. Meng et al. [29] explored the impact of quality perception level on the pricing decisions and profit of supply chain members by establishing an online shopping supply chain that takes into account consumers' quality perception level. Existing research has mostly focused on static environments, with insufficient research on how differences in quality perception under dynamic multi-cycle conditions regulate consumer channel switching behavior.

Strategic consumers dynamically adjust their purchasing channels based on price and perceived product quality. Online retailers have increasingly adopted price discrimination strategies to reward new consumers and incentivize channel-switching behavior in product purchases [24]. Furthermore, consumer segments can be categorized as high-value and low-value groups based on their purchasing patterns [52,53]. Existing research classifies discount strategies into proportional preference and monetary preference [14,54,55]. Coupon distribution can effectively attract new consumers [15], bring stronger consumer engagement [56], increase consumer purchase impulse [57] and promote social empowerment [58], etc. Discount rate and coupon ease of use affect consumer purchase decisions [59]. Zhang et al. [55] empirically demonstrate that proportional discounts are more effective for low-grade products, whereas monetary discounts better suit high-grade products. Zhang et al. [60] established two promotional models, price discount and cash coupon, which are decided by the platform and consider the merchant's advertisement investment strategy, and found that the platform should use the cash coupon strategy when the transaction rate is high, and the price of the products is low. Otherwise, it should use the price discount strategy. By comparing the two scenarios of platform-issued and non-coupon issued, Wang and Yang [61] found that platform-issued coupons are beneficial to the platform and manufacturers' profit but may harm retailers' interests. Chen et al. [62] further reveal that intensified price discounts diminish retailers’ incentives to invest in advertising when online and offline channels compete on both price and promotional efforts. Li et al. [63] discussed the issue of issuing coupons in the online channel under omnichannel retailing. They found that retailers should implement a low-price strategy in the pure online channel to attract consumers with high travelling costs and issue coupons with substantial discounts in the BOPS (order online, pick up offline) channel to attract consumers with a high perceived value of product experience. In addition, Li et al. [64] analyzed the optimal promotional strategies of manufacturers and retailers before and after the introduction of private labels by retailers for an omnichannel supply chain providing BOPS services. The implementation of these promotional strategies can stimulate consumers' purchasing behavior to a certain extent and, at the same time, play a role in pulling new products and promotions [15,65]. These studies mostly assume that consumer valuations are exogenous, ignoring the dynamic impact of differences in quality perceptions on price sensitivity [24]. We combine a two-period model to analyze how consumers' channel switching behavior based on quality perceptions affects product pricing.

The platform operation mode affects the optimal pricing of products to a certain extent [33]. Current research on the choice of platform operation model mainly focuses on the comparison and choice of self-operated model and commission model [30]. The main difference between the two models is that platforms assume different roles in sales [17]. When online and offline channels compete, the commission-based model outperforms the self-operated model. With the intensification of competition between online and offline channels, enterprises will prefer the commission-based model [17]. Consider the case of retail competition between two enterprises that can generate Pareto improvements, one using the self-operated model and the other using the commission-based model [66]. When a supplier does not have a direct sales channel, at least one competing supplier always adopts a commission-based model [67]. In markets with heterogeneous firm competitiveness, the dominant e-retailer should adopt a proprietary model [68]. In addition to channel competition, the commission fee is also an important factor affecting the choice of retailers' operation model [31]. Zhen and Gu [32] considered the showrooming behavior of consumers when purchasing experiential products. They found that when the commission ratio is within a reasonable range, retailers choose the commission-based model if the consumer satisfaction rate of the product is very low, and conversely, they choose the self-operated model. In addition, Wei and Dong [69] derived the conditions under which all four distribution models are in equilibrium and found that the use of pricing power and resale double marginalization plays a key role in the equilibrium outcome. In supply chain games, the order of decision-making significantly affects the equilibrium outcome. Fudenberg et al. [25] pointed out that leaders can obtain higher profits through first-mover advantages, and in e-commerce platform competition, differences in decision-making sequences further interact with operating models. However, existing literature often ignores the joint effect of decision order and quality perception. For example, Zhang Zijian and Liu Wenjing [31] studied pattern selection under channel competition, but did not analyze how differences in consumer quality perception moderate the pricing strategies of leaders and followers. This paper reveals the interactive mechanism between decision-making sequence, quality perception, and operating mode by constructing Stackelberg and Nash equilibrium models.

(Page6-10, Line:126-218)

Once again, thank you for your professional insights and valuable comments. If there are any areas that require further clarification, please do not hesitate to let us know, and we will do our utmost to make timely revisions.

**Reviewers' comments:***3. Figure 1 should be revised, as the differences between the self-operated and commission-based models are minimal. Additionally, since the paper employs the Hotelling model, a brief explanation should be provided to improve clarity and readability.*

**The authors’ Answer:** Thank you for your careful review of our manuscript and for your valuable comments on Figure 1. We are deeply introspective about your corrections to "the differences between the self-operated and commission-based models are minimal" and "a brief explanation should be provided to improve clarity and readability", and we sincerely appreciate your meticulous attention to detail and logic.

In response to the above comments, we have carefully revised them one by one and would like to report as follows:

(1) Due to the minimal differences between the self-operated model and the commission-based model, we have consolidated the original Figures 1(a) and Figures 1(b) into a single illustration, divided into two columns, and added annotations to highlight the main differences between the two models.

(2) For the convenience of readers, we provide a brief explanation of the important assumptions of the Hotelling model and the interpretation of important variables of the paper, which improves the clarity and readability of the text on the basis of ensuring academic rigor. The revised Figure 1 is shown in the following figure and has replaced the original figure.

**Figure 1 Schematic diagram of the operating model**

(Page13, Line:276-277)

Thank you again for your patience and guidance on our manuscript! If there are still any inadequacies, please continue to criticize and correct them, and we will do our best to continue to modify them.

**Reviewers' comments:***4. Several formatting and consistency issues should be corrected, such as inconsistent expressions like “4.1. Self-reliant model” and the presence of Chinese symbols in the formulas of Theorem 5 and Theorem 6.*

**The authors’ Answer:** Thank you very much for your careful review of our manuscript and your valuable comments. With regard to the consistency of the format and symbols that you have pointed out, we have checked each item and revised them carefully, and have unified the expressions of “self-operated model” and “commission-based model” as follows:

**Table 2.** Parameter values of numerical simulations

| **Parameter** | **Definitions** |
| --- | --- |
| $\bar{v}$ | Valuation of consumer expectations of product quality |
| $\lambda$ | Probability that a consumer will receive a high-quality perception$(0<\lambda<1)$ |
| $\text{g}$ | $\text{g}=(C,R)$indicates commission-based and self-operated models, respectively |
| $k$ | $k=(S,D)$indicates simultaneous and sequential decision-making by the two platforms, respectively |
| $tx$ | Negative utility incurred by consumers purchasing products on the platform |
| $y$ | The point at which the utility of the product$A$and the product$B$are indistinguishable to consumers in the first period |
| $x_{0}$ | Point of no difference in utility between consumers repeating and switching purchases of a product$A$in the second period |
| $x_{1}$ | Point of no difference in utility between consumers repeating and switching purchases of a product$B$in the second period |
| $t$ | unit search cost |
| $\omega_{j}^{k}$ | Wholesale prices for Manufacturer$M_{j}$decisions |
| $\theta_{i}^{k}$ | Commission rate charged by the platform$E_{i}$ |
| $\beta^{k}$ | Price discount factor granted by platform$E_{i}$to new consumers |
| $e_{i}^{k}$ | Amount of price concessions granted by platform$E_{i}$to new consumers |
| $d_{j}$ | Market demand for product$A(B)$in the first period |
| $D_{j}$ | Market demand for product$A(B)$in the second period |
| $a_{i,g}^{k}$ | Retail prices of first-period products |
| $b_{i,g}^{k}$ | Repeat purchase price of second-period products |
| $c_{i,g}^{k}$ | Switching purchase price for second period products |
| $\Pi_{i,g}^{k}$ | Total Supply Chain Profit |
| $\pi(M_{j,g}^{k})$ | Second-period profit for manufacturers |
| $\Pi(M_{j,g}^{k})$ | Manufacturer's total profit for the second period |
| $\pi(E_{i,g}^{k})$ | Second-period profit of the platform |
| $\Pi(E_{i,g}^{k})$ | Total two-period profit of the platform |

(Page14-15, Line:280)

**4.1.** **Self-operated model under simultaneous decision making (S-R)**

(Page15, Line:288-289)

**4.3.** **Self-operated model under sequential decision-making (D-R)**

(Page20, Line:368)

The profit of self-operated platform $E_{i}$ in the second period and the total profit in the two periods are as follows:

(Page21, Line:384-385)

**Fig 2. Distribution of f intervals in Self-operated model.**

(Page30, Line:560)

In response to the problem of “Chinese symbols in formulas”, we have scrutinized the whole text and modified the symbol errors as follows:

**Theorem 5.** In the self-managed model, when platforms make decisions sequentially, the following relationship between the retail prices of products of the leader platform and the follower platform can be obtained:

$$\left\{\begin{matrix} a_{AR}^{D}>a_{BR}^{D},b_{AR}^{D}<b_{BR}^{D}\text{, }c_{AR}^{D}>c_{BR}^{D},0<\frac{t}{H-L}<l2 \\ a_{AR}^{D}>a_{BR}^{D},b_{AR}^{D}>b_{BR}^{D},c_{AR}^{D}>c_{BR}^{D},l2<\frac{t}{H-L}<l1 \\ a_{AR}^{D}<a_{BR}^{D},b_{AR}^{D}>b_{BR}^{D},c_{AR}^{D}>c_{BR}^{D},l1<\frac{t}{H-L}<l3 \\ a_{AR}^{D}<a_{BR}^{D},b_{AR}^{D}>b_{BR}^{D},c_{AR}^{D}<c_{BR}^{D},\frac{t}{H-L}>l3>0 \end{matrix}\right.$$

(Page28-29, Line:536-539)

**Theorem 6.** In the commission-based model, the relationship between the amount of price preference given by the platform to new consumers and$\lambda$ can be obtained when the platform makes successive decisions as follows, where$f_{2}>f_{1}$.The values of $f_{1}$ and $f_{2}$ are illustrated in the Appendix S10 .

$$\left\{\begin{matrix} \frac{\partial e_{A}^{D}}{\partial\text{λ}}>0,\frac{\partial e_{B}^{D}}{\partial\text{λ}}>0,\frac{t}{H-L}>f2>0 \\ \frac{\partial e_{A}^{D}}{\partial\text{λ}}>0,\frac{\partial e_{B}^{D}}{\partial\text{λ}}<0,f1<\frac{t}{H-L}<f2 \\ \frac{\partial e_{A}^{D}}{\partial\text{λ}}<0,\frac{\partial e_{B}^{D}}{\partial\text{λ}}<0,0<\frac{t}{H-L}<f1 \end{matrix}\right.$$

(Page31, Line:586-590)

We understand that rigor of detail is at the heart of academic norms, and this omission stems from our negligence; we thank you for correcting us so that we can improve. A revised manuscript with all changes marked has been submitted with the letter of response. If there is still anything that is not covered, we would appreciate further guidance and we will cooperate fully.

**Reviewers' comments:***5. In Section 6: Numerical Simulation, the sources of numerical values are not specified, which reduces the practical relevance of the study. Furthermore, when comparing multiple figures, most results remain unchanged, making the analysis appear somewhat repetitive.*

**The authors’ Answer:** We feel great thanks for your professional review work on our article.

In the numerical experiment part, we assign values to the unit search cost of online shopping $(t)$ and the difference between consumers' perception of product quality$(\Delta s=H-L)$. Ma Dongsheng et al. [53] assume $t=1$ in the Hotelling model, study the impact of service differentiation on the pricing of e-commerce platforms, and compare the equilibrium profits under the self-operated and commission-based models. This paper draws on its approach to assign t to 1 to simplify the model derivation and focus on the core competitive strategy analysis. Xie Guangming et al. [71] conducted an empirical analysis based on data from Amazon China, indicating that the coefficient of variation of word-of-mouth discrepancy for practical attribute products is below 0.2. Liu Weiqi et al. [24] establish $H=6.0$ and $L=1.0\text{ (}\Delta\text{s=5)}$ in the context of pricing discrimination strategies, representing consumer disagreement on the functionality and durability of electronic products (such as headphones). Ma Dongsheng et al. [53] used $\Delta\text{s=8.0}$ in the luxury e-commerce scenario in the service differentiation model to verify the pricing resilience of the leader platform under high differentiation. Therefore, we assign $\Delta\text{s}$to 0.1, 5, and 10, respectively, which represent the three levels of low, medium, and high perceived differences in product quality.

When comparing simulation results, most results are similar due to the limited impact of consumer quality perception differences, platform operating models, decision-making sequences, and other factors on the simulation results. Through a broad comparative system of various situations, we explore how differences in consumer quality perception affect product pricing and platform operation model choices at the strategic level.

The numerical simulation results in this paper provide a theoretical basis for the selection of platform operating models. The study shows that differences in consumer perceptions of product quality and the order of platform decisions are both factors influencing the selection of platform operating models. Regardless of whether the platform adopts a self-operated business model or a commission-based business model, the platform's profits under simultaneous decision-making are lower than those under sequential decision-making. When competing platforms make decisions simultaneously, choosing a self-operated model can yield higher profits; when competing platforms make decisions sequentially, choosing a commission-based model allows follower platforms to earn higher profits.

Once again, thank you for your professional insights and valuable comments. If there are any areas that require further clarification, please do not hesitate to let us know, and we will do our utmost to make timely revisions.

[24] Liu W, Zhang J. Research on price discrimination strategy considering consumers' expected regret. China Manag Sci. 2018;26(5):1-8.

[53] Ma D, Song H, Zhao J, Zhu Y. Research on behavior-based pricing strategies and contractual models for service differentiated e-commerce platforms. China Manag Sci. 2023;31(2):215-225.

[71] Xie G, Qiu D, Jiang Y. A Research on the e-WOM's Dispersive Coefficient of Variation from the Perspective of Endogeneity. Management Review. 2018,30(04):94-10.

All the lines and pages indicated above are in the revised manuscript. Thank you for your comments. In addition, we added that the role of funders in the study was explained.

**Author Contributions**

B.X.: Conceptualization, Methodology, Data Curation, Validation, Writing— original draft. Q.G.: Supervision, Writing—review and editing, formal analysis, Validation. Y.C.: Mathematics Analysis, Software, Visualization. M.N.: Writing – review & editing, Investigation. All authors have read and agreed to the published version of the manuscript.

(Page:48-49, Line:883-887)

**Author's supplement:** In addition to the above changes, we also found some grammatical errors in the manuscript, marked in red to distinguish them, which are shown as follows:

Zhang et al. [55], on the other hand, found through empirical research that low-grade products are more suitable for the use of proportional discounts, and high-grade products are ideal for the use of monetary discounts.

Zhang et al. [55] empirically demonstrate that proportional discounts are more effective for low-grade products, whereas monetary discounts better suit high-grade products. (Page:8, Line:167-169)

In addition, Chen et al. [62] found that increasing price discounts reduce retailers' incentives to invest in advertisements when there is price and advertising competition in both online and offline channels.

Chen et al. [62] further reveal that intensified price discounts diminish retailers’ incentives to invest in advertising when online and offline channels compete on both price and promotional efforts. (Page:8, Line:176-178)

When there is channel competition between online and offline channels, the commission-based model is more effective than the self-operated model.

When online and offline channels compete, the commission-based model outperforms the self-operated model. (Page:9, Line:194-195)

When there is a difference in the competitiveness of enterprises in the market, the dominant e-retailer should choose the proprietary model [68].

In markets with heterogeneous firm competitiveness, the dominant e-retailer should adopt a proprietary model [68]. (Page:9, Line:200-202)

Consequently, manufacturers can elevate the price of repeat purchases to achieve higher profit.

Consequently, manufacturers can raise repeat-purchase prices to extract greater profits. (Page:25, Line:454-455)

These efforts, in turn, positively impact consumers' perception of product quality.

These initiatives, in turn, enhance consumers' perceived product quality. (Page:26, Line:490-491)

This enables it to attract more consumers and to impose higher commissions on manufacturers.

This strategy allows the platform to expand its consumer base and levy higher commission rates on manufacturers. (Page:27, Line:508-509)

When the difference in consumers' perception of product quality is large, the benefits given to new consumers in the second period are negatively related to the probability of consumers obtaining high perception of product quality.

When the dispersion in consumers' quality perceptions is large, the second-period benefits offered to new consumers vary inversely with the likelihood that consumers perceive the product as high quality. (Page:33, Line:609-611)

We tried our best to improve the manuscript and made some changes marked in red in revised paper which will not influence the content and framework of the paper. We appreciate Editors/Reviewer’s warm work earnestly and hope the correction will meet with approval.

Thank you and best regards!

Yours sincerely,

Yingying Cheng

E-mail: 9906180@haust.edu.com
