# Supplementary material for: Two-period e-commerce platforms operation strategies considering the difference in product quality perception
Source: PLoS One. 2026 Mar 26;21(3):e0327280. doi: 10.1371/journal.pone.0327280 (PMC13020826; doi:10.1371/journal.pone.0327280)
Supplement: Attachment — Submitted filename: PONE-D-25-31916.R1.docx [file pone.0327280.s003.docx]

**Title:** Two-period e-commerce platforms operation strategies considering the difference in product quality perception

**Journal:** *PLOS ONE*

**Manuscript ID:** PONE-D-25-31916.R1

Authors answered all my previous comments very carefully. So, the paper can be accepted after these minor changes:

1. Paper is very lengthy, if possible, delete less important or repeated topics.
2. In my previous comment “Provide a table comparing the optimal numerical results for all scenarios.” Which is placed in Table 3 in the revised manuscript. In this comment, I want to show the results numerical values not expressions.
3. Conclusion section must be divided into main findings, managerial insights and limitations with future research directions.
