# Supplementary material for: Two-period e-commerce platforms operation strategies considering the difference in product quality perception
Source: PLoS One. 2026 Mar 26;21(3):e0327280. doi: 10.1371/journal.pone.0327280 (PMC13020826; doi:10.1371/journal.pone.0327280)
Supplement: Attachment — Submitted filename: Response_to_Reviewers_auresp_2.docx [file pone.0327280.s004.docx]

**Response to the Reviewer’s Comments**

**Manuscript ID:** PONE-D-25-31916R2

**Title:** Two-period e-commerce platforms operation strategies considering the difference in product quality perception

Dear Editors and Reviewers,

We appreciate the opportunity to revise our manuscript titled “Two-period e-commerce platforms operation strategies considering the difference in product quality perception” again to meet PLOS ONE’s publication criteria. We are very grateful to the editors and reviewers for giving us another opportunity to revise and improve our paper. Their comments are all valuable and very helpful for revising and improving our paper, as well as of the important guiding significance for our research. The following offers a comprehensive response to each of the comments. Revised portions are marked in red on the paper. Additionally, we have conducted a comprehensive revision of the entire manuscript. In this response letter, the comments are presented in italica, and our corresponding changes and additions to the manuscript are highlighted in red text. We have tried our best to make all revisions clear, and we hope that the revised manuscript meets the requirements for publication.

Here below is our description on revision according to your comments.

**Editor Comments:**

*1. Please remove your figures/ from within your manuscript file, leaving only the individual TIFF/EPS image files. These will be automatically included in the reviewer’s PDF.*

**Response:** Thank you very much for taking the time out of your busy schedule to review this paper again and provide valuable revision suggestions. we have removed our figures from within our manuscript file, leaving only the individual TIFF/EPS image files.

**The revised content is as follows:**

**Fig 5. Impact of differences in consumer quality perception on platform profit when platforms make sequential decisions.** (“─”indicates the platform profit in the com-mission-based model, “┄” indicates the platform profit in the self-managed model)

(Page32, Line:616-619)

**Fig 7. Impact of differences in consumer quality perception on manufacturers' profitability at platform sequential decision making.**

(Page34-35, Line:666-669)

**Fig 8. Operating mode selection for simultaneous decision making by the platform.** (“─”indicates the platform profit in the com-mission-based model, “┄” indicates the platform profit in the self-managed model)

**Fig 9. Leader platform operation mode selection when the platform makes successive decisions.** (“─”indicates the platform profit in the com-mission-based model, “┄” indicates the platform profit in the self-managed model)

**Fig 10. Follower platform operation mode selection when the platform makes successive decisions.** (“─”indicates the platform profit in the com-mission-based model, “┄” indicates the platform profit in the self-managed model)

**Fig 11. Platform operation mode selection.** (“─”indicates the platform profit in the com-mission-based model, “┄” indicates the platform profit in the self-managed model)

(Page36-37, Line:711-725)

Once again, thank you for your patience and professional support throughout the review process. Your comments have been instrumental in shaping our research into a more rigorous and impactful work. We sincerely hope the revised manuscript meets the journal’s standards and look forward to your further feedback.

*2. 1) Thank you for providing the following Data Availability Statement:*

*“All relevant data are within the manuscript and its Supporting Information files."*

*Before we proceed, we kindly ask that you please respond to the following queries in your "Author Comments" box:*

*1A) At this time, please confirm that your submission contains your "minimal data set", which PLOS defines as the data used to reach the conclusions drawn in the manuscript and any additional data required to replicate the reported study findings in their entirety. This includes:*

*- The values behind the means, standard deviations and other measures reported;*

*- The values used to build graphs;*

*- The points extracted from images for analysis.*

*If your submission contains the minimal data set, please confirm this in your "Author Comments" box and disregard the remaining queries (1B) and (1C).*

**Response:** Thank you very much for your valuable feedback. We confirm that our submission contains our "minimal data set" and we have added a data availability statement in the manuscript.

**The revised content is as follows:**

**Data Availability Statement**

All relevant data are within the manuscript and its Supporting Information files and we confirm that our submission contains our "minimal data set".

(Page43, Line:857-859)

We tried our best to improve the manuscript and made some changes marked in red in revised paper which will not influence the content and framework of the paper. We appreciate Editors/Reviewer’s warm work earnestly and hope that our paper meets the journal's publication requirements.

Thank you and best regards!

Yours sincerely,

Yingying Cheng

E-mail: 9906180@haust.edu.com
